# Quantum Chemical Insights into the Dissociation of Phenol: Shedding Light on Impact Ionization Mass Spectrometry for Icy Moon Exploration

**DOI:** 10.1021/acsearthspacechem.5c00318

**Published:** 2026-03-12

**Authors:** Thomas R. O’Sullivan, Partha P. Bera, Nozair Khawaja, Maryse Napoleoni, Bernd Abel, Frank Postberg

**Affiliations:** † Freie Universität Berlin, 9166 Institut für Geologische Wissenschaften, 12249 Berlin, Germany; ‡ 53406 NASA Ames Research Center, Moffett Field, Mountain View, California 94035, United States; § Bay Area Environmental Research Institute, Moffett Field, Mountain View, California 94035, United States; ∥ Institute for Space Systems, University of Stuttgart, Pfaffenwaldring 29, 70569 Stuttgart, Germany; ⊥ Institute of Chemical Technology, University of Leipzig, Linnéstraße 3, 04103 Leipzig, Germany; # J. Heyrovsky Institute of Physical Chemistry, Czech Academy of Sciences, Dolejškova 2155/3, 182 23 Praha, Czech Republic

**Keywords:** Enceladus, Europa, quantum chemistry, mass spectrometry, ice grains, icy moons, Europa clipper, Cassini, aromatic organics, LILBID

## Abstract

Ice grains emitted by the Saturnian moon Enceladus were sampled by Cassini’s Cosmic Dust Analyser (CDA) using impact ionization mass spectrometry. CDA revealed that Enceladus hosts a rich organic and inorganic chemical inventory in its subsurface ocean, hinting at its potential habitability. Analysis of fragmentation patterns with laser desorption experiments for the interpretation of CDA data has been essential; however, theoretical insights regarding both fragmentation and ionization processes are often missing. Here, we use density functional theory methods to investigate the energies for dissociation channels of phenol, a model aromatic compound for the features observed by CDA. The fragmentation channels are compared to experimental spectra obtained by using laser-induced liquid beam ion desorption (LILBID) mass spectrometry, an analogue for ice impact mass spectra. Our findings suggest that protonation is the dominant mechanism of ionization, that dissociation from the radical cation and neutral phenol molecule is limited, and that multiple isomers of the protonated molecule act as starting points for dissociation. The highest-intensity organic fragments observed in the LILBID spectrum—arising from the losses of CO, [M + H–CO]^+^, and water, [M + H–H_2_O]^+^—are found to be both thermodynamically and kinetically accessible. We examined water–molecule interactions during the initial production of the protonated molecule. The presence of water significantly influences the preferred site of protonation and causes variation in the relative energy ordering of the protomers. This work builds toward a computational model of ice grain impact ionization mass spectrometry, relevant for missions such as Europa Clipper and ESA’s L4 mission to Enceladus.

## Graphical abstract

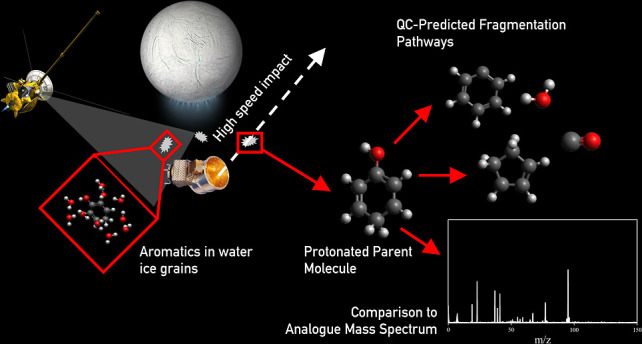

## 1 Introduction

The Saturnian moon Enceladus emits subsurface material from fractures in its crust near the south pole into space in a plume of ice grains and vapors.^1–4^ Such material originates from a global, saline subsurface ocean that lies beneath its icy crust.^5–8^ Enceladus possesses at least five of the six (CHNOP + S) elements considered essential for all known terrestrial life,^9–12^ placing it at as a key target for exploratory missions. Its astrobiological potential is further enhanced by the presence of ongoing water–rock interactions at the ocean–core interface, where hydrothermal activity is thought to be supported by tidal dissipation.^9,13,14^ The plume of Enceladus is the source of particles for Saturn’s E-ring, as a substantial fraction of ice grains escape the moon’s Hill Sphere and enter into Saturnian orbit.^15^ The sampling of ejected ice grains during the Cassini–Huygens mission offered remarkable insights into the subsurface composition of Enceladus. The Cosmic Dust Analyser (CDA) was an impact ionization mass spectrometer on board Cassini which generated time-of-flight (TOF) mass spectra via ice grain impacts upon its Chemical Analyser Target.^16^ Both during and since Cassini’s exploration of the Saturnian system, compositional analysis of ice grain mass spectra from CDA, alongside vapor-phase data from the Ion and Neutral Mass Spectrometer (INMS), has revealed evidence for a diverse subsurface chemical inventory, both organic and inorganic.^7–11,17–23^

CDA utilized impact ionization to generate ions via the kinetic energy supplied from impacts of ice grains onto its rhodium metal target. (Sub)­micron-sized dust or ice particles incident onto the target at hypervelocities (>2 km/s) disperse into an impact cloud of ions, electrons, neutrals, molecules, and macroscopic fragments. Molecular fragmentation induced by impact is thought to occur via the anisotropic dispersion of shockwaves as the impact cloud expands, with high-pressure regions contributing to bond-breaking events.^24–26^ Organic molecules embedded in ice grains can be partially shielded from dissociation upon impact via energy dissipation through surrounding water molecules from the ice shell, although these water molecules will exert additional mechanical forces, particularly in the case of polar organics which interact with the strong dipole moment of water.^27,28^ For lower-speed hypervelocity impacts (<8 km/s), additional dynamical and physicochemical factors also come into play, including impact angle, target surface effects, composition, water ice phase, and unique molecular structures.^27,29^ Organic fragments in mass spectra can also be suppressed by the presence of salts^30–33^ or can interfere with water clusters at the same *m*/*z* values.^20,34^

Aromatic compounds were detected in ice grains from Enceladus by CDA in Saturn’s E-ring and directly from the fresh plume as both fragments of larger marcomolecular organic structures and seemingly isolated molecules.^18,20,21^ The detection of a homologous series of peaks related to high-mass organic cations (HMOCs) at extended masses (>80 u) suggests the presence of organic species with increasing numbers of carbon atoms. The array of spectral features produced by the impact of an aromatic-bearing ice grain depends almost entirely upon the impact speed, although the aromatic ring remains detectable even at the highest impact speeds encountered by Cassini and the attached functional group(s).^18,20,21^ The phenyl cation (C_6_H_5_^+^) is the most common diagnostic feature for aromatic molecules in CDA mass spectra, but a coincident peak at *m*/*z* 91 occasionally appears, indicating the presence of the tropylium (C_7_H_7_^+^) cation. The ratio of intensities of the *m*/*z* 77 and *m*/*z* 91 peaks informs about the functional group attached to the aromatic ring. The formation of tropylium ions is energetically favorable when an alkyl group is attached, whereas phenyl cations often derive from non-alkyl substituents.^18^ In most HMOC spectra, however, high intensities of phenyl cations are observed,^35–37^ suggesting that the tropylium channel must in many cases be inhibited based on molecular structure and that non-alkyl substituents are instead common.

Isolated lower-mass aromatic compounds are also present in ice grains from Enceladus.^20,21^ A similar feature at *m/z* 77 is observed in characteristic low-mass aromatic spectra with further peaks occasionally observed at lower masses depending upon the impact speed of the ice grain responsible for the mass spectrum. Such aromatic features have been detected in both the E-ring and fresh plume ice grains, confirming that aryl groups originate from the Enceladus subsurface rather than as products of space weathering.^20,21^ Some mass spectra also demonstrate features related to *N*- and/or *O*-bearing aromatics, including those of both HMOC- and low-mass organic-rich ice grains, suggesting that multiple aromatic species may contribute to Enceladus’ organic inventory.^18,20^

The acceleration of ice grains in the laboratory is challenging, although such methods are beginning to emerge.^38^ In lieu of such accelerators, laboratory analogue techniques have been essential in the interpretation of mass spectra from Cassini’s CDA and for planning the science activities of Europa Clipper’s SUDA^39^ and ESA’s L4 mission to Enceladus. Laser-induced liquid beam ion desorption (LILBID) mass spectrometry replicates impact ionization mass spectra collected by spaceborne instruments through the laser irradiation of a μm-sized liquid water beam containing dissolved compounds.^34^ This results in the shockwave ablation of droplets into ions, fragments, and neutral compounds, the charged components of which can be directed into a TOF mass spectrometer.

While conventional mass spectrometric ion sources rely on direct ionization processes, LILBID operates in a fundamentally different way, whereby protonation and deprotonation events occur during the dispersion of the hot water matrix. Despite the physical differences between LILBID and impact ionization, the similarities in the mass spectrometric response between the two systems are striking. The LILBID process is characterized by several interacting mechanisms, each contributing to the overall outcome. Protonation and deprotonation dynamics compete within the transient, extremely hot water phase,^40–42^ while high-temperature chemical reactions and post-laser impact transformations can occur during rapid cooling.^43,44^ At higher laser fluences, plasma ionization and electron transfer processes, where genuine ionization processes take place, can occur.^43^ The extent of water clustering is influenced by the distribution of ions and neutral species in the expanding plume, alongside the efficiency of charge recombination.^26,34,43,44^ Similarly, ice particle impacts are not governed by a sole mechanism. Although the cold plasma generated during impact plays a central role, other factors—such as localized heating, transient high-pressure conditions, and secondary reactions—contribute further.^27,28,45–48^ Consequently, LILBID can replicate the spectral appearance of various velocity regimes of ice impact behavior by adjusting laser parameters, times of delayed extraction (i.e., gating of fast and slow ions), and microchannel plate potentials.^34^ This tunability supports the identification of experimental conditions that mimic ice impacts at specific kinetic energies, effectively enabling a calibration between LILBID and space-relevant impact energies, as has been demonstrated in previous studies.^30–32,34,49^

In the present work, we investigate the dissociative behavior of phenol, a model polar aromatic compound potentially representative of the singly substituted aromatic species prevalent in the Enceladus plume, verified by both CDA and INMS. The aromatic ring of monosubstituted aryls seems to obey similar fragmentation behavior in impact ionization mass spectrometry, regardless of the attached functional group.^20^ It is unclear whether phenol itself was detected in ice grains from Enceladus but, regardless, it offers insights into the generic dissociation behavior of O-bearing aromatics in impact ionization mass spectrometry. The following quantum chemical calculations address several processes, ranging from reactivity/fragmentation after simple protonation at increased temperatures to the conformational ensemble of microsolvated protonated phenol prior to ionization.^50,51^

Protonated molecules do not necessarily dissociate from the lowest-energy structure, but instead may fragment from multiple different contributing protomers, including the more energetically labile.^52,53^ We account for this by calculating the isomerization and tautomerization barriers between several different protonated isomers, as well as their relative thermodynamic energies. The extent of water clustering is generally coupled with impact speed; high speed impacts typically inhibit the formation of water clusters containing more than two H_2_O molecules.^34,54^ We thus consider noncovalent interactions at the highest impact speeds to be insignificant, whereas van der Waals’ interactions are thought to play a more important role at lower impact speeds.^54^

Modern quantum chemical methods can simulate the molecular fragmentation processes that occur during ionization from first principles.^55,56^ The collisional, destructive nature of ice grain impacts can induce complex fragmentation patterns that are challenging to predict through empirical approaches alone. Similarly, interpreting the vast quantities of data that will be collected by future instruments, such as Europa Clipper’s SUDA^39^ or HIFI^57^ for an Enceladus mission, could be aided by the generation of on-demand spectra from first principles. Jaramillo-Botero et al.^27^ used reactive molecular dynamics to study hypervelocity sampling of potential molecular biosignatures, employing a force-field based approach to model the effects of various parameters (impact angle, velocity, geometry, etc.) on the fragmentation of organic compounds. While this work provided valuable insights into the physics of hypervelocity impacts and the resulting influence on fragmentation, the use of more rigorous quantum mechanical treatment using, for example, density functional theory (DFT) can enrich these findings by more accurately capturing geometric and electronic structures, quantum effects such as correlation and dispersion, and bond-breaking events. Additionally, quantum chemical computations can easily provide definitive molecular structures and physical properties, such as dipole moments, charge distributions, and even optical molecular spectra. These facets may be useful in future work, but the scope of this study is to apply contemporary computational chemistry techniques to the analysis of a LILBID mass spectrum via the calculation of dissociative asymptotes.

## 2 Methods

### 2.1 Experimental Methods

LILBID mass spectrometry is proven to accurately replicate impact ionization mass spectra of ice grains in space. The setup is described in detail by Klenner et al.,^34^ so only the most salient details are given here. Phenol is dissolved in solution, at a concentration of 0.05 M, which is then injected into vacuum (5 × 10^–5^ mbar) as a narrow (12–20 μm diameter) liquid beam. This beam is intersected by a pulsed (20 Hz) infrared laser (2840 nm) at variable pulse energies up to 4 mJ, resulting in the absorption of the laser energy with a rapid, extreme temperature rise followed by mechanical, anisotropic dispersion of the beam into a cloud of atomic, molecular, and macroscopic fragments, both charged and neutral.^26,58^ Ions are accelerated toward a reflectron-type TOF mass spectrometer, which operates on the principle of delayed extraction, whereby a repelling electrode before the mass analyzer can be switched on after a predetermined period of time (delay time), allowing the selective detection of ions that arrive within a desired time. Varying the delay time and laser intensity allows for the simulation of ice grain impacts in space: more powerful intensities and lower delay times result in excessive fragmentation and the detection of only faster, lighter, organic ions.^34^

### 2.2 Computational Methods

We performed DFT calculations on several different phenol protomers, as well as fragments arising from each, using a modern range-separated ωB97M-V density functional and correlation-consistent polarized valence triple-ζ (cc-pVTZ) basis set. To investigate possible contributions from the direct dissociation of neutral and radical cationic phenol, we also calculate various dissociation channels from these structures. Molecular geometries were fully optimized, and harmonic vibrational frequencies were calculated to ensure a minimum on the potential energy surface (PES). This fully quantum treatment models bond behavior more accurately than semiempirical methods, including exchange–correlation and long-range dispersion corrections within the range-separated hybrid *meta*-GGA functional.^59^ We used the ORCA theoretical chemistry package^60–70^ on the Curta high performance cluster at the Freie Universität Berlin.^71^ ORCA invokes the Libxc variant of the ωB97M-V functional.^72^ The correlation-consistent polarized-valence triple-ζ (cc-pVTZ) basis set^73^ includes a set of 14 (3s2p1d) contracted atomic orbitals for hydrogen atoms, and a set of 30 (4s3p2d1f) contracted atomic orbitals for carbon and oxygen atoms. ORCA employs def2/J as an auxiliary basis set to enhance computational efficiency by approximating the products of orbital basis functions.^74^ Two-electron integrals are computed using the Libint2 library.^75^ We utilize the DFT-NL (nonlocal) dispersion correction to account for noncovalent (i.e., exchange correlation) interactions over large distances,^76,77^ particularly useful when considering microsolvation structures as described below. Final optimization runs utilized a 302-point Lebedev angular grid and a radial grid with 75 spatial points, increasing the number of integration points and offering an increase in accuracy. The dissociation energies were calculated as the difference between the total energies of parent species and the combined energies of the fragments after correcting for zero-point vibration energies.

To generate microsolvated structures, the DOCKER function within ORCA was used for the ideal placement of H_2_O molecules around each priorly optimized isomer, via the location of local minima on the PES by a swarm intelligence algorithm.^78^ The semiempirical tight-binding method GFN2-xTB^79^ is used with the DOCKER. Microsolvated structures obtained via the DOCKER are then further optimized to a higher level of theory first by using the cc-pVTZ basis set and ωB97M-V functional, and later with a tighter quadrature grid as described above for the gas-phase structures.

## 3 Results

The LILBID cation mass spectra of phenol simulating a high impact velocity (9–12 km/s)^34^ of ice grains in space are given in Figure 1. From quantum chemical calculations with ORCA, we then present an overview of the available dissociation channels and their computed energies for the different forms of phenol (for reasons explored in the Discussion section) that may contribute, via dissociation into charged fragments, to the mass spectra: neutral, radical cationic, and protonated. For the protonated structure, the lowest-energy conformers are computed, and the dissociation channels for isomerization from each are found. We compare dissociation pathways to elucidate the lowest-energy fragmentation channels for the neutral, radical cation, and several protonated isomers of phenol in the gas phase at 0 K, the merits of which are discussed later. We then explore explicit microsolvation, via the addition of water molecules, and perform further geometry optimizations for 1- to 4- and 10-membered water clusters, relevant for CDA mass spectra, which drastically alters both the energy and structure of the phenol isomers from those of the gas phase. The energies, vibrational frequencies, and optimized geometries—obtained via quantum chemical calculations for all chemical species mentioned in this work—are given in the quantum chemical data Supporting Information file available online.

**Figure 1:**
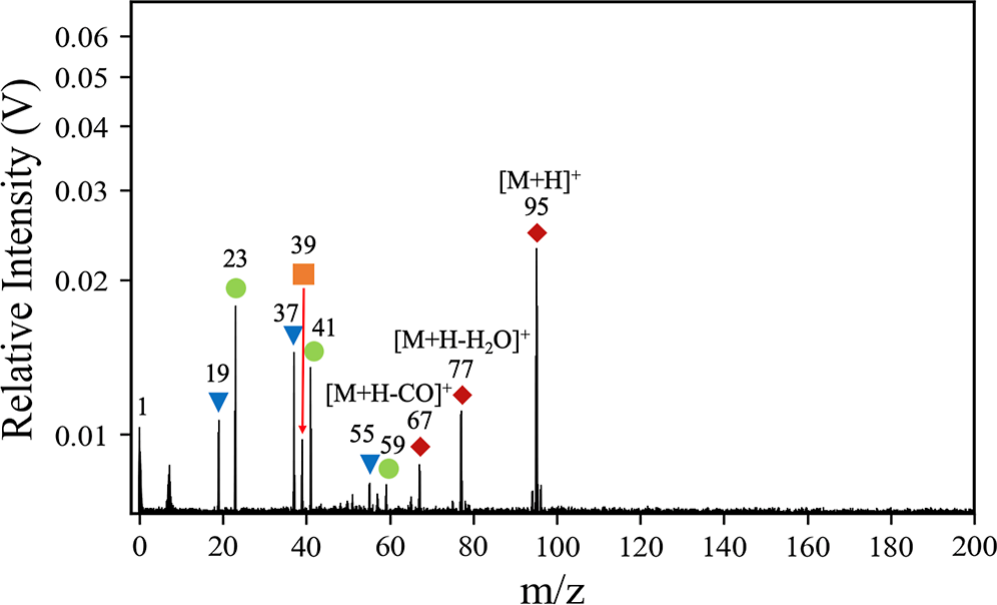
A LILBID cation mass spectrum of phenol simulating a high-velocity impact (9–12 km/s), obtained with a laser intensity of 100% and a delay time of 4.2 μs. The corresponding *m*/*z* value to each peak is printed on the spectrum. Blue triangle labels correspond to the standard patterns of water clustering of the form [H_3_O^+^(H_2_O)_*n*_]. Green circle labels correspond to sodium clusters of the form [Na^+^(H_2_O)_*n*_]. Orange squares correspond to the potassium ion K^+^. Red diamond labels correspond to organic-related features. Some peaks may have multiple contributing species. Minor peaks are not labeled.

### 3.1 Mass Spectra

We retrieved the LILBID cation mass spectrum of phenol dissolved in pure water from the mass spectral database outlined by Klenner et al.^80^ A laser intensity of 100% and a delay time of 4.2 μs simulate an impact speed of 9–12 km/s,^34^ henceforth referred to as the high-speed regime based on the range of velocities at which Cassini sampled Enceladean ice grains.^34,81^ Low (3–6 km/s) and intermediate (6–8 km/s) impact speeds are simulated in the LILBID mass spectra in Figures S1 and S2, respectively, while a mass spectrum of NaCl and some clustering peak intensity ratios are given in Figure S3 and Table S1, respectively, to aid in peak assignment.

The high-velocity spectrum (Figure 1) exhibits potential organic features related to phenol at *m*/*z* 67, 77, and 95. The base peak in the spectrum is the protonated molecular peak [M + H]^+^ at *m*/*z* 95. The peak at *m*/*z* 77 arises via the loss of water from the protonated molecule [M + H–H_2_O]^+^, i.e., the phenyl cation [C_6_H_5_]^+^. However, there is a clear contribution to this peak from sodium contamination in the LILBID mass spectrum simulating low (Figure S1) and intermediate (Figure S2) impact speeds. This is a known complication in the analysis of aromatic-type spectra from CDA^20^ and LILBID, although consideration of water/sodium cluster peak ratios and other LILBID mass spectra simulating alternative impact speeds can provide confirmation of the presence of an organic fragment at *m*/*z* 77 (see Figures S1 and S2 and Table S1 for more information). We note that the collision-induced dissociation mass spectrum of phenol also reveals a peak at *m*/*z* 77, alongside a peak at *m*/*z* 67.^82^ This peak at *m*/*z* 67 arises from the loss of CO from the protonated molecule [M + H–CO]^+^. Additional minor peaks are observed at *m*/*z* 39 (in all spectra), 57, and 75 (in the lower-speed spectra) due to K^+^ contamination and its water clusters.

### 3.2 Dissociation from Neutral Phenol and the Phenol Radical Cation

We computed a series of dissociation channels from both neutral phenol (Figure 2, Table 1) and the phenol radical cation (Figure 3 and Table 2), assuming that both dissociate from their electronic ground-state structures. From the neutral phenol molecule, we compute a number of fragmentation pathways, with anionic, neutral, and cationic products, some more plausible than others. Among the lowest-energy pathways are those involving the loss of a small neutral volatile, with losses of H_2_ and CO rather similar in energy. Each compound is stable as a neutral molecule, limiting charge transfer in these pathways. Indeed, as a general rule, all ionic dissociation pathways from neutral phenol require higher energies than fragmentation into neutral molecules. This fact, coupled with the high intensity of the protonated molecular peak in the LILBID mass spectrum, implies that direct dissociation from the neutral molecule plays a limited role. LILBID is blind to the presence of neutral fragments, unless such neutrals cluster with ions via ion–molecule interactions in the plasma cloud, so these computations provide further evidence that neutral unimolecular dissociation does not occur in LILBID.

**Figure 2:**
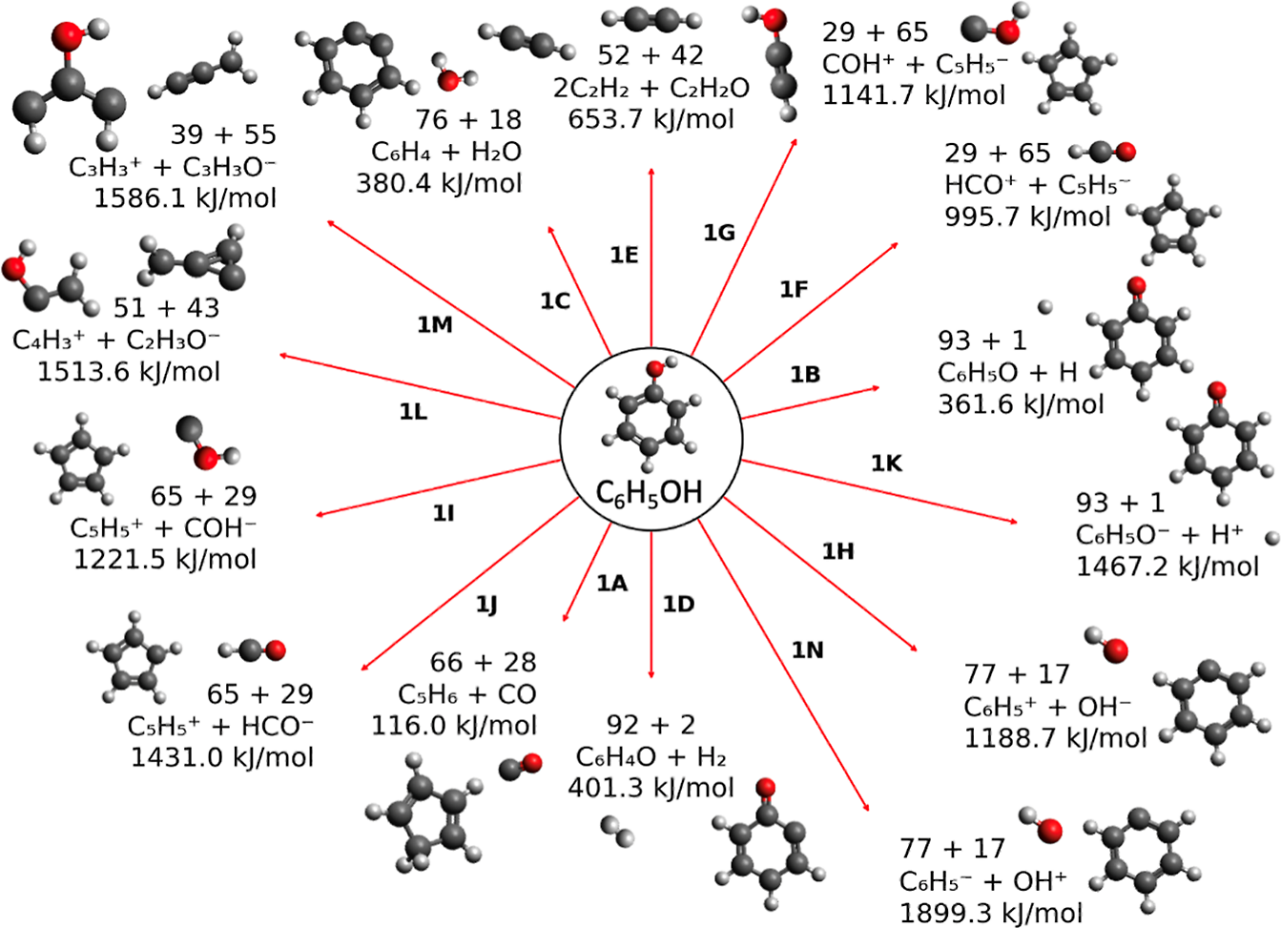
Dissociation channels from neutral phenol and their respective energies. The arrow lengths correspond to the path energy, with longer lines signifying higher energies for dissociation. Energies are given in kJ/mol on the figure for each pathway. The masses, in atomic mass units, of each product are printed above the formulas. Dissociation channels are labeled corresponding to the pathways in Table 1. Molecular structures, rendered in Avogadro^102^ v1.2.0, are printed for all dissociation channels.

**Table 1:**
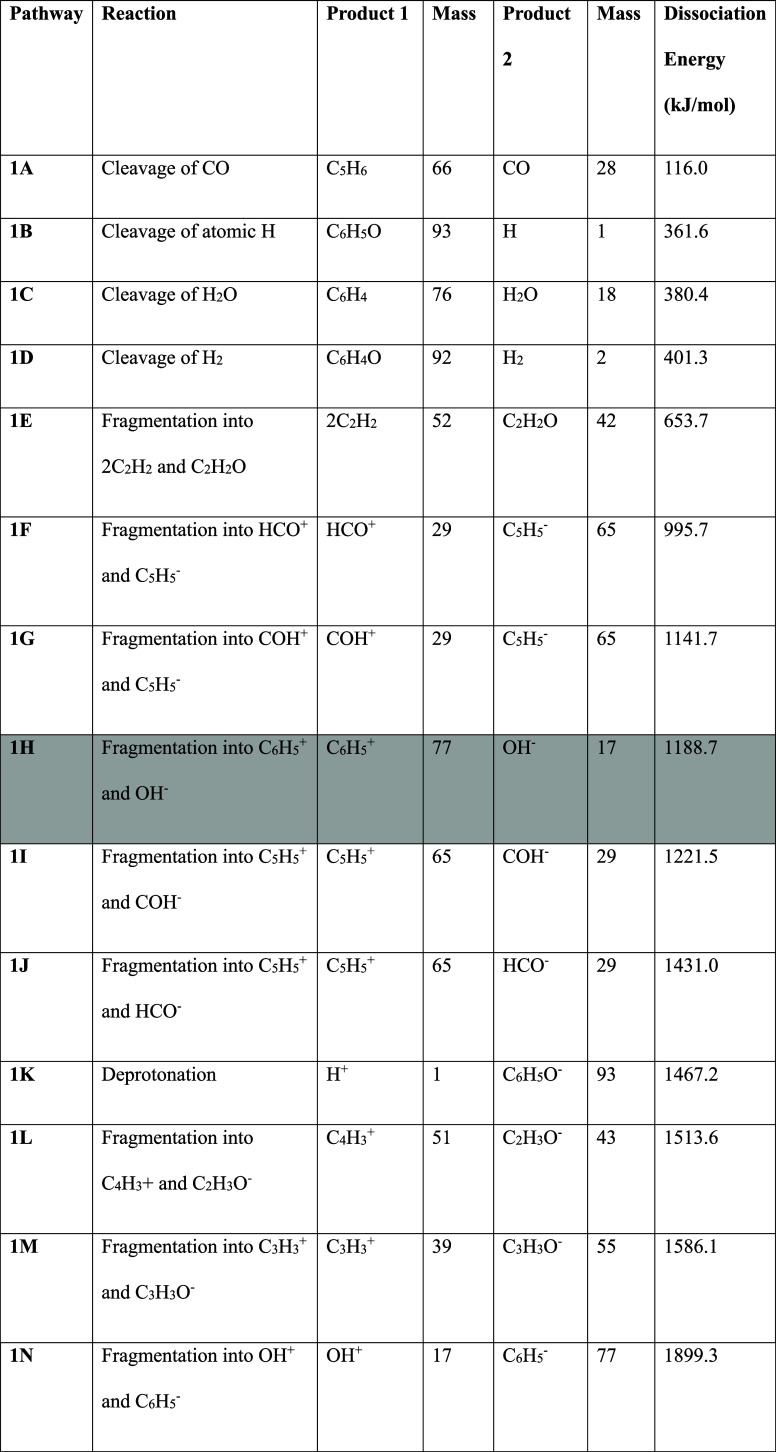
Dissociation Channels from Neutral Phenol and Their Respective Energies, with Product Formulae and Masses Also Given^a^

a Dissociation channels leading to cations that are observed in the LILBID mass spectra are shaded in grey.

**Figure 3:**
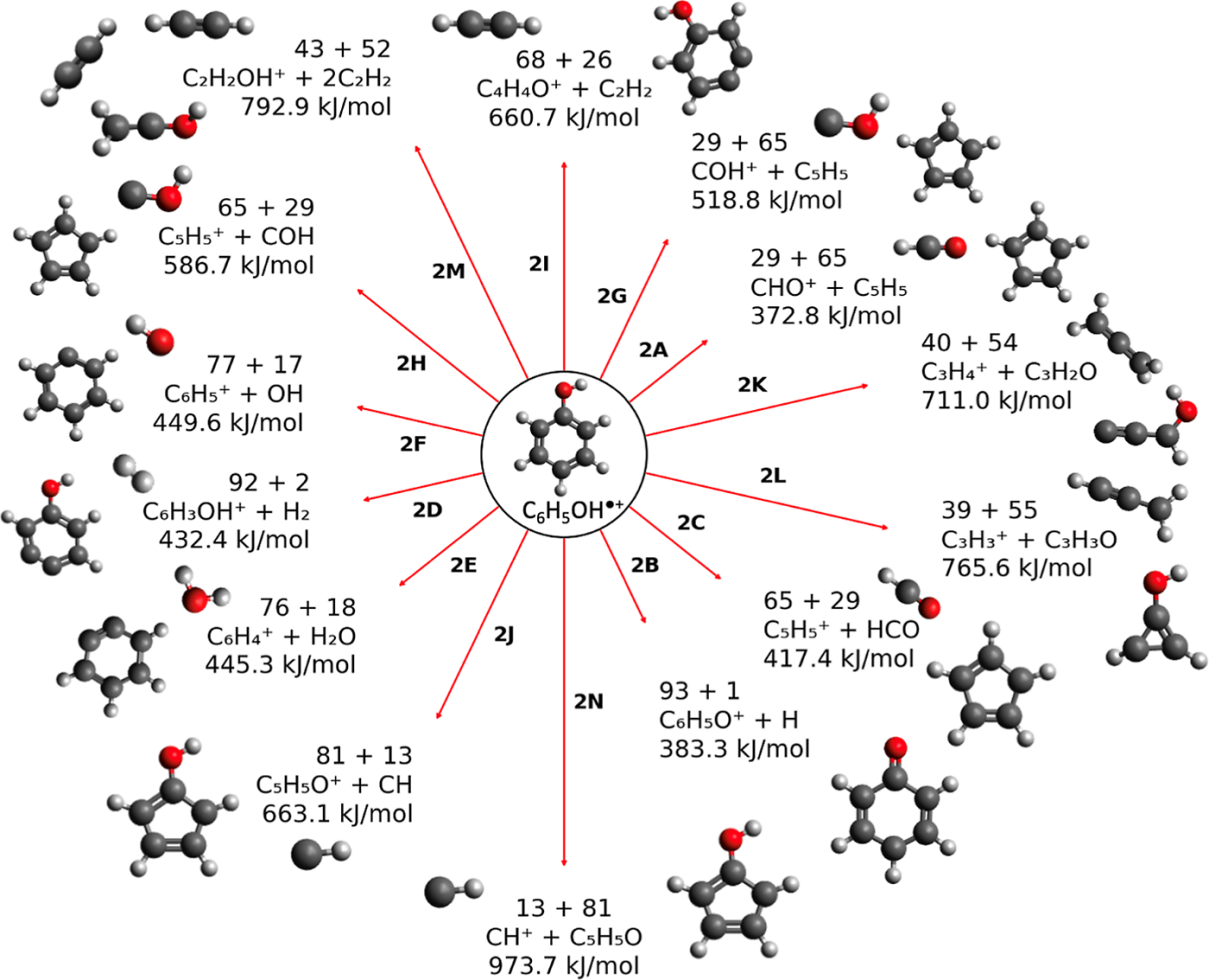
Dissociation channels from the phenol radical cation and their respective energies. The arrow lengths correspond to the path energy, with longer lines signifying higher energies for dissociation. Energies are given in kJ/mol on the figure for each pathway. The masses, in atomic mass units, of each product are printed above the formulas. Dissociation channels are labeled corresponding to the pathways in Table 2. Molecular structures, rendered in Avogadro^102^ v1.2.0, are printed on the figure.

**Table 2:**
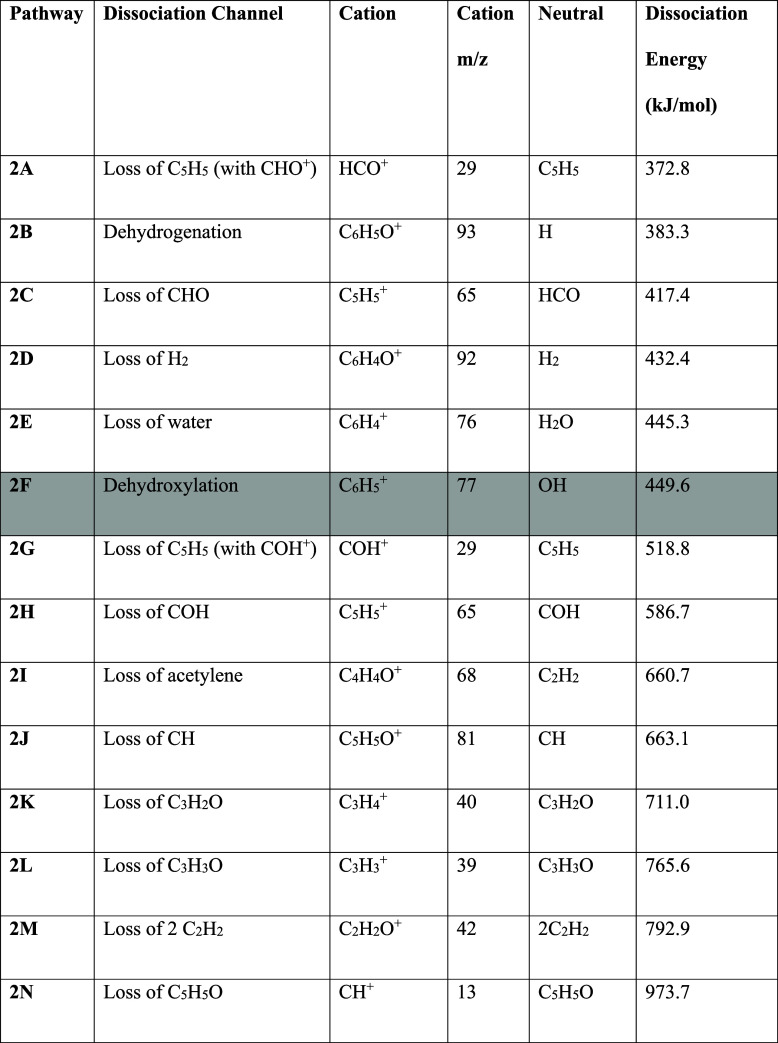
Relative Energies in kJ/mol of the Dissociation Channels from the Phenol Radical Cation, with all Energies Expressed Relative to the Phenol Radical Cation^a^

a Dissociation channels leading to cations that are observed in the LILBID mass spectra are shaded in grey.

We also assess various dissociation channels, illustrated in Figure 3 and Table 2, from the phenol radical cation. The lowest-energy pathway for dissociation of the phenol radical cation leads to the ion [COH]^+^, which is not observed in the LILBID mass spectrum, but is present in standard electron ionization (EI) mass spectra of phenol.^83^ This ion is an isomer of the more stable formyl ion, [CHO]^+^, which is probably the final structure following a rearrangement with a barrier of around 150 kJ/mol.^84^ In the presence of a water molecule, however, proton transfer to H_2_O is found to be more efficient than rearrangement,^85^ perhaps explaining its absence in LILBID mass spectra. Notably, the majority of dissociation channels from the phenol radical cation are lower in energy than those from the neutral molecule, suggesting that the radical cation is more prone to fragmentation. An intermediate-energy dissociation channel, the loss of OH leading to the phenyl cation at *m*/*z* 77, could contribute to this spectral feature here, although we cite this primarily as the loss of water from the protonated molecule. At a slightly higher energy, the loss of neutral CHO is an established channel of phenol fragmentation in the literature,^86^ but we do not observe a peak at *m*/*z* 65 related to this channel.

In general, we find the dissociation channels for the neutral and cationic phenol to be of little relevance to the observed LILBID mass spectrum, so we move to consider in detail the dissociation of protonated phenol.

### 3.3 Dissociation from Protonated Phenol

Protonation can occur at several different sites on the phenol molecule. We evaluate the relative energies for the possible sites of protonation. Figure 4 shows the relative energies for the different protonated phenol isomers. We find that *para*-protonated phenol is the lowest-energy isomer at 0 K in the gas phase, consistent with previous studies.^50,51,87,88^ The relative energies of the *ortho*- and *meta*-protonated phenols lie 18.5 and 71.9 kJ/mol, respectively, above that of the *para*-protonated isomer. *O*-protonated phenol is located 72.1 kJ/mol above the *para*-protonated isomer in the gas phase, with the least stable isomer, ipso-protonated, lying at 121.2 kJ/mol. We also include the *keto*- tautomer of phenol, cyclohexa-2,4-dien-1-one, which is important for some dissociation channels.

**Figure 4:**
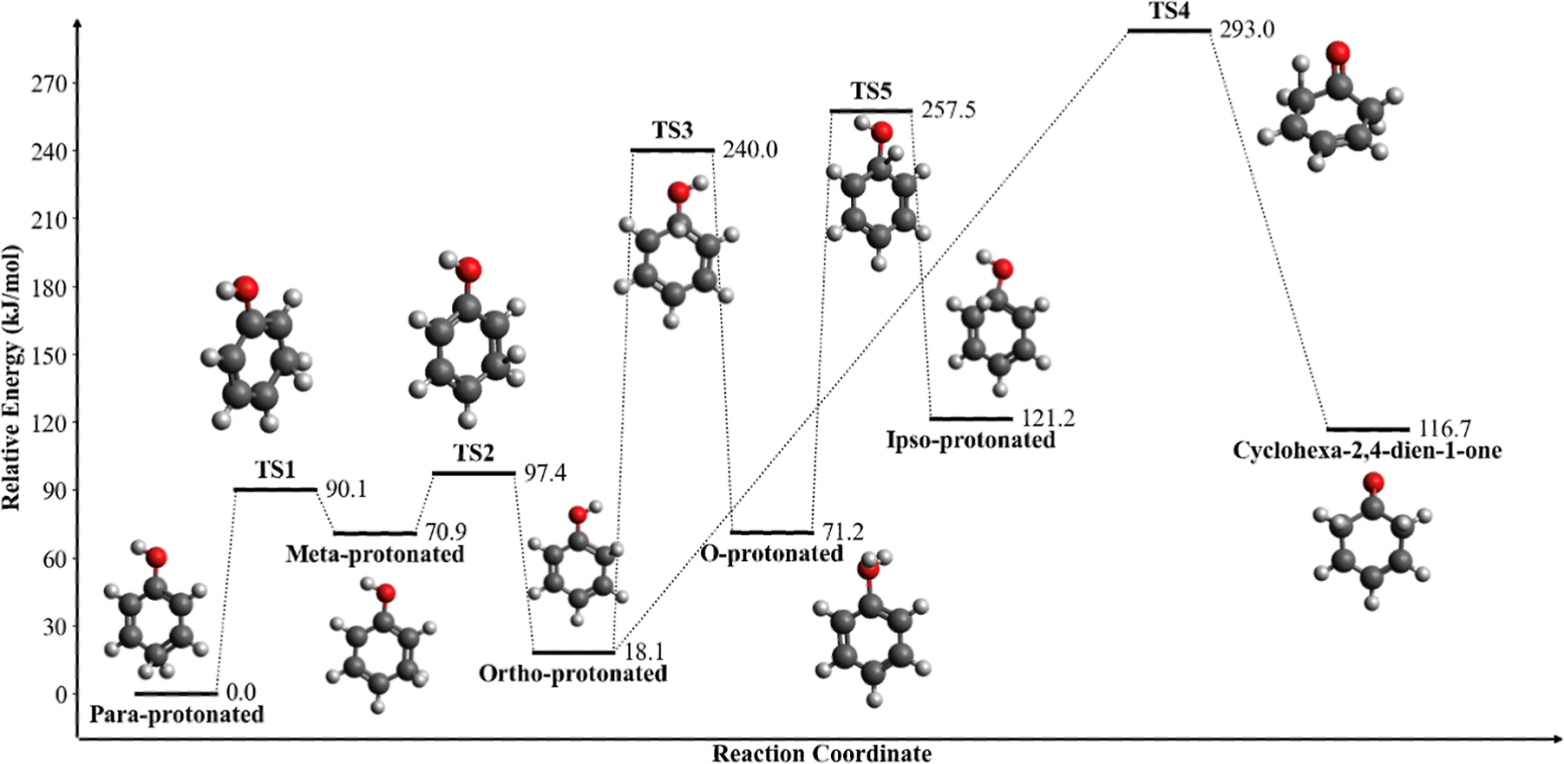
PES for protonated phenol isomers in kJ/mol and stepwise isomerization reaction pathways. Transition states are denoted “TS”. Relative energies are computed from *para*-protonated phenol. All energies include the zero-point energy correction. Molecular structures, rendered in Avogadro^102^ v1.2.0, are printed on the plot according to each species.

Our results indicate that *para*-protonated phenol is the most stable ground-state protonated isomer–protomer—in agreement with the literature.^50,51^ *Para*-protonated phenol exhibits the lowest dipole moment of all protonated structures, suggesting that this establishes a more symmetric charge distribution across the two polar parts of the molecule, with the concentrated positive charge at the protonated carbon disrupting the delocalized electron density across the aromatic ring. The ipso-carbon adjacent to the oxygen in *para*-protonated phenol is electron deficient, as our calculations show, and therefore, the C–O bond shrinks by 0.06 Å compared to that in *O*-protonated phenol. Relative energy ordering of the protomers, however, is significantly altered once water molecules are brought into the vicinity as explained below.

We also investigated the H^+^-transfer barriers for proton transfer between the various phenol protomers. These mechanisms involve stepwise hydrogen transfer involving transition states. We find that isomerization reactions are possible with relatively low barriers given by the transition-state structures for most protomers, with the exception of ipso-protonated phenol, which is characterized by an energy barrier of 186.3 kJ/mol (Figure 4). Protonated cyclohexa-2,4-dien-1-one is somewhat accessible from *ortho*-protonated phenol, suggesting that this keto- tautomer can, too, lead to additional dissociation channels that may otherwise be inhibited based upon structure.

We computed a number of possible dissociation channels from the phenol protomers, consistent with the principle that multiple different structures can contribute to the observed dissociation products of protonated molecules.^52^

Figure 5 shows the list of dissociation channels from each protonated phenol, with the energy from each printed on the graph and the *m*/*z* of the cationic fragment also shown. Two low energy dissociation pathways in agreement with the LILBID mass spectra are calculated: the loss of CO (leading to a peak at *m*/*z* 67 in Figure 1) and the loss of water (leading to the phenyl cation at *m*/*z* 77).

**Figure 5:**
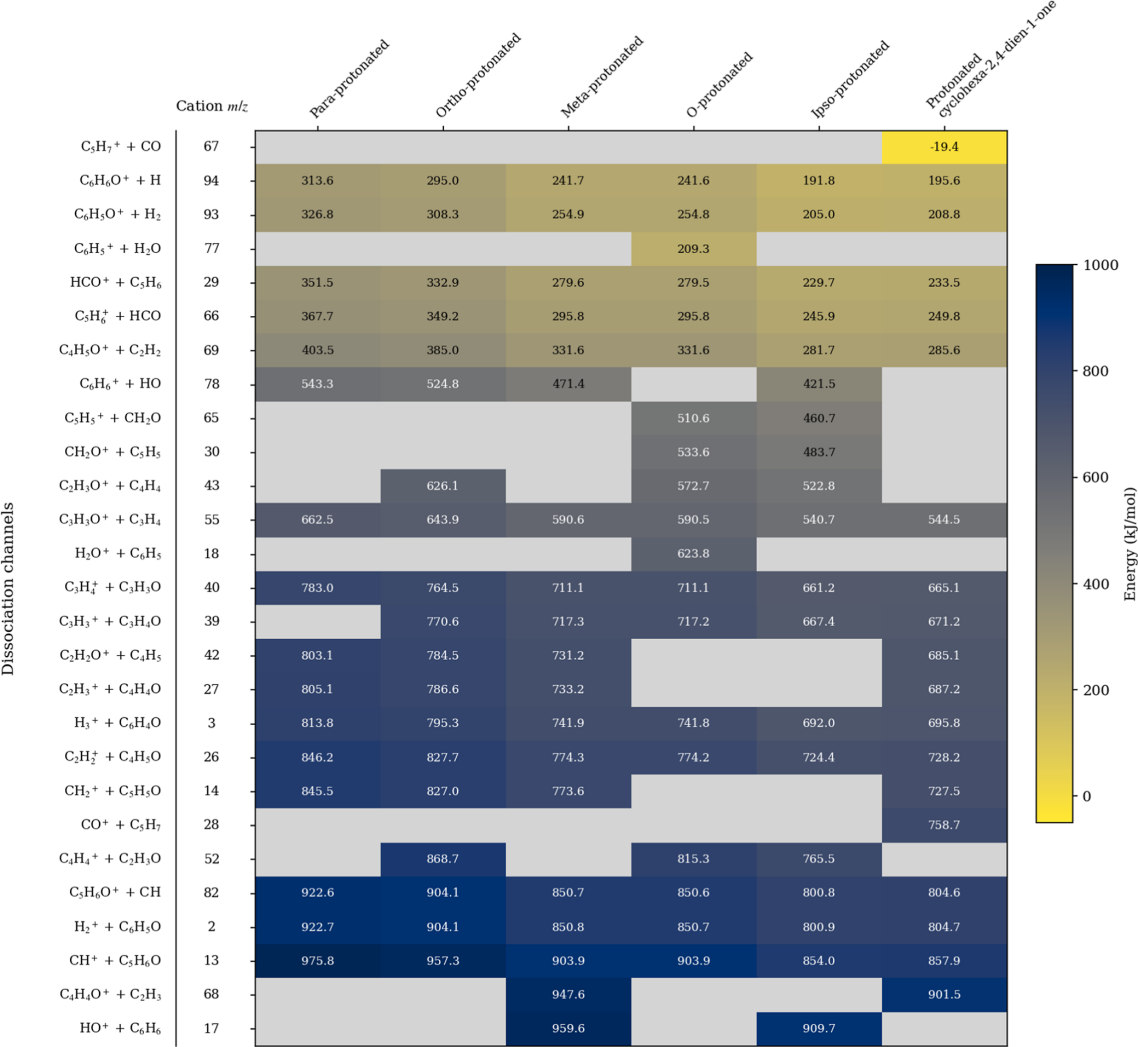
Dissociation channels from the various protomers of phenol and their respective thermodynamic energies. Lower energies appear in a lighter yellow color, while higher energies appear as a darker blue. Relative energies in kJ/mol for each channel are printed relative to each protomer. Blank entries represent structurally forbidden fragmentation pathways. Only dissociation channels below 1000 kJ/mol are included in this figure.

### 3.4 Effects of Surrounding Water Molecules on Protonation

Starting from the protonated structures shown in Figure 4, the relative energies of each protonated isomer in water clusters of 1, 2, 3, 4, and 10 H_2_O molecules are shown in Figure 6 and given in Table S2. Depending on the starting geometry, we find that increasing the number of water clusters added to the system changes the energetic preference for the most stable protonated isomer. In some cases, structures do not converge to stable minima; therefore, these are not included. We also include a structure where the proton has already been transferred to the water cluster, as this becomes increasingly thermodynamically favorable as the number of water molecules increases.

**Figure 6:**
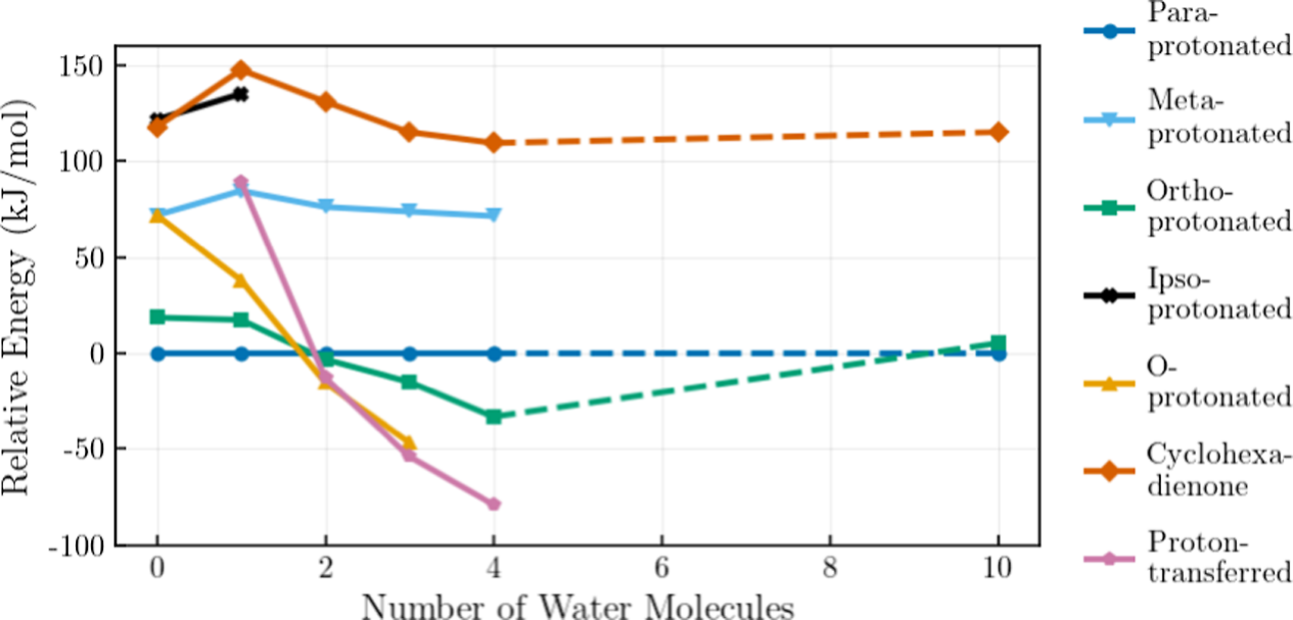
Relative energies in kJ/mol of different protonated structures calculated relative to *para*-protonated phenol with increasing numbers of water molecules. The dashed line represents an extrapolation over configurations that were not simulated. Structures that did not converge to minima, or converged to different structures, are excluded from the graph.

In general, water molecules form a cluster network originating from the hydroxyl part of the phenol molecule and avoiding the hydrophobic π-bonded regions above and below the aromatic ring. We observe that both the site of protonation and the number of explicit water molecules significantly influence the energetic landscape. The energetically favorable structure for protonated phenol varies with an increasing number of water clusters (Figure 6). *Para*-protonated phenol remains the lowest-energy structure in the presence of one water molecule, but other structures become more favorable as more are added. In particular, the abstraction of the proton into the water cluster network becomes the preferred configuration for *n* ≥ 3, in agreement with results in the literature. Charge becomes held in the water cluster network and may lead to alternative routes of dissociation. *O*-protonated phenol is the only protomer that approaches the energy of the proton-transferred configuration for *n* = 3.

## 4 Discussion

Comparison of the PES for phenol protomers shown in Figure 4 with the set of dissociation channels in Figure 5 reveals that proton transfer barriers are competitive prior to any dissociation. This enables straightforward losses of neutral molecules during fragmentation without the need to invoke complex reaction mechanisms. Wiersma et al.^89^ observed that supplying energy to the phenyl cation, [C_6_H_5_]^+^, typically resulted in intramolecular structural changes prior to dissociation, limiting the thermodynamic availability of some dissociation channels and enhancing that of others. Their work showed that such isomerization steps drastically expanded the product space, meaning that fragments formed via many different pathways may contribute to the observed mass spectra. Reis et al.^52^ also provided a guide for studying the dissociation of protonated molecules and highlighted the importance of considering more labile protomers than simply the lowest-energy structure. This has been shown to be critical in the identification of dissociation channels from protonated aniline.^90^

The most prominent feature corresponding to a fragment in the LILBID mass spectra (Figures 1, S1, and S2) is the peak at *m*/*z* 77, attributed to [C_6_H_5_]^+^. Notably, this channel is directly accessible only from the *O*-protomer, from which the dissociation proceeds without an activation barrier. While the CO- and H_2_-loss channels are lower in thermodynamic energy, each pathway must pass through a higher activation barrier. The rate-limiting step for the loss of CO is an initial proton transfer with a barrier of 187.3 kJ/mol, while the only transition state for the loss of H_2_ is characterized by a barrier of 403 kJ/mol (Figures S4 and S5, respectively). This high barrier for H_2_-loss explains the absence of any cationic fragment at *m*/*z* 93 in the LILBID mass spectra. The loss of H_2_O is only accessible from *O*-protonated phenol, disregarding the possibility of some complex rearrangement from a different protomer. This dissociation was found to be barrierless; H_2_O readily departs the molecule when the C–O bond is stretched.

H-loss from the protonated molecule is thermodynamically facile but would result in the formation of the unstable phenol radical cation, which could undergo further unimolecular dissociation. Fragments corresponding to any channels below 449.6 kJ/mol (loss of OH leading to *m*/*z* 77, Figure 3 and Table 1) from that structure are not observed in the LILBID spectrum, suggesting that this does not occur.

Of the thermodynamic dissociation energies, three of the four lowest correspond to fragment pairs that minimize disruption of the cyclic structure. This is consistent with the recurrent appearance of the phenyl cation ([C_6_H_5_]^+^ at *m*/*z* 77) in the CDA mass spectra of ice grains containing monosubstituted aromatic compounds. The one exception here is the loss of CO, which can proceed only via protonated cyclohexa-2,4-dien-1-one, which must either be a contributor to the protomer ensemble or exist as an intermediate along the dissociation pathway. Given that this structure possesses a rather high energy amidst all of the microsolvated protomers and that it requires the rearrangement of a H atom more tightly bound than the proton, the latter may be more likely. From cyclohexa-2,4-dien-1-one, however, there is a thermodynamic drive toward the products, which are lower in energy than those of this structure. We also calculated the full reaction pathway for the loss of CO from protonated phenol. The loss of CO from the phenol radical cation has previously been investigated in some detail,^91,92^ so we explored a structurally similar pathway for protonated phenol, whereby CO is initially forced out of the aromatic ring. However, we find that an additional intermediate characterized by a proton transfer from the *ortho*- to *meta*-positions is required. From this protonated ketone, CO is forced out of the ring, which closes to become five-membered, but remains bonded in the second intermediate. CO then begins to depart the ring via a second transition state before fully detaching. See Figure S4 for detailed information on this pathway. Several searches for the loss of CO from other protomers were conducted, which would involve concurrent H-migration, but no additional pathways could be identified.

The most energetically favorable fragmentation pathways (as shown in Figure 5, in order of decreasing energy: the losses of CO, H, H_2_, H_2_O, C_5_H_5_, HCO, C_2_H_2_, OH, and CH_2_O between −19.4 and 510 kJ/mol) are dominated by the losses of small and stable neutrals, with the exception of C_5_H_5_/HCO^+^ which, as discussed above, requires a post-dissociation rearrangement that is questionable under LILBID conditions. These low energies of dissociation are not surprising; protonated molecules in the gas phase preferentially undergo such unimolecular losses of neutral molecules, leaving behind a stable ion.^52^ An exception here is the loss of H, releasing a neutral that is itself unstable. While the phenyl cation formed from the loss of water is not an inherently stable ion, the dissociation is barrierless and readily proceeds.

Despite the thermochemical differences between our quantum chemical calculations and ice grains in the Cronian system, comparison of our dissociation channels with the ions observed in LILBID spectra offers insight into both the strengths and limitations of our approach thus far. Both the impact and laser desorption processes generate extremely high instantaneous temperatures, and the response of specific molecules to this could be modeled using ab initio molecular dynamics simulations.^93^ While we observe fragments in the LILBID spectrum that correspond to low-energy dissociation channels, little quantitative consideration has hitherto been given to peak intensities. These aspects will form the basis of future work; as multiple physicochemical interactions occur during LILBID and ice grain impacts, the disentanglement of peak intensities requires a more rigorous approach. An analysis of low-lying conformers would also allow for a prescreening of expected fragmentation pathways, facilitating the selection of a small number of the most relevant starting geometries for the further time evolution of newly generated fragments using ab initio molecular dynamics. Empirically, IR photodissociation spectroscopy experiments^50^ to identify the collection of protomers that may go on to dissociate in LILBID mass spectrometry would be valuable to assess the availability of various dissociation channels and assess trends across functional groups.

In this work, we discount secondary fragmentations of the products. In general, one would expect multistep dissociations to be trackable via a sequence of organic peaks decreasing in both intensity and mass. Limited numbers of organic peaks are observed at low *m*/*z* values (albeit some may be obscured by the presence of water and salt clusters); however, at least in the relatively simple molecule phenol, one-step pathways seem to be more significant. This is likely not the case for larger molecules, as Grimme^56^ observed that secondary ion fragmentations play a significant role in simulations using a quantum model for EI mass spectrometry.

In both ice grains from Enceladus and the LILBID analogue technique, organic solutes are shrouded in multiple layers of water clusters.^93^ This is particularly evident in LILBID mass spectra, where organic fragment–water clusters are frequently formed. The presence of water molecules likely influences the site of protonation. Indeed, in the case of phenol, we observe that while the C4-protonated molecule is the lowest energy structure, the presence of 1–10 water clusters leads to protonation at the OH-group. Evidently, dissociation channels cannot necessarily be computed from the lowest-energy dehydrated structure as would be the case for gas-phase reactions; instead, water molecules play a pivotal role in the “starting” arrangement needed to calculate dissociative pathways for LILBID (and impact ionization), as well as the physicochemical nature of such pathways. This is a limitation of our work; interactions between water molecules, protonated molecules, and organic fragments will influence the availability of, and energy barriers for, dissociation channels. Indeed, we do not attempt to model the complete desorption, protonation, and fragmentations processes. Our results here serve as a guide for predicting and interpreting mass spectra from LILBID and, by extension, the impact ionization mass spectra for which it is an analogue. We calculate the mechanisms and energy barriers for the most relevant dissociation channels, but it is possible that some unexpected pathways also play a role.

The dissociation pathways available to aromatic compounds such as phenol are not only significant in the context of impact ionization and fragmentation. Chemical reactions at some hydrothermal sites on Earth’s seafloor, thought to be similar to those found at Enceladus’ ocean–core interface,^94,95^ can enable the substitution of functional groups on the aromatic ring into more stable moieties.^96,97^ Understanding these reactions, albeit at distinct physicochemical conditions to those considered in this work, will shed light on the most likely aromatic compounds responsible for characteristic features in CDA mass spectra.^18,20,21^ At the ocean surface, despite the fact that freeze–thaw cycles of the organic material in water can enable organic chemistry,^98^ aromatics are not expected to be significantly altered at these sites owing to the stability and hydrophobicity of the aromatic ring. This behavior parallels previous observations in terrestrial polar environments, where aromatic compounds concentrate at the top of the unfrozen water column beneath the nascent ice layer.^99^ Even in the presence of highly reactive species such as OH radicals, the reactivity of aromatic compounds appears to be suppressed due to self-association effects.^100,101^ The coupling of thermodynamic modeling^103^ of organics in the Enceladean subsurface with quantum chemical interpretations of spacecraft data would offer a comprehensive overview of the origin, evolution, and detection of biologically relevant compounds by future missions. SUDA will also detect negatively charged ions, and the preference of molecules to dissociate into a mixture of positive and negative ions should be explored.

## 5 Conclusions

This work employs a theoretical chemical approach to interpreting the LILBID-TOF mass spectra of phenol, as an analogue for spaceborne impact ionization mass spectrometry. Deciphering the behavior of aromatic organic compounds in impact ionization mass spectra is of importance in both the context of Enceladus, where Cassini detected polar phenyl- and benzoyl-like aromatic derivatives in ejected ice grains, and future exploration of other icy moons. In this work, phenol is investigated as a model polar aromatic compound using LILBID mass spectrometry, which replicates impact ionization mass spectra of ice grains in space, in combination with quantum chemical calculations at the cc-pVTZ/ωB97M-V level of theory. The lowest-energy dissociation channels from neutral, radical cationic, and various protonated isomers of phenol in the gas phase were investigated in order to gain insight into dissociation. Microsolvation effects on the protonated molecule and the associated structural differences were also investigated. We find that *para*-protonated phenol is the lowest-energy protonated structure in the gas phase, but in the presence of explicit water molecules, proton transfer events, both intramolecular and through the water cluster network, may occur, rendering *O*-protonated phenol more favorable. The lowest-energy dissociation channels from the set of phenol isomers (loss of CO—[M + H–CO]^+^, loss of water—[M + H–H_2_O]^+^) match the organic fragment ions that appear at *m*/*z* 67 and 77, respectively. Reaction mechanisms are evaluated for key pathways, suggesting a barrierless dissociation of H_2_O from *O*-protonated phenol and a low barrier for the loss of CO via a number of steps. The theoretically computed dissociation channels from neutral phenol and the phenol radical cation, however, do not correspond as well to the LILBID spectrum, hinting at a protonation-first mechanism for both LILBID and impact ionization. Peak intensities are somewhat correlated with channel energies and may depend upon the available energy along the dissociative mode. This suggests that further work to model energy transfer in ice grain impacts and laser irradiation into organic molecules and the surrounding water matrix is needed. Furthermore, molecular geometries in this work were computed at 0 K, with no thermal corrections applied to energies. In reality, an ensemble of low-lying conformers may constitute starting points for dissociation, and we consider only a few isomers of protonated phenol toward this aspect. A full PES scan for low-energy structures would enable a more realistic model, and a statistical physics approach could be applied to the dissociation channels accessible from each. Future work will extend to other singly substituted aromatics, such as benzoyl-like compounds, which are also candidates for the observed features in CDA spectra. Similarly, the presence of water molecules significantly deviates the ground-state behavior of protonated phenol, which will also be considered in future work.

## Supplementary Material





## Notes

Section 1: additional LILBID spectra and peak ratio analysis; Section 2: reaction mechanisms; and Section 3: quantum chemical data for explicit solvation quantum chemical data (PDF)
List of energies, frequencies, and geometries for all structures (PDF)

## Abbreviations

CAT: Chemical Analyser Target
cc-PVTZ: correlation-consistent polarized valence triple-ζ
CDA: Cosmic Dust Analyser
DFT: density functional theory
EI: electron ionization
ESA: European Space Agency
HIFI: High Ice Flux Instrument
HMOC: high-mass organic cation
HPC: high-performance cluster
INMS: Ion and Neutral Mass Spectrometer
LILBID: laser-induced liquid beam ion desorption
NL: nonlocal
PES: potential energy surface
SUDA: SUrface Dust Analyzer
TOF: time-of-flight
