# Supplementary material for: Quantum Chemical Insights into the Dissociation of Phenol: Shedding Light on Impact Ionization Mass Spectrometry for Icy Moon Exploration
Source: ACS Earth Space Chem. 2026 Mar 12;10(4):1033–46. doi: 10.1021/acsearthspacechem.5c00318 (PMC13093666; doi:10.1021/acsearthspacechem.5c00318)
Supplement: Supplementary file 1 [file sp5c00318_si_001.pdf]

Supporting Information File 1

Quantum Chemical Insights into the Dissociation of Phenol: Shedding light into Impact Ionisation Mass Spectrometry for Icy Moon Exploration

Thomas R. O'Sullivan,[1] Partha P. Bera,[2,3]* Nozair Khawaja,[1,4] Maryse Napoleoni,[1] Bernd Abel,[5,6] Frank Postberg[1]*

[1]Freie Universität Berlin, Institut für Geologische Wissenschaften, Malteserstr. 74-100, 12249 Berlin, Germany.

[2]NASA Ames Research Center, Moffett Field, Mountain View, California, USA 94035.

[3]Bay Area Environmental Research Institute, Moffett Field, Mountain View, California, USA 94035.

[4]Institute for Space Systems, University of Stuttgart, Pfaffenwaldring 29, 70569 Stuttgart, Germany.

[5]Institute of Chemical Technology, University of Leipzig, Linnéstraße 3, 04103 Leipzig, Germany.

[6]J. Heyrovsky Institute of Physical Chemistry, Czech Academy of Sciences, Dolejškova 2155/3, 182 23 Praha, Czech Republic.

*Corresponding authors: tr.osullivan@fu-berlin.de, partha.bera@nasa.gov

Supporting Information File 1

1. Additional LILBID Spectra, Peak Ratio Analysis.
2. Reaction Mechanisms.
3. Quantum Chemical Data for Explicit Solvation.

Section 1. Additional LILBID Spectra and Peak Ratio Analysis.

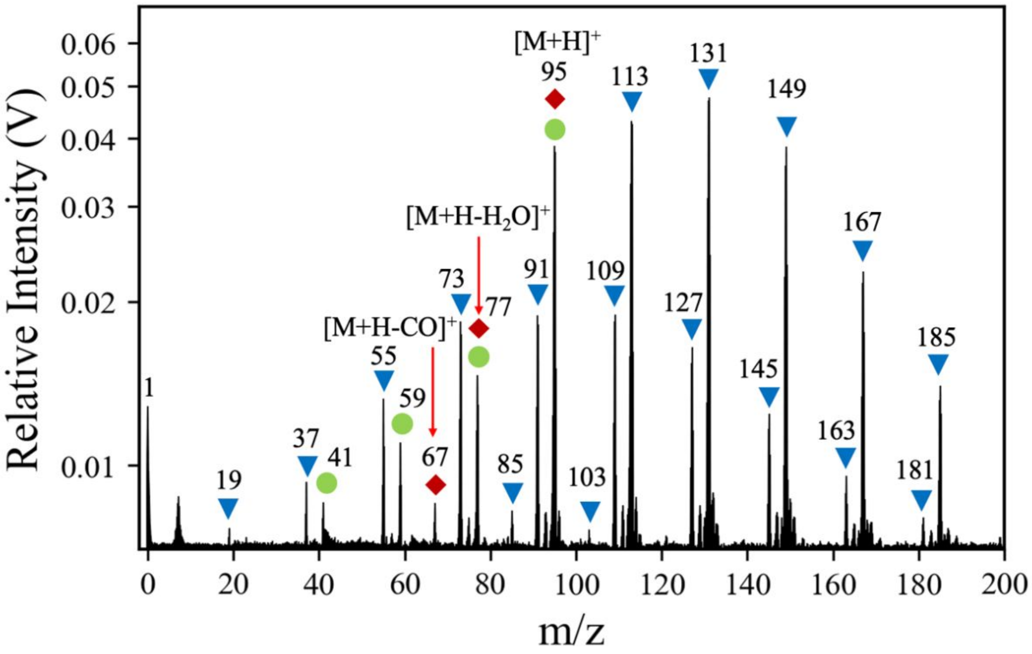

SI Figure S1. A LILBID cation mass spectrum of phenol simulating a low-velocity impact (3-6 km/s). Extensive water and sodium clustering up to high masses can be observed. Red diamonds represent organic features, which are the only species labelled by m/z and formula. Blue triangles represent pure water clusters of the form [$H_3O^+(H_2O)_n$] and water clusters of organic species. Green circles represent sodium clusters of the form [$Na^+(H_2O)_n$]. This mass spectrum of phenol dissolved in pure water from the mass spectral database outlined by Klenner et al.[80]

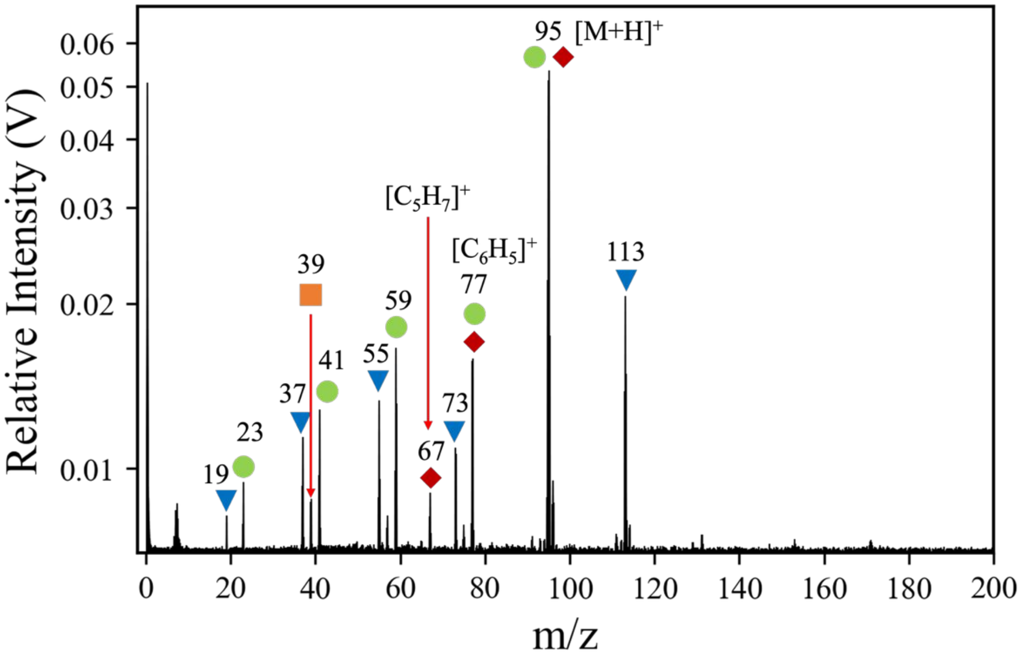

SI Figure S2. A LILBID cation mass spectrum of phenol simulating an intermediate-velocity impact (6-8 km/s). Water and sodium clustering can be observed. Red diamonds represent organic features, which are the only species labelled by m/z and formula. Blue triangles represent pure water clusters of the form $[H_3O^+(H_2O)_n]$. Green circles represent sodium clusters of the form $[X^+(H_2O)_n]$. Orange squares represent the potassium ion K^+. This mass spectrum of phenol dissolved in pure water from the mass spectral database outlined by Klenner et al.[80]

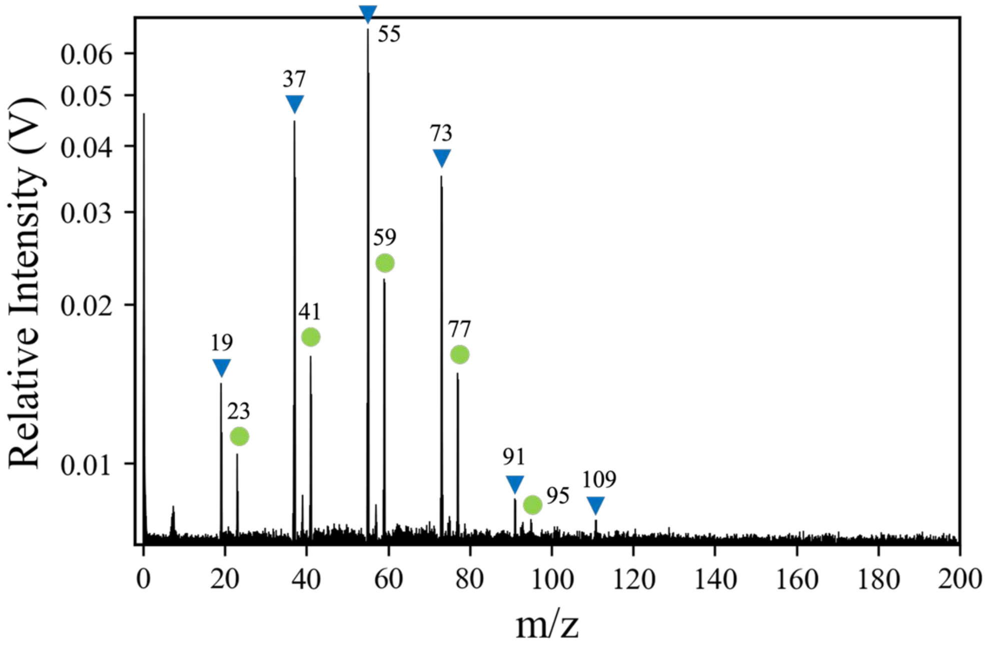

SI Figure S3. A LILBID cation mass spectrum of 10^{-6} M NaCl dissolved in water simulating an intermediate-velocity impact (6-8 km/s). Standard patterns of water and Na-water clustering are observed. Blue triangles represent pure water clusters of the form $[H_3O^+(H_2O)_n]$. Green circles represent sodium clusters of the form $[X^+(H_2O)_n]$. Note the significant reduction in intensity between the peaks at m/z 59 and 77. This spectrum was retrieved from the water from the mass spectral database outlined by Klenner et al.0

	**Peak ratios**			
**Simulated Impact Speed**	**19/23**	**37/41**	**55/59**	**73/77**
High speed (Main Fig. 1)	0.5981	1.0702	1.0057	0.6462
Intermediate speed (SI Fig. S2)	0.8679	0.8904	0.7994	0.6855
Low speed (SI Fig S1)	1.0377	1.0919	1.2029	1.2572

SI Table S1. A comparison of the peak ratios for H_2O and Na cluster series that may interfere with the identification of organic peaks in the LILBID mass spectrum, for $[X(H_2O)_{n=0,1,2,3}]$ where X is H_3O^+ or Na^+. If no organic fragments contribute to these masses, the ratios should remain largely constant – which is not the case here. Note that the peaks at m/z 23 is not shown in the low-speed spectrum as its low intensity precluded retention in processing. The anomalous relationship between m/z 19 and 23 in the high-speed spectrum can be explained by sodium contamination in the measured sample, which does not form clusters as efficiently at higher laser intensities.

Section 2. Reaction Mechanisms

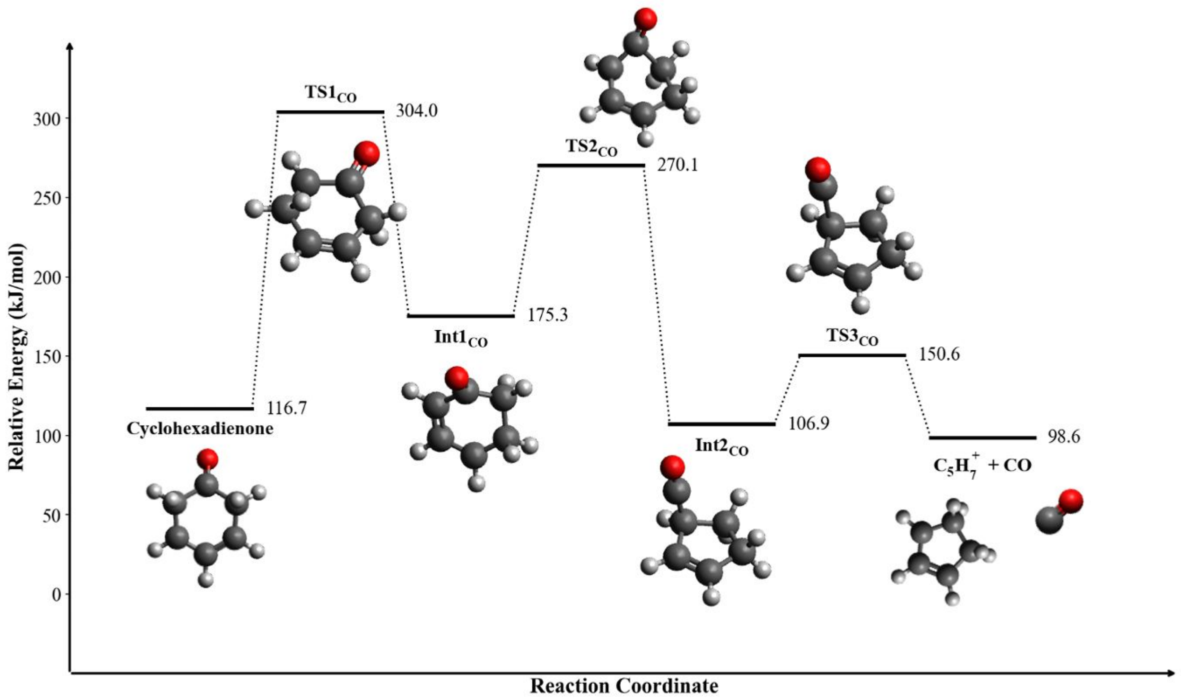

SI Figure S4. A reaction coordinate diagram for the loss of neutral CO from cyclohexa-2,4-dien-1-one.

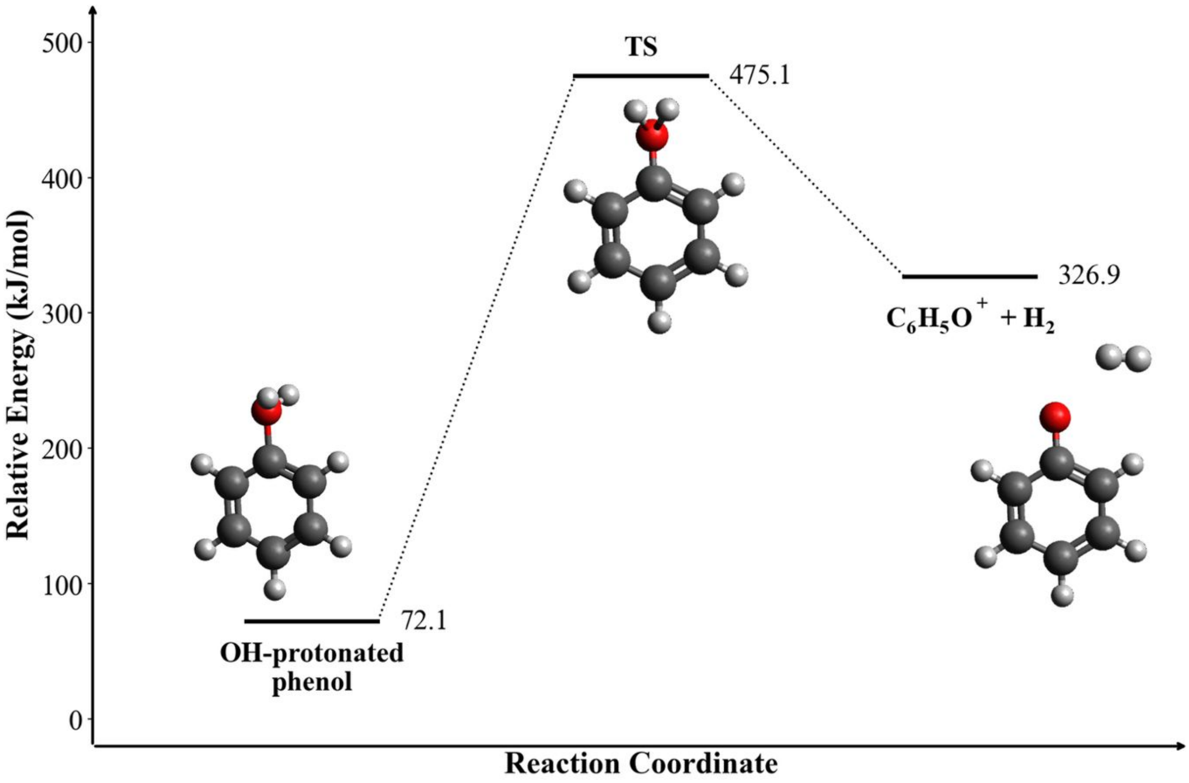

SI Figure S5. A reaction coordinate diagram for the loss of neutral H_2 from O-protonated phenol.

Section 3. Quantum Chemical Data for Explicit Solvation

n **H_2O**	**Structure**	**Relative Energy (kJ/mol)**	**Notes**

**0**	Para-protonated	0	
	Meta-protonated	71.9	
	Ortho-protonated	18.5	
	Ipso-protonated	121.8	
	O-protonated	72	
	Cyclohexa-2,4-dien-1-one	118	
**1**	Para-protonated	0	
	Meta-protonated	84.6	
	Ortho-protonated	17.3	
	Ipso-protonated	135.2	
	O-protonated	37.9	
	Cyclohexa-2,4-dien-1-one	147.6	
	Proton-transferred	89.5	
**2**	Para-protonated	0	
	Meta-protonated	76.2	
	Ortho-protonated	-3.2	
	Ipso-protonated	N/A	No stable minimum located
	O-protonated	-15.4	
	Cyclohexa-2,4-dien-1-one	131.1	
	Proton-transferred	-12.9	
**3**	Para-protonated	0	
	Meta-protonated	73.8	
	Ortho-protonated	-15	
	Ipso-protonated	N/A	No stable minimum located
	O-protonated	-46.7	
	Cyclohexa-2,4-dien-1-one	115.1	
	Proton-transferred	-54	
**4**	Para-protonated	0	
	Meta-protonated	71.5	
	Ortho-protonated	-33.2	
	Ipso-protonated	N/A	No stable minimum located
	O-protonated	N/A	No stable minimum located
	Cyclohexa-2,4-dien-1-one	109.4	
	Proton-transferred	-79	
**10**	Para-protonated	0	
	Meta-protonated	N/A	No stable minimum located
	Ortho-protonated	5.2	
	Ipso-protonated	N/A	No stable minimum located
	O-protonated	N/A	No stable minimum located
	Cyclohexa-2,4-dien-1-one	115.1	
	Proton-transferred	N/A	No stable minimum located

SI Table S2. A comparison of the energies of different protonated phenol structures with increasing explicit water solvation. Relative energies are calculated from the para-protonated structure at each solvation level. All energies are given in kJ/mol.
