# Supplementary material for: Quantum Chemical Insights into the Dissociation of Phenol: Shedding Light on Impact Ionization Mass Spectrometry for Icy Moon Exploration
Source: ACS Earth Space Chem. 2026 Mar 12;10(4):1033–46. doi: 10.1021/acsearthspacechem.5c00318 (PMC13093666; doi:10.1021/acsearthspacechem.5c00318)
Supplement: Supplementary file 2 [file sp5c00318_si_002.pdf]

Supplementary Information File 2

Quantum Chemical Insights into the Dissociation of Phenol: Shedding light into Impact Ionisation Mass Spectrometry for Icy Moon Exploration

Thomas R. O'Sullivan,[1] Partha P. Bera,[2,3]* Nozair Khawaja,[1,4] Maryse Napoleoni,[1] Bernd Abel,[5,6] Frank Postberg[1]*

[1]Freie Universität Berlin, Institut für Geologische Wissenschaften, Malteserstr. 74-100, 12249 Berlin, Germany.

[2]NASA Ames Research Center, Moffett Field, Mountain View, California, USA 94035.

[3]Bay Area Environmental Research Institute, Moffett Field, Mountain View, California, USA 94035.

[4]Institute for Space Systems, University of Stuttgart, Pfaffenwaldring 29, 70569 Stuttgart, Germany.

[5]Institute of Chemical Technology, University of Leipzig, Linnéstraße 3, 04103 Leipzig, Germany.

[6]J. Heyrovsky Institute of Physical Chemistry, Czech Academy of Sciences, Dolejškova 2155/3, 182 23 Praha, Czech Republic.

*Corresponding authors: tr.osullivan@fu-berlin.de, partha.bera@nasa.gov

Supplementary Information File 2

Quantum Chemical Data

All geometries are given in xyz coordinates. For the protonated structures, Mulliken charge distributions, dipole moments, and rotational constants are also reported.

Para-protonated phenol

Zero-point corrected energy (Eh): -307.66466980

Mode	Frequency (cm^{-1})
6	150.15
7	314.42
8	347.98
9	427.83
10	513.19
11	569.45
12	593.03
13	633.26
14	798.92
15	829.1
16	834.4
17	903.15
18	908.36
19	989.2
20	1011.39
21	1016.06
22	1061.44
23	1162.54
24	1186.55
25	1204.87
26	1210.4
27	1340.28
28	1364.49

29	1402.97
30	1427.82
31	1534.76
32	1547.5
33	1624.73
34	1705.39
35	3010.36
36	3027.94
37	3197.88
38	3208.26
39	3214.01
40	3236.17
41	3750.87

Optimised geometry:

C	-4.2824270000	2.7687220000	0.0226940000
C	-4.3327160000	1.4247600000	0.0060670000
C	-3.1161890000	0.5960950000	0.0029650000
C	-1.8404500000	1.3320770000	0.0190590000
C	-3.0086030000	3.4088700000	0.0377760000
H	-5.1805610000	3.3718130000	0.0249140000
H	-5.2887130000	0.9185210000	-0.0055690000
H	-3.1393690000	-0.0826850000	-0.8613940000
H	-0.9260280000	0.7542000000	0.0171890000
H	-0.8609830000	3.2283350000	0.0477210000
O	-2.8922650000	4.7031350000	0.0542960000
H	-3.7458330000	5.1612980000	0.0551040000
H	-3.1490080000	-0.1031520000	0.8505490000
C	-1.7882240000	2.6753870000	0.0357090000

Mulliken Atomic Charges:

0 C: -0.136423
1 C: -0.032977
2 C: -0.130807
3 C: -0.028841
4 C: 0.303547

5 H:	0.138698
6 H:	0.177425
7 H:	0.175472
8 H:	0.176966
9 H:	0.165387
10 O:	-0.144779
11 H:	0.257767
12 H:	0.175582
13 C:	-0.097015

Sum of atomic charges: 1.0000000

Dipole moment:

	X	Y	Z
Electronic contribution:	0.334823704	3.336720115	0.042362321
Nuclear contribution:	-0.932019267	-3.776494113	-0.050984149
	--		
Total Dipole Moment:	-0.597195563	-0.439773997	-0.008621828
	--		

Magnitude (a.u.): 0.741699430
Magnitude (Debye): 1.885250129

Rotational Constants:

Rotational constants in cm^{-1}:	0.175623	0.087885	0.059181
Rotational constants in MHz:	5265.038214	2634.733520	1774.211539

Ortho-protonated phenol

Zero-point corrected energy (Eh): -307.65760602

Mode	Frequency (cm^{-1})
6	174.61
7	330.28
8	440.02
9	462.55
10	500.28
11	516.61
12	574.61

13	611.6
14	770.1
15	824.96
16	842.11
17	943.4
18	981.89
19	1023.4
20	1045.72
21	1080.81
22	1089.61
23	1160.36
24	1182.15
25	1191.99
26	1209.85
27	1249.47
28	1282.12
29	1386.94
30	1423.46
31	1513.27
32	1555.71
33	1633.89
34	1673.65
35	2782.69
36	2889.89
37	3148.0
38	3160.71
39	3168.28
40	3177.43
41	3734.06

Optimised geometry:

C	-4.2917760000	2.8572470000	-0.0676350000
C	-4.2476780000	1.3765700000	-0.0439550000
C	-3.0820290000	0.7150160000	0.0048920000
C	-1.8567050000	1.4533170000	0.0350610000
C	-1.7912100000	2.8227350000	0.0167510000
C	-2.9817340000	3.5407240000	-0.0345160000
H	-4.8979860000	3.2179550000	0.7748250000
H	-5.1939240000	0.8548360000	-0.0678140000
H	-3.0488880000	-0.3632260000	0.0220510000
H	-0.9282600000	0.8980290000	0.0745860000
H	-0.8520260000	3.3541220000	0.0401780000
O	-2.9095110000	4.8413180000	-0.0529870000
H	-3.7731490000	5.2771760000	-0.0891780000
H	-4.8407450000	3.1921020000	-0.9585790000

Mulliken Atomic Charges:

0 C: -0.193034
1 C: -0.068620
2 C: -0.082211
3 C: 0.031860
4 C: -0.122833
5 C: 0.274140
6 H: 0.187754
7 H: 0.175925
8 H: 0.158484
9 H: 0.165777
10 H: 0.166761
11 O: -0.136966
12 H: 0.255404
13 H: 0.187559
Sum of atomic charges: 1.0000000

Dipole Moment:

	X	Y	Z
Electronic contribution:	1.304908706	1.720128809	0.017138466
Nuclear contribution:	-2.091562264	-1.628695505	-0.044917941
Total Dipole Moment:	-0.786653558	0.091433304	-0.027779475

Magnitude (a.u.): 0.792436476
Magnitude (Debye): 2.014213450

Rotational Constants:

Rotational constants in cm^{-1}:	0.177649	0.086892	0.058970
Rotational constants in MHz:	5325.773038	2604.957161	1767.881698

Meta-protonated phenol

Zero-point corrected energy (Eh): -307.63725443

Mode	Frequency (cm^{-1})
6	176.76
7	191.94
8	315.63
9	390.3
10	401.11
11	504.69
12	513.69
13	601.5
14	675.4
15	814.78
16	816.81
17	917.0
18	956.04
19	994.84
20	1029.4
21	1040.26
22	1047.23
23	1133.62
24	1147.41
25	1200.35
26	1206.49
27	1250.38
28	1314.19

29	1351.1
30	1428.05
31	1471.59
32	1498.79
33	1627.04
34	1670.22
35	2972.46
36	2975.77
37	3176.08
38	3212.35
39	3218.07
40	3234.54
41	3826.09

Optimised geometry:

O	0.3013130000	2.9618260000	-0.0046370000
C	1.3335640000	0.8745040000	-0.0543910000
C	1.2750950000	-0.5799270000	-0.0713260000
C	-0.0371660000	-1.2093160000	-0.0493580000
C	-1.1637010000	-0.4449270000	-0.0141760000
C	-1.0449460000	0.9508860000	0.0006730000
C	0.1889870000	1.6264250000	-0.0188800000
H	1.8481690000	-0.9619740000	-0.9351170000
H	-0.5537900000	3.4046650000	0.0212910000
H	-0.0923960000	-2.2888960000	-0.0616570000
H	-2.1456840000	-0.8926740000	0.0025210000
H	-1.9543110000	1.5420270000	0.0287820000
H	2.2963280000	1.3676730000	-0.0700960000
H	1.8867430000	-0.9832160000	0.7555620000

Mulliken Atomic Charges:

0 O: -0.218405
1 C: -0.038896
2 C: -0.129445
3 C: -0.014189
4 C: -0.088287
5 C: -0.008302
6 C: 0.198489
7 H: 0.191002

8 H: 0.237349
9 H: 0.180679
10 H: 0.162353
11 H: 0.151025
12 H: 0.185582
13 H: 0.191045
Sum of atomic charges: 1.0000000

Dipole Moment:

	X	Y	Z
Electronic contribution:	-0.606014754	2.339980239	0.043047571
Nuclear contribution:	0.328221978	-3.215809589	-0.047186180
Total Dipole Moment:	-0.277792776	-0.875829350	-0.004138609

Magnitude (a.u.): 0.918837855
Magnitude (Debye): 2.335500223

Rotational Constants:

Rotational constants in cm^{-1}:	0.181753	0.084319	0.058171
Rotational constants in MHz:	5448.819043	2527.808349	1743.920734

O-protonated phenol

Zero-point corrected energy (Eh): -307.63725563

Mode	Frequency (cm^{-1})
6	139.96
7	219.66
8	358.55
9	421.76
10	459.55
11	508.66
12	613.09
13	680.63
14	691.89
15	746.87
16	761.71
17	833.52
18	941.27

19	1005.51
20	1014.23
21	1047.57
22	1052.21
23	1059.84
24	1100.13
25	1106.83
26	1190.8
27	1192.82
28	1328.77
29	1342.36
30	1505.31
31	1517.53
32	1637.62
33	1643.53
34	1708.31
35	3206.21
36	3216.41
37	3224.39
38	3230.98
39	3236.89
40	3660.11
41	3741.41

Optimised geometry:

O	-3.4676460000	4.8362030000	-0.3151850000
C	-4.4986790000	2.6033310000	-0.1638920000
C	-4.3493830000	1.2479840000	0.0978210000
C	-3.1075230000	0.7390020000	0.4555310000
C	-1.9975870000	1.5659900000	0.5585650000
C	-3.3632000000	3.3654740000	-0.0441820000
C	-2.1126110000	2.9269380000	0.3035440000

H	-4.0491010000	5.3080250000	0.3061980000
H	-3.7042270000	5.0486190000	-1.2348290000
H	-5.4544310000	3.0238810000	-0.4441730000
H	-5.2062100000	0.5958410000	0.0206010000
H	-1.0373220000	1.1591730000	0.8375800000
H	-1.2728700000	3.6017800000	0.3736210000
H	-3.0047920000	-0.3170290000	0.6566260000

Mulliken Atomic Charges:

0 O: -0.056846
1 C: -0.136692
2 C: -0.067716
3 C: -0.063417
4 C: -0.060904
5 C: 0.155751
6 C: -0.108990
7 H: 0.298389
8 H: 0.298270
9 H: 0.145788
10 H: 0.146563
11 H: 0.148054
12 H: 0.154708
13 H: 0.147043
Sum of atomic charges: 1.0000000

Dipole Moment

	X	Y	Z
Electronic contribution:	-0.021227013	1.862984920	-0.319394307
Nuclear contribution:	-0.671440397	0.367822912	-0.211428046
Total Dipole Moment:	-0.692667409	2.230807832	-0.530822353

Magnitude (a.u.): 2.395425660
Magnitude (Debye): 6.088688152

Rotational constants in cm^{-1}:	0.184529	0.083200	0.057853
Rotational constants in MHz:	5532.047656	2494.277302	1734.380593

Ipso-protonated phenol

Zero-point corrected energy (Eh): -307.61828241

Mode	Frequency (cm^{-1})
6	175.06

7	228.04
8	331.96
9	384.49
10	397.72
11	517.33
12	583.14
13	595.73
14	704.03
15	758.04
16	810.31
17	913.95
18	983.8
19	1026.78
20	1043.72
21	1048.01
22	1082.44
23	1095.26
24	1134.51
25	1187.73
26	1202.92
27	1219.56
28	1280.98
29	1364.75
30	1420.77
31	1490.97
32	1503.08
33	1591.29
34	1664.67
35	2582.81
36	3199.59

37	3205.5
38	3213.66
39	3230.73
40	3234.51
41	3824.27

Optimised geometry:

O	-2.9179040000	4.8462800000	-0.3391290000
C	-3.0289030000	3.5532410000	0.1146170000
C	-1.7875250000	2.7956920000	0.0204020000
C	-1.8081740000	1.4288090000	0.0318490000
C	-3.0379230000	0.7649410000	0.0029970000
C	-4.2662840000	1.4433220000	-0.0212100000
C	-4.2778400000	2.8066650000	-0.0385360000
H	-3.0247370000	3.4914760000	1.2645790000
H	-5.2053130000	3.3640850000	-0.0796320000
H	-3.6569370000	5.3921430000	-0.0491490000
H	-0.8682280000	3.3666770000	0.0200530000
H	-0.8869190000	0.8664750000	0.0280310000
H	-3.0442860000	-0.3173150000	-0.0078480000
H	-5.1894900000	0.8854410000	-0.0638250000

Mulliken Atomic Charges:

0 O: -0.249269
1 C: 0.139476
2 C: -0.011933
3 C: -0.091952
4 C: 0.056809
5 C: -0.095382
6 C: -0.024179
7 H: 0.179310
8 H: 0.169852
9 H: 0.238220
10 H: 0.189623
11 H: 0.165850
12 H: 0.168343
13 H: 0.165233
Sum of atomic charges: 1.0000000

Dipole Moment:

	X	Y	Z
Electronic contribution:	0.216856639	0.602604364	-0.654944490
Nuclear contribution:	-0.678585778	-1.119928474	1.244774335
	--		
Total Dipole Moment:	-0.461729139	-0.517324110	0.589829845

Magnitude (a.u.): 0.910339101
Magnitude (Debye): 2.313898106

Rotational Constants:

Rotational constants in cm^{-1}:	0.175758	0.087077	0.059460
Rotational constants in MHz:	5269.101435	2610.495464	1782.558914

Protonated cyclohexa-2,4-dien-1-one

Zero-point corrected energy (Eh): -307.61973704

Mode	Frequency (cm^{-1})
6	108.94
7	196.96
8	333.84
9	374.52
10	455.46
11	523.94
12	549.39
13	654.35
14	711.48
15	747.93
16	937.96
17	951.71
18	975.03
19	990.12
20	1014.16
21	1058.34
22	1071.96
23	1155.34
24	1177.02
25	1181.4
26	1211.82

27	1323.99
28	1339.87
29	1392.13
30	1407.19
31	1441.3
32	1569.93
33	1580.05
34	1919.51
35	2977.44
36	2984.84
37	3134.36
38	3134.97
39	3184.14
40	3186.4
41	3230.45

Optimised geometry:

O	-1.7644540000	4.1351770000	-2.1885340000
C	-3.6072820000	3.3088400000	-0.8979540000
C	-3.8715570000	1.9528760000	-0.4184230000
C	-2.9206120000	1.1529920000	0.1813640000
C	-1.6070730000	1.5735420000	0.2102730000
C	-2.1285430000	3.5268760000	-1.2417650000
C	-1.1687840000	2.8996120000	-0.2233130000
H	-3.8013320000	3.9992940000	-0.0602260000
H	-4.2609190000	3.6078740000	-1.7144680000
H	-1.2186780000	3.5673240000	0.6529850000
H	-4.8629390000	1.5445330000	-0.5823340000
H	-0.8461350000	0.8720210000	0.5347250000
H	-0.1393350000	2.9149990000	-0.5747560000
H	-3.1803820000	0.1613670000	0.5199500000

Mulliken Atomic Charges:

0 O: -0.123070
1 C: -0.205788
2 C: 0.059907

3 C:	-0.100342
4 C:	0.059986
5 C:	0.198284
6 C:	-0.205028
7 H:	0.194131
8 H:	0.177107
9 H:	0.194133
10 H:	0.199350
11 H:	0.198948
12 H:	0.177064
13 H:	0.175317

Sum of atomic charges: 1.0000000

Dipole Moment:

	X	Y	Z
Electronic contribution:	0.646863335	0.836016149	-1.838389527
Nuclear contribution:	-1.254070061	-2.022696293	3.313654301
Total Dipole Moment:	-0.607206725	-1.186680144	1.475264774

Magnitude (a.u.): 1.988294728

Magnitude (Debye): 5.053843563

Rotational Constants:

Rotational constants in cm^{-1}:	0.158901	0.090902	0.062287
Rotational constants in MHz:	4763.725959	2725.185133	1867.331557

C_2H_2O (neutral, doublet)

Zero-point corrected energy (Eh): -152.5157557

Mode	Frequency (cm^{-1})
6	410.75
7	460.88
8	567.87
9	671.76
10	1093.82
11	1267.5
12	2304.43

Optimised geometry:

O	2.729558	-0.257300	0.284518

C	1.486841	-0.030640	-0.067396
C	0.361600	0.234740	-0.369793
H	2.965137	-1.164963	0.063032
H	-0.637380	0.466579	-0.634917

C_2H_2O (cation, doublet)

Zero-point corrected energy (Eh): -152.15841176

Mode	Frequency (cm^{-1})
8	470.33
9	513.68
10	639.44
11	1057.03
12	1313.29
13	3409.61
14	3723.25

Optimised geometry:

C	-5.1341020000	2.1787820000	0.0000620000
C	-3.9994310000	2.6696280000	0.0002630000
H	-6.1074600000	1.7283830000	-0.0002160000
O	-2.9158040000	3.2710210000	0.0005090000
H	-2.1167430000	2.6988560000	-0.0006190000

C_2H_2 (neutral, singlet)

Zero-point corrected energy (Eh): -77.29535876

Mode	Frequency (cm^{-1})
5	686.15
6	686.23
7	770.51
8	770.61
9	2092.82
10	3426.53

11	3533.87

Optimised geometry:

H	-0.0000000000	-0.0000000000	-1.6587770000
C	0.0000000000	-0.0000000000	-0.5973470000
C	0.0000000000	0.0000000000	0.5973480000
H	-0.0000000000	-0.0000000000	1.6587770000

C_2H_2 (cation, doublet)

Zero-point corrected energy (Eh): -76.82101453

Mode	Frequency (cm^{-1})
6	488.28
7	567.8
8	1205.39
9	1437.93
10	3005.78
11	3102.98

Optimised geometry:

C	-0.1845540000	2.1720240000	-2.4047270000
C	-0.4696910000	0.9836900000	-1.7670190000
H	-0.6250750000	2.3889120000	-3.3872010000
H	0.4854000000	2.9036340000	-1.9333430000

C_2H_3O (anion, singlet)

Zero-point corrected energy (Eh): -153.11278017

Mode	Frequency (cm^{-1})
6	549.04
7	550.42
8	640.2
9	736.05

10	908.43
11	1021.34
12	1266.89
13	1375.75
14	1538.0
15	2843.28
16	3136.31
17	3495.83

Optimised geometry:

O	0.8995670000	1.3556690000	0.2148940000
C	3.0399660000	0.5441230000	0.4907310000
C	2.2568840000	1.6474590000	0.4707570000
H	2.6308250000	-0.4735680000	0.3203930000
H	4.1078420000	0.6010030000	0.6746360000
H	0.8205740000	0.3876990000	0.1040670000

C_2H_3O (neutral, doublet)

Zero-point corrected energy (Eh): -153.08278707

Mode	Frequency (cm^{-1})
6	340.36
7	447.39
8	658.64
9	838.79
10	965.01
11	1146.66
12	1242.89
13	1402.69
14	1740.09
15	3122.2
16	3249.15

17	3824.53

Optimised geometry:

C	-0.2804910000	2.1138220000	0.3471160000
C	-0.1443670000	0.8371010000	0.0782310000
H	0.5615160000	0.2321770000	0.6241270000
H	-0.7293880000	0.3775810000	-0.7126230000
O	-1.1773580000	2.9999400000	-0.0824360000
H	-0.7414630000	3.8409890000	-0.2544140000

C_2H_3O (cation, singlet)

Zero-point corrected energy (Eh): -152.81805578

Mode	Frequency (cm^{-1})
6	378.63
7	396.61
8	812.38
9	849.35
10	893.76
11	1131.03
12	1207.11
13	1345.63
14	2137.07
15	3115.21
16	3207.6
17	3546.53

Optimised geometry:

O	2.8026080000	-0.3518720000	-0.2689980000
C	1.5921940000	-0.5022480000	-0.1606970000
C	0.3317750000	-0.6585150000	-0.1665190000
H	3.3034650000	-0.2920630000	0.5756040000
H	-0.0851290000	-1.6623530000	-0.1624270000
H	-0.3175620000	0.2131690000	-0.1568730000

C_2H_3 (neutral, doublet)

Zero-point corrected energy (Eh): -77.77227366

Mode	Frequency (cm^{-1})
6	488.09
7	885.04
8	1111.41
9	1306.45
10	1308.94
11	1434.6
12	2896.14
13	3004.35
14	3041.56

Optimised geometry:

C	-1.5239470000	-1.3865300000	0.0373990000
C	-2.6755590000	-1.9559650000	0.6718110000
H	-1.2089620000	-0.3897220000	0.3661210000
H	-0.8221780000	-2.1167330000	0.5039960000
H	-1.4341360000	-1.5095810000	-1.0478020000

C_2H_3 (cation, singlet)

Zero-point corrected energy (Eh): -77.46758953

Mode	Frequency (cm^{-1})
6	278.24
7	287.58
8	1071.94
9	1195.72
10	1196.77
11	1503.34

12	2862.71
13	2929.05
14	2929.71

Optimised geometry:

C	-1.4740230000	-1.4526380000	0.0818450000
C	-2.6712030000	-1.8399390000	0.5791250000
H	-1.2975260000	-0.4002090000	0.4010790000
H	-0.6967890000	-2.1336530000	0.4974620000
H	-1.5252410000	-1.5320920000	-1.0279840000

C_3H_2O (neutral, singlet)

Zero-point corrected energy (Eh): -190.54522030

Mode	Frequency (cm^{-1})
6	168.21
7	192.67
8	606.62
9	717.63
10	1014.93
11	1058.21
12	1254.86
13	1362.07
14	1515.92
15	2102.78
16	3186.78
17	3746.47

Optimised geometry:

O	2.3324760000	-0.1138740000	-0.0245270000
C	1.0588390000	0.1819660000	-0.1915070000
C	0.0498940000	-0.6825680000	-0.0469660000
C	-0.8982640000	-1.5103560000	0.0481520000

H	2.4028440000	-1.0498420000	0.2155480000
H	0.9230260000	1.2225830000	-0.4628830000

C_3H_3O (anion, singlet)

Zero-point corrected energy (Eh): -191.12318376

Mode	Frequency (cm^{-1})
6	347.37
7	487.71
8	507.63
9	607.38
10	723.73
11	744.8
12	907.33
13	1067.13
14	1129.83
15	1237.66
16	1385.84
17	1482.82
18	2939.98
19	2993.56
20	3745.48

Optimised geometry:

O	-0.8675610000	-1.5359970000	-0.6385020000
C	-0.9424610000	0.7269180000	0.1951930000
C	1.0936330000	-0.4343620000	0.1703570000
C	-0.2112850000	-0.3564100000	-0.2709270000
H	-0.1633970000	-2.1900450000	-0.5372470000
H	-0.2992140000	1.6124700000	0.0583120000
H	1.5455720000	0.5657300000	0.1020090000

C_3H_3O (neutral, doublet)

Zero-point corrected energy (Eh): -191.13134526

Mode	Frequency (cm^{-1})
6	351.87
7	439.5
8	505.96
9	599.63
10	727.2
11	879.94
12	941.44
13	977.5
14	1112.82
15	1177.0
16	1393.37
17	1823.51
18	3097.76
19	3323.89
20	3807.45

Optimised geometry:

C	-0.6268490000	2.9123620000	0.3635690000
C	0.2582690000	3.9382170000	-0.0089730000
C	0.8595080000	2.8016520000	0.2805580000
H	1.7332260000	2.2096410000	0.4609250000
H	-1.4048270000	2.4037460000	-0.1988440000
O	0.3218950000	5.2021500000	-0.3947810000
H	1.2478670000	5.4600220000	-0.4690640000

C_3H_3O (cation, singlet)

Zero-point corrected energy (Eh): -190.83127246

Mode	Frequency (cm^{-1})

6	192.14
7	204.28
8	506.15
9	671.16
10	812.17
11	1003.77
12	1074.73
13	1130.44
14	1278.84
15	1448.99
16	1591.67
17	1669.12
18	3132.76
19	3186.61
20	3677.2

Optimised geometry:

O	2.3336240000	-0.0186720000	-0.0847840000
C	1.0672770000	-0.0206230000	-0.1805680000
C	0.2323840000	-1.1500550000	-0.0323180000
C	-1.0598450000	-0.8868230000	-0.1795950000
H	2.7414410000	-0.8824820000	0.1082030000
H	0.5755740000	-2.1615280000	0.1833190000
H	0.6540660000	0.9601390000	-0.3959350000

C_3H_3 (neutral, quartet)

Zero-point corrected energy (Eh): -115.82982315

Mode	Frequency (cm^{-1})
6	463.08
7	547.18
8	553.52

9	772.97
10	822.07
11	883.03
12	1177.08
13	1268.97
14	1388.26
15	2963.45
16	3268.61
17	3276.01

Optimised geometry:

C	-1.3672000000	0.0177440000	0.0942870000
C	-0.7070490000	1.2043760000	0.0246450000
C	0.6413920000	1.3640190000	-0.0526180000
H	-2.3924470000	-0.3013010000	0.1587480000
H	-1.3187400000	2.1178410000	0.0320620000
H	1.3256570000	2.1917980000	-0.1133230000

C_3H_3 (cation, singlet)

Zero-point corrected energy (Eh): -115.62816385

Mode	Frequency (cm^{-1})
6	248.79
7	321.14
8	652.77
9	924.53
10	1028.27
11	1139.06
12	1159.77
13	1471.24
14	2146.85
15	3119.93

16	3225.68
17	3366.97

Optimised geometry:

C	-1.5821770000	-0.0262970000	0.0946280000
C	-0.7901370000	0.9016150000	0.0383490000
C	0.0788330000	1.9196150000	-0.0237670000
H	-2.2777370000	-0.8414300000	0.1433610000
H	-0.2740160000	2.9468970000	-0.0034870000
H	1.1460290000	1.7293590000	-0.0954380000

C_3H_4O (neutral, singlet)

Zero-point corrected energy (Eh): -191.73592356

Mode	Frequency (cm^{-1})
6	99.77
7	331.75
8	580.06
9	584.96
10	738.47
11	930.81
12	1044.34
13	1049.86
14	1168.08
15	1243.8
16	1309.78
17	1360.82
18	1413.98
19	1599.41
20	2971.69
21	3034.59
22	3223.3

23	3774.52

Optimised geometry:

O	2.3416980000	-0.0285690000	-0.0849420000
C	1.0269460000	-0.0716740000	-0.1738980000
C	0.2329780000	-1.1743310000	-0.0240790000
C	-1.1388150000	-0.9231970000	-0.1718630000
H	2.6829440000	-0.9121680000	0.1072860000
H	0.7213470000	-2.1300100000	0.1924980000
H	-1.6837150000	-1.8720600000	-0.0321310000
H	0.5761750000	0.8879870000	-0.3884900000

C_3H_4O (cation, doublet)

Zero-point corrected energy (Eh): -191.47069464

Mode	Frequency (cm^{-1})
6	166.0
7	315.28
8	558.78
9	647.15
10	769.24
11	819.88
12	923.47
13	1082.6
14	1140.44
15	1242.1
16	1286.23
17	1436.05
18	1585.83
19	1632.06
20	3107.96
21	3195.12

22	3259.18
23	3675.77

Optimised geometry:

O	2.3187490000	0.0065500000	-0.0928650000
C	1.0519800000	-0.0546670000	-0.1731290000
C	0.2749640000	-1.2290990000	-0.0114960000
C	-1.0402940000	-1.1119010000	-0.1282880000
H	2.7549160000	-0.8441770000	0.0963250000
H	0.7556860000	-2.1838360000	0.2004270000
H	-1.9393840000	-1.7080190000	-0.0827990000
H	0.5829430000	0.9011290000	-0.3837970000

C_3H_4 (neutral, singlet)

Zero-point corrected energy (Eh): -116.48390258

Mode	Frequency (cm^{-1})
6	145.31
7	611.69
8	771.78
9	942.96
10	964.42
11	1016.05
12	1093.3
13	1173.02
14	1325.36
15	1426.36
16	1598.89
17	2979.63
18	3114.23
19	3148.14
20	3255.2

Optimised geometry:

C	-1.5192110000	0.3652880000	-0.2563310000
C	-0.6391410000	1.1865110000	0.3636620000
C	0.5434400000	1.2418430000	-0.3940980000
H	-2.5909620000	0.4677880000	-0.1335560000
H	-0.9604340000	1.8837350000	1.1339330000
H	0.8860640000	2.2898740000	-0.4316540000
H	-1.1448520000	-0.3587870000	-0.9650300000

C_3H_4 (cation, doublet)

Zero-point corrected energy (Eh): -116.23507543

Mode	Frequency (cm^{-1})
6	319.78
7	333.92
8	781.0
9	804.78
10	922.22
11	940.49
12	993.41
13	1074.86
14	1344.7
15	1397.78
16	1586.37
17	3082.55
18	3101.81
19	3185.31
20	3187.77

Optimised geometry:

C	-0.0000000000	-0.0000000000	1.3101410000
H	-0.3883770000	0.8521400000	1.8649070000
H	0.3883750000	-0.8521400000	1.8649090000

C	0.0000010000	-0.0000000000	-0.0000020000
C	0.0000020000	-0.0000010000	-1.3101440000
H	0.3883760000	0.8521440000	-1.8649050000
H	-0.3883770000	-0.8521420000	-1.8649060000

C_4H_3 (neutral, doublet)

Zero-point corrected energy (Eh): -153.91711349

Mode	Frequency (cm^{-1})
7	221.15
8	323.13
9	495.66
10	654.52
11	748.05
12	802.55
13	844.51
14	971.63
15	1262.34
16	1679.52
17	2014.47
18	2433.13
19	3100.15
20	3283.87

Optimised geometry:

C	-1.5923250000	0.0263360000	0.0933370000
C	-0.8014350000	1.0654540000	0.0362220000
C	0.6359580000	0.9687110000	-0.0624070000
C	1.8728120000	0.7537670000	-0.1466230000
H	-2.6481420000	-0.1635150000	0.1667980000
H	-1.2161110000	2.0716850000	0.0624430000
H	1.3878600000	1.9568060000	-0.1225480000

C_4H_3 (cation, singlet)

Zero-point corrected energy (Eh): -153.66617910

Mode	Frequency (cm^{-1})
6	221.56
7	373.32
8	494.29
9	700.83
10	841.53
11	891.56
12	978.03
13	995.48
14	1031.01
15	1328.57
16	1466.65
17	1760.73
18	3119.6
19	3223.62
20	3265.6

Optimised geometry:

C	-1.5038230000	0.6813080000	-0.1141000000
C	-0.1820490000	0.7080830000	-0.1577010000
C	1.0124340000	1.3491520000	0.2126550000
C	1.1770560000	0.2548130000	-0.5221890000
H	-2.0139000000	0.1525890000	0.6863850000
H	-2.0864510000	1.1912630000	-0.8763230000
H	1.2372020000	2.2336410000	0.7897780000

C_4H_4O (neutral, singlet)

Zero-point corrected energy (Eh): -229.89041450

Mode	Frequency (cm^{-1})
6	172.57
7	190.31
8	440.05
9	455.39
10	507.02
11	550.84
12	627.35
13	713.69
14	830.48
15	986.74
16	1032.89
17	1156.51
18	1265.21
19	1337.33
20	1393.25
21	1722.92
22	2244.03
23	3161.24
24	3221.39
25	3495.91
26	3820.51

Optimised geometry:

O	2.6220050000	0.0052910000	-0.0648190000
C	1.2795680000	-0.0353560000	-0.1953250000
C	0.4930960000	-1.0716220000	0.0981650000
C	-0.9201520000	-1.0184220000	-0.0750340000
C	-2.1113510000	-0.9937630000	-0.2167360000
H	2.9368830000	-0.8374440000	0.2784480000
H	0.9213320000	-1.9913460000	0.4808460000

H -3.1646120000 -0.9691000000 -0.3396540000
H 0.8858720000 0.8952510000 -0.5783120000

C_4H_4O (cation, doublet)

Zero-point corrected energy (Eh): -229.50409254

Mode	Frequency (cm^{-1})
6	137.87
7	155.62
8	283.58
9	327.19
10	557.64
11	632.68
12	718.62
13	912.79
14	962.03
15	1029.13
16	1087.55
17	1176.69
18	1315.97
19	1413.88
20	1424.5
21	1597.75
22	1664.92
23	3017.85
24	3189.59
25	3198.82
26	3698.26

Optimised geometry:

O 2.3616290000 -0.0445570000 -0.1002960000

C	1.0775490000	-0.0148970000	-0.1416650000
C	0.2493550000	-1.1328020000	-0.0059640000
C	-1.1456060000	-1.0128280000	-0.0631270000
C	-1.9517580000	0.0356760000	-0.2357650000
H	2.7409390000	-0.9297250000	0.0372840000
H	0.6900870000	-2.1098160000	0.1463370000
H	-1.7978800000	-1.8949210000	0.0442880000
H	0.6836760000	0.9842890000	-0.2958410000

C_4H_4 (neutral, singlet)

Zero-point corrected energy (Eh): -154.60108399

Mode	Frequency (cm^{-1})
6	542.02
7	596.92
8	614.4
9	738.08
10	858.39
11	908.54
12	909.85
13	968.44
14	1062.74
15	1129.34
16	1194.5
17	1273.03
18	1658.19
19	1662.11
20	3221.47
21	3238.56
22	3257.84
23	3268.59

Optimised geometry:

C	-0.7675460000	0.1121340000	0.0641850000
C	-0.7826430000	1.4377870000	0.0236560000
C	0.7849700000	1.4529400000	-0.0647380000
C	0.8000520000	0.1272710000	-0.0247090000
H	-1.5176810000	-0.6588530000	0.1305360000
H	-1.5501010000	2.1941890000	0.0433070000
H	1.5351260000	2.2239260000	-0.1308650000
H	1.5675240000	-0.6291110000	-0.0445770000

C_4H_4 (cation, doublet)

Zero-point corrected energy (Eh): -154.24393905

Mode	Frequency (cm^{-1})
6	165.14
7	211.04
8	216.97
9	486.24
10	649.3
11	665.88
12	741.11
13	753.65
14	782.94
15	786.96
16	992.82
17	1070.66
18	1792.62
19	1813.61
20	3230.77
21	3232.2
22	3363.26
23	3375.64

Optimised geometry:

C	-1.5527150000	0.3294680000	0.2288570000
C	-0.8519480000	1.2099210000	-0.3111220000
C	0.7393100000	1.1413960000	0.2115760000
C	1.4412740000	0.4302360000	-0.5364290000
H	-2.0323000000	-0.4690230000	0.7588660000
H	-1.0102530000	2.0358190000	-0.9885740000
H	0.8975040000	1.7661340000	1.0780660000
H	1.9185040000	-0.2138940000	-1.2476730000

C_4H_5O (neutral, doublet)

Zero-point corrected energy (Eh): -230.43407330

Mode	Frequency (cm^{-1})
7	205.13
8	261.75
9	429.74
10	465.47
11	593.38
12	620.36
13	812.9
14	856.94
15	866.97
16	984.64
17	1012.21
18	1157.85
19	1263.03
20	1283.07
21	1341.28
22	1400.11
23	1677.02
24	1731.9
25	3053.71

26	3160.77
27	3206.8
28	3273.01
29	3820.51

Optimised geometry:

O	2.4137650000	-0.0377280000	-0.1168390000
C	1.0594320000	-0.0339350000	-0.1292420000
C	0.2686020000	-1.0975390000	-0.0038050000
C	-1.1987770000	-1.0339960000	-0.0243180000
C	-1.9604250000	0.0215870000	-0.1622310000
H	2.7239710000	-0.9420560000	-0.0020670000
H	0.7161240000	-2.0771030000	0.1223700000
H	-1.7038150000	-1.9957770000	0.0912770000
H	-3.0125380000	0.2418030000	-0.1983440000
H	0.6676710000	0.9660120000	-0.2570720000

C_4H_5O (cation, singlet)

Zero-point corrected energy (Eh): -230.21560737

Mode	Frequency (cm^{-1})
6	142.82
7	179.5
8	403.15
9	447.48
10	535.86
11	591.5
12	739.18
13	889.15
14	910.01
15	929.69
16	1075.37
17	1090.12

18	1147.68
19	1299.56
20	1323.14
21	1401.84
22	1431.38
23	1613.69
24	1998.99
25	3135.25
26	3192.47
27	3196.57
28	3227.3
29	3719.02

Optimised geometry:

O	2.3850450000	0.0665060000	-0.0955990000
C	1.1298590000	-0.1263410000	-0.2506670000
C	0.3881670000	-1.2389360000	0.1613270000
C	-0.9282910000	-1.0302180000	0.0295810000
C	-2.1261760000	-0.6294200000	-0.1802420000
H	2.8601110000	-0.6848080000	0.2957910000
H	0.8250030000	-2.0984920000	0.6539920000
H	-2.7281240000	-1.0401270000	-0.9824500000
H	-2.5150610000	0.1940140000	0.4095520000
H	0.6156410000	0.7251180000	-0.6835580000

C_4H_5 (neutral, doublet)

Zero-point corrected energy (Eh): -155.20037502

Mode	Frequency (cm^{-1})
6	134.73
7	268.53
8	500.3
9	597.55

10	720.8
11	799.19
12	870.15
13	968.32
14	972.74
15	1034.45
16	1079.9
17	1267.84
18	1332.3
19	1441.78
20	1681.2
21	1717.25
22	3057.63
23	3154.96
24	3173.15
25	3246.79
26	3268.09

Optimised geometry:

C	-1.5201570000	0.0351750000	-0.1242560000
C	-0.8401490000	1.1545210000	0.0871980000
C	0.6102880000	1.3100830000	-0.1325650000
C	1.5004990000	0.3531130000	-0.1095700000
H	-2.5819100000	-0.0182920000	0.0678510000
H	-1.3619260000	2.0387190000	0.4334680000
H	0.9598880000	2.3279360000	-0.3190510000
H	2.5644980000	0.2482250000	-0.2326280000
H	-1.0317900000	-0.8536970000	-0.5007770000

C_4H_5 (cation, singlet)

Zero-point corrected energy (Eh): -154.93199923

Mode	Frequency (cm^{-1})
7	179.8
8	282.33
9	397.51
10	646.44
11	806.4
12	819.77
13	892.82
14	958.48
15	1050.49
16	1072.3
17	1254.85
18	1285.35
19	1444.27
20	1617.25
21	1919.74
22	3075.26
23	3165.63
24	3174.9
25	3180.33
26	3274.39

Optimised geometry:

C	-1.7173650000	0.0391220000	0.1912410000
C	-0.9999670000	1.1712290000	0.0977400000
C	0.3759000000	1.0147860000	0.0308040000
C	1.6041830000	0.7262770000	-0.0810070000
H	-2.7753550000	0.0875840000	0.4013550000
H	-1.4203580000	2.1622930000	0.2305980000
H	2.3557030000	1.3848140000	-0.5088480000
H	1.8692300000	-0.3105060000	0.1454220000
H	-1.2513090000	-0.9369910000	0.1805330000

C_5H_4O (neutral, singlet)

Zero-point corrected energy (Eh): -267.89915473

Mode	Frequency (cm^{-1})
6	121.56
7	141.55
8	245.66
9	289.07
10	295.98
11	460.38
12	587.25
13	715.85
14	834.12
15	971.53
16	986.54
17	1039.23
18	1156.01
19	1207.67
20	1323.22
21	1340.64
22	1407.7
23	1479.6
24	1648.41
25	2069.11
26	3135.18
27	3185.86
28	3207.29
29	3794.16

Optimised geometry:

O	2.5332370000	-0.2161910000	-0.0880370000
C	1.2092420000	-0.1214850000	-0.1001010000
C	0.3373070000	-1.1465250000	0.0005260000
C	-1.0707600000	-0.9083400000	-0.0261130000
C	-1.6633110000	0.2913520000	-0.1480250000
C	-2.2110660000	1.4241570000	-0.2632690000
H	2.8030820000	-1.1381400000	0.0037850000
H	0.6950770000	-2.1631260000	0.1031090000
H	-1.7048290000	-1.7867060000	0.0612350000
H	0.8641910000	0.8996490000	-0.2027660000

C_5H_4O (cation, doublet)

Zero-point corrected energy (Eh): -267.57817921

Mode	Frequency (cm^{-1})
6	124.39
7	159.61
8	221.45
9	281.48
10	391.48
11	453.87
12	694.76
13	729.11
14	860.87
15	967.82
16	1026.64
17	1080.88
18	1149.75
19	1216.55
20	1296.51
21	1411.89
22	1445.34

23	1582.99
24	1613.75
25	2232.25
26	3180.36
27	3194.29
28	3209.97
29	3697.02

Optimised geometry:

O	2.4957510000	-0.1837690000	-0.0867350000
C	1.2200800000	-0.0904530000	-0.0901200000
C	0.3254210000	-1.1641700000	-0.0200450000
C	-1.0197990000	-0.9316810000	-0.0326180000
C	-1.6296470000	0.3299100000	-0.1124960000
C	-2.1975530000	1.3909390000	-0.1805150000
H	2.8274600000	-1.0965600000	-0.0296600000
H	0.6966260000	-2.1779830000	0.0442150000
H	-1.6935120000	-1.7801370000	0.0233290000
H	0.8691090000	0.9344000000	-0.1547340000

C_5H_5O (neutral, doublet)

Zero-point corrected energy (Eh): -268.60414743

Mode	Frequency (cm^{-1})
6	261.86
7	408.81
8	451.05
9	550.27
10	638.63
11	661.66
12	687.18
13	718.07
14	735.61

15	864.82
16	905.67
17	937.65
18	1032.1
19	1049.25
20	1082.94
21	1129.39
22	1293.26
23	1334.44
24	1364.17
25	1457.42
26	1578.48
27	1579.98
28	3227.12
29	3237.13
30	3250.48
31	3263.65
32	3833.36

Optimised geometry:

C	-0.9321330000	3.5877300000	-0.0023300000
C	-2.0726210000	2.7253860000	-0.0061030000
C	-1.5880550000	1.4509650000	-0.0173330000
C	0.2644560000	2.8130420000	-0.0114900000
C	-0.1301250000	1.5029690000	-0.0207270000
H	0.5188570000	0.6423520000	-0.0291620000
O	-1.0349470000	4.9212700000	0.0083580000
H	-0.1530630000	5.3070540000	0.0090920000
H	1.2708730000	3.1999410000	-0.0110860000
H	-3.0976140000	3.0535220000	-0.0008500000
H	-2.1719980000	0.5451600000	-0.0229000000

C_5H_5O (cation, singlet)

Zero-point corrected energy (Eh): -268.33185054

Mode	Frequency (cm^{-1})
6	184.11
7	402.13
8	428.56
9	657.99
10	660.79
11	666.01
12	727.93
13	798.37
14	850.47
15	891.47
16	958.22
17	994.3
18	1017.15
19	1098.99
20	1112.65
21	1123.87
22	1303.19
23	1352.02
24	1360.35
25	1613.88
26	1674.12
27	1678.12
28	3245.82
29	3254.73
30	3266.65
31	3280.41
32	3683.62

Optimised geometry:

C	-2.2132180000	2.9120410000	0.2738900000
C	-3.4653940000	2.1537140000	0.2894970000
C	-3.0922100000	0.8765730000	0.3690890000
H	-4.4560410000	2.5743930000	0.2446210000
C	-1.5676310000	0.7895690000	0.4066320000
H	-3.7412820000	0.0155700000	0.4034420000
C	-1.0554960000	2.0162820000	0.3489150000
H	-1.0254340000	-0.1402950000	0.4704190000
H	-0.0306640000	2.3443300000	0.3525730000
O	-2.0738690000	4.1701870000	0.2063720000
H	-2.9143910000	4.6602770000	0.1606500000

C_5H_5 (neutral, doublet)

Zero-point corrected energy (Eh): -193.36180296

Mode	Frequency (cm^{-1})
6	103.45
7	493.02
8	527.22
9	687.03
10	735.34
11	836.38
12	846.08
13	908.41
14	928.43
15	930.16
16	971.89
17	1044.28
18	1086.75
19	1154.54
20	1214.1
21	1297.8

22	1405.01
23	1474.25
24	1592.43
25	3223.08
26	3225.01
27	3240.55
28	3255.39
29	3263.53

Optimised geometry:

C	-0.8480600000	-0.1954240000	0.0122270000
C	-0.6886400000	1.2631770000	-0.0520200000
C	0.6321190000	1.5372970000	0.0252380000
C	1.3372090000	0.2673310000	0.1399160000
C	0.4019040000	-0.7739080000	0.1286650000
H	-1.7926380000	-0.7153540000	-0.0264240000
H	-1.4971250000	1.9695600000	-0.1446080000
H	1.0998710000	2.5074720000	0.0073960000
H	0.6189620000	-1.8259900000	0.1978170000
H	2.4078130000	0.1577030000	0.2197410000

C_5H_5 (cation, singlet)

Zero-point corrected energy (Eh): -193.04747078

Mode	Frequency (cm^{-1})
6	235.91
7	336.07
8	369.29
9	704.34
10	809.06
11	818.32
12	867.98
13	971.97

14	982.06
15	1019.09
16	1060.36
17	1081.88
18	1113.3
19	1153.04
20	1274.02
21	1303.51
22	1359.44
23	1608.62
24	1644.93
25	3202.34
26	3244.5
27	3250.77
28	3277.83
29	3282.58

Optimised geometry:

C	0.1566460000	-0.4173340000	-0.0071800000
C	-0.9634090000	0.4907600000	-0.0530750000
C	1.4166620000	0.2789690000	0.0875350000
C	1.1147360000	1.5824300000	0.1018900000
C	-0.4405350000	1.7208240000	0.0098940000
H	-0.9592560000	2.6674880000	0.0017880000
H	-1.9975390000	0.2030340000	-0.1237120000
H	0.0624620000	-1.4975690000	-0.0399740000
H	2.3850580000	-0.1869910000	0.1353180000
H	1.7893160000	2.4228690000	0.1644860000

C_5H_5 (anion, singlet)

Zero-point corrected energy (Eh): -193.42890158

Mode	Frequency (cm^{-1})

6	630.64
7	631.16
8	667.24
9	667.52
10	669.47
11	824.3
12	824.84
13	849.27
14	849.72
15	1015.32
16	1020.27
17	1049.34
18	1050.57
19	1165.56
20	1268.2
21	1391.85
22	1392.05
23	1471.06
24	1471.32
25	3140.44
26	3141.64
27	3169.0
28	3170.79
29	3188.72

Optimised geometry:

C	-0.8737050000	-0.1728710000	0.0092470000
C	-0.7134850000	1.2253470000	-0.0516140000
C	0.6638680000	1.5095430000	0.0289260000
C	1.3545980000	0.2868880000	0.1401380000
C	0.4044240000	-0.7529810000	0.1279640000
H	-1.8147230000	-0.7074790000	-0.0284620000

H	-1.5096670000	1.9533820000	-0.1448560000
H	1.1130190000	2.4946930000	0.0091120000
H	0.6191500000	-1.8120970000	0.1971790000
H	2.4279370000	0.1674390000	0.2203130000

C_5H_6O (neutral, singlet)

Zero-point corrected energy (Eh): -269.21693356

Mode	Frequency (cm^{-1})
6	250.62
7	368.03
8	400.59
9	435.58
10	524.15
11	635.77
12	687.48
13	816.4
14	824.36
15	916.36
16	924.9
17	965.73
18	968.66
19	1016.27
20	1123.46
21	1134.14
22	1170.62
23	1251.18
24	1287.34
25	1369.58
26	1414.83
27	1460.78

28	1622.66
29	1692.57
30	3057.07
31	3093.39
32	3211.62
33	3224.3
34	3247.42
35	3839.78

Optimised geometry:

O	1.9450730000	0.3437960000	0.0520060000
C	0.6632130000	-0.0912500000	-0.0045220000
C	0.1546580000	-1.3279260000	0.1079520000
C	-1.3029040000	-1.2215650000	-0.0323490000
C	-1.6583460000	0.0541440000	-0.2249720000
C	-0.4245180000	0.9115490000	-0.2258240000
H	2.5227720000	-0.4108860000	0.1988780000
H	0.7065690000	-2.2410340000	0.2720330000
H	-1.9761270000	-2.0642580000	0.0159180000
H	-2.6573970000	0.4347140000	-0.3603210000
H	-0.4300650000	1.6619260000	0.5685630000
H	-0.2788560000	1.4443300000	-1.1686640000

C_5H_6O (cation, doublet)

Zero-point corrected energy (Eh): -268.84655313

Mode	Frequency (cm^{-1})
6	111.79
7	159.22
8	252.51
9	305.22
10	367.48
11	468.49

12	513.16
13	606.24
14	757.22
15	827.05
16	949.05
17	1003.32
18	1030.84
19	1047.61
20	1054.9
21	1096.17
22	1166.86
23	1237.05
24	1311.72
25	1404.18
26	1463.43
27	1567.65
28	1635.35
29	1682.39
30	3148.63
31	3161.52
32	3184.89
33	3195.9
34	3259.89
35	3551.44

Optimised geometry:

O	2.3640930000	-0.1928930000	0.4888240000
C	1.1228440000	-0.3168330000	0.3654140000
C	0.2558350000	-1.3075980000	-0.0565750000
C	-1.0932700000	-1.0588030000	-0.0738420000
C	-1.6772230000	0.2409030000	0.0636720000
C	-1.1001520000	1.3447510000	-0.4339140000

H	2.9229230000	-0.9319480000	0.1684100000
H	0.6564620000	-2.2891060000	-0.2933840000
H	-1.7606380000	-1.9056070000	-0.1892620000
H	-2.6574730000	0.2975110000	0.5196970000
H	-1.5510100000	2.3178720000	-0.3011220000
H	-0.2183190000	1.2952920000	-1.0592190000

C_5H_6 (neutral, singlet)

Zero-point corrected energy (Eh): -193.98349228

Mode	Frequency (cm^{-1})
6	344.51
7	526.12
8	682.43
9	726.95
10	816.42
11	818.15
12	916.72
13	939.41
14	971.07
15	976.42
16	978.48
17	1019.42
18	1114.39
19	1130.29
20	1135.05
21	1272.57
22	1327.41
23	1399.03
24	1419.02
25	1591.97

26	1670.97
27	3040.69
28	3073.0
29	3211.1
30	3221.29
31	3237.91
32	3245.66

Optimised geometry:

C	-0.8495870000	-0.0852120000	-0.2158950000
C	-0.5563240000	1.3731840000	-0.0325900000
C	0.7626540000	1.5447160000	0.1158560000
C	1.4293680000	0.2384070000	0.0441400000
C	0.5090990000	-0.7142720000	-0.1475800000
H	-1.5167990000	-0.4685740000	0.5620580000
H	-1.3110670000	2.1437680000	-0.0259900000
H	1.2699530000	2.4857070000	0.2654330000
H	0.6887470000	-1.7736920000	-0.2416750000
H	2.4943510000	0.0867190000	0.1335180000
H	-1.3439060000	-0.2848280000	-1.1715350000

C_5H_6 (cation, doublet)

Zero-point corrected energy (Eh): -193.67993945

Mode	Frequency (cm^{-1})
6	326.28
7	439.27
8	642.5
9	800.44
10	807.85
11	825.04
12	900.48
13	943.08

14	968.42
15	1015.59
16	1042.3
17	1084.87
18	1120.59
19	1135.15
20	1139.96
21	1307.88
22	1339.89
23	1387.8
24	1461.59
25	1466.56
26	1506.57
27	2992.36
28	3129.73
29	3182.24
30	3200.56
31	3219.32
32	3233.9

Optimised geometry:

C	0.2226360000	-0.6571610000	-0.1609940000
C	-0.7285260000	0.4748220000	-0.0177240000
C	1.5355480000	0.0373980000	-0.1398160000
C	1.3454370000	1.4090820000	0.0002150000
C	-0.0290280000	1.6746400000	0.0743060000
H	-0.4764950000	2.6501570000	0.1848620000
H	-1.8033770000	0.3720030000	0.0098380000
H	0.0636740000	-1.2216750000	-1.0859230000
H	2.4918850000	-0.4578880000	-0.2219320000
H	0.1248570000	-1.3898050000	0.6471600000
H	2.1321320000	2.1461470000	0.0441900000

C_5H_7 (neutral, doublet)

Zero-point corrected energy (Eh): -194.52437237

Mode	Frequency (cm^{-1})
6	145.71
7	255.01
8	272.46
9	372.48
10	569.58
11	609.19
12	695.18
13	810.78
14	900.01
15	908.04
16	919.83
17	992.06
18	1011.36
19	1020.47
20	1121.04
21	1171.11
22	1265.33
23	1318.26
24	1406.78
25	1441.61
26	1467.85
27	1569.12
28	1615.58
29	3145.19
30	3149.24
31	3170.74

32	3174.11
33	3184.03
34	3258.36
35	3268.33

Optimised geometry:

C	1.8810040000	-0.5692120000	0.2133120000
C	0.8671710000	-1.2927980000	-0.3125000000
C	-0.5134170000	-0.9639490000	-0.3530930000
C	-1.1331900000	0.2947110000	-0.1353250000
C	-0.5551830000	1.5168300000	-0.1213040000
H	2.8915890000	-0.9494280000	0.1863550000
H	1.1176520000	-2.2681340000	-0.7165060000
H	-1.1836000000	-1.7831350000	-0.5803590000
H	-1.1542920000	2.3998940000	0.0455530000
H	0.4925130000	1.6649770000	-0.3310380000
H	-2.2105110000	0.2592610000	-0.0115040000
H	1.7208180000	0.3674750000	0.7237860000

C_5H_7 (cation, singlet)

Zero-point corrected energy (Eh): -194.29934087

Mode	Frequency (cm^{-1})
6	184.45
7	374.87
8	569.57
9	791.54
10	818.19
11	825.64
12	878.58
13	932.79
14	949.48
15	1028.13

16	1045.98
17	1046.19
18	1121.94
19	1139.75
20	1159.05
21	1204.81
22	1299.09
23	1319.48
24	1346.29
25	1396.67
26	1411.5
27	1502.56
28	1539.2
29	3040.8
30	3043.15
31	3066.96
32	3070.81
33	3213.13
34	3214.68
35	3260.24

Optimised geometry:

C	-4.3491480000	2.7616590000	-0.0860730000
C	-4.3922790000	1.3846390000	0.0199770000
C	-3.0542480000	0.7894950000	0.1103360000
C	-2.1155290000	1.9887380000	0.0485440000
C	-3.0196370000	3.1379590000	-0.0713740000
H	-5.1973720000	3.4215820000	-0.1656820000
H	-5.3030200000	0.7988860000	0.0357230000
H	-2.9076350000	0.0666680000	-0.6993890000
H	-1.4187090000	1.9669680000	-0.7961830000
H	-1.4778610000	2.1013070000	0.9319310000
H	-2.6709380000	4.1609660000	-0.1397540000
H	-2.9645040000	0.1999890000	1.0290190000

C_6H_4O (neutral, triplet)

Zero-point corrected energy (Eh): -306.05129626

Mode	Frequency (cm^{-1})
6	171.94
7	389.54
8	431.77
9	490.26
10	514.24
11	586.82
12	680.01
13	772.55
14	811.69
15	858.14
16	952.17
17	957.71
18	1004.75
19	1018.4
20	1110.38
21	1146.39
22	1196.33
23	1301.93
24	1359.19
25	1415.54
26	1492.81
27	1547.17
28	1601.46
29	3185.46
30	3194.09

31	3205.97
32	3215.55

Optimised geometry:

O	-0.6732080000	-1.1136040000	0.0231100000
C	-0.7140950000	1.2478990000	0.0082040000
C	-0.0096020000	2.4153330000	-0.0005340000
C	1.3998980000	2.4150720000	-0.0032320000
C	2.1133080000	1.2076430000	0.0031190000
C	1.3926980000	0.0596950000	0.0117580000
C	-0.0442410000	-0.0501670000	0.0150010000
H	-1.7952150000	1.2267330000	0.0104370000
H	-0.5332010000	3.3611070000	-0.0054870000
H	1.9371440000	3.3532480000	-0.0102190000
H	3.1950130000	1.2073480000	0.0011330000

C_6H_4O (cation, doublet)

Zero-point corrected energy (Eh): -305.73509299

Mode	Frequency (cm^{-1})
6	205.73
7	338.44
8	428.04
9	431.48
10	478.32
11	547.22
12	597.62
13	638.51
14	675.29
15	823.6
16	884.48
17	892.9
18	994.04

19	1054.06
20	1121.94
21	1170.88
22	1235.95
23	1352.79
24	1395.99
25	1453.09
26	1468.61
27	1573.13
28	1930.08
29	3193.85
30	3232.6
31	3252.32
32	3732.47

Optimised geometry:

O	2.2333620000	-0.1117500000	-0.0788970000
C	0.9333030000	-0.0838010000	-0.0893520000
C	0.1441570000	-1.2950130000	0.0655550000
C	-1.2257640000	-1.2702800000	0.0550350000
C	-1.6638400000	0.0513560000	-0.1206180000
C	-0.9996390000	1.0936030000	-0.2541730000
C	0.3421340000	1.2346350000	-0.2656240000
H	2.6079860000	-0.9991610000	0.0395140000
H	0.6771560000	-2.2292720000	0.1913300000
H	-1.8457620000	-2.1451170000	0.1669770000
H	0.9595710000	2.1135520000	-0.3773690000

C_6H_4 (neutral, singlet)

Zero-point corrected energy (Eh): -230.80588715

Mode	Frequency (cm^{-1})
6	310.16

7	570.89
8	594.37
9	615.75
10	633.67
11	798.84
12	813.5
13	818.29
14	837.54
15	910.55
16	1001.0
17	1064.41
18	1069.53
19	1109.22
20	1148.61
21	1292.22
22	1365.01
23	1429.87
24	1574.36
25	1859.39
26	3181.29
27	3206.3
28	3240.52
29	3244.64

Optimised geometry:

C	-1.3203370000	0.0083580000	0.0753190000
C	-0.7795920000	1.3044500000	0.0335240000
C	0.6218400000	1.2860730000	-0.0638970000
C	1.0182660000	-0.0358580000	-0.0861490000
C	0.9782730000	-1.3798750000	-0.0780060000
C	-0.2679180000	-0.8819300000	0.0059940000
H	-2.3653250000	-0.2419700000	0.1482930000

H	-1.3729330000	2.2105560000	0.0712600000
H	1.2666290000	2.1474090000	-0.1121200000
H	1.5695110000	-2.2827910000	-0.1154060000

C_6H_4 (cation, doublet)

Zero-point corrected energy (Eh): -230.47680062

Mode	Frequency (cm^{-1})
8	261.16
9	371.97
10	414.45
11	654.96
12	739.75
13	795.73
14	947.66
15	975.28
16	1007.39
17	1023.45
18	1033.53
19	1102.76
20	1201.23
21	1308.15
22	1384.25
23	1403.94
24	1503.66
25	2047.39
26	3168.12
27	3179.35
28	3234.61
29	3325.44

Optimised geometry:

C	-5.6238990000	3.1029200000	0.0601590000
C	-5.7324800000	1.7375080000	-0.0615890000
C	-4.5699240000	0.8989130000	-0.0595940000
C	-3.4085340000	1.6328390000	-0.1493810000
C	-3.3124260000	2.8389550000	0.1527170000
C	-4.3432470000	3.7470450000	0.0604500000
H	-6.7048750000	1.2663650000	-0.0825060000
H	-4.6296020000	-0.1662590000	0.1235890000
H	-6.5095180000	3.7220000000	0.0792980000
H	-4.2335340000	4.8082050000	-0.1231440000

C_6H_5O (neutral, doublet)

Zero-point corrected energy (Eh): -306.68050122

Mode	Frequency (cm^{-1})
6	244.29
7	318.48
8	405.37
9	406.73
10	486.29
11	538.89
12	612.98
13	692.76
14	800.57
15	820.04
16	824.56
17	938.28
18	971.47
19	1001.82
20	1058.38
21	1102.61

22	1170.59
23	1192.15
24	1292.35
25	1309.93
26	1319.75
27	1454.8
28	1498.96
29	1635.24
30	1677.35
31	3164.54
32	3189.99
33	3198.29
34	3210.5
35	3853.53

Optimised geometry:

C	0.0058280000	2.1283110000	-0.0165300000
C	-1.1992480000	1.4343200000	0.0031050000
C	-1.1959530000	0.0437530000	-0.0062390000
C	0.0229400000	-0.5818840000	-0.0350390000
C	1.2326890000	0.0539650000	-0.0552850000
C	1.2174210000	1.4483820000	-0.0456440000
H	2.1704710000	-0.4835210000	-0.0779330000
H	2.1492650000	2.0019120000	-0.0608040000
H	-2.1269200000	-0.5057400000	0.0088860000
O	-0.0633930000	3.4931350000	-0.0067010000
H	0.8276080000	3.8519240000	-0.0235860000
H	-2.1240800000	1.9934820000	0.0254990000

C_6H_5O (cation, singlet)

Zero-point corrected energy (Eh): -306.41102094

Mode	Frequency (cm^{-1})

6	131.14
7	296.98
8	358.31
9	429.76
10	512.67
11	559.48
12	590.09
13	761.86
14	796.05
15	860.62
16	981.14
17	997.14
18	1044.23
19	1064.58
20	1087.24
21	1106.51
22	1172.98
23	1187.17
24	1268.05
25	1405.03
26	1435.76
27	1494.76
28	1597.12
29	1678.35
30	1788.29
31	3191.88
32	3215.87
33	3217.6
34	3231.94
35	3234.62

Optimised geometry:

O	0.0499120000	2.2550630000	-0.0036170000
C	-0.9796860000	1.6286990000	-0.0062280000
C	-0.9663460000	0.1382990000	-0.0105100000
C	-2.2969390000	2.3262170000	-0.0053110000
C	-2.1333250000	-0.5422660000	-0.0134710000
C	-3.3469390000	0.1891800000	-0.0124020000
C	-3.4380970000	1.6031760000	-0.0083810000
H	0.0054740000	-0.3372590000	-0.0110620000
H	-2.1645070000	-1.6214850000	-0.0166200000
H	-2.2720340000	3.4078550000	-0.0021580000
H	-4.4107110000	2.0719390000	-0.0078550000
H	-4.2733460000	-0.3742180000	-0.0148390000

C_6H_5O (anion, singlet)

Zero-point corrected energy (Eh): -306.79665112

Mode	Frequency (cm^{-1})
6	193.92
7	432.11
8	447.74
9	505.04
10	531.54
11	615.51
12	700.62
13	707.91
14	806.66
15	836.04
16	859.17
17	944.89
18	952.24
19	983.24
20	1035.47

21	1072.87
22	1146.94
23	1161.95
24	1241.85
25	1336.07
26	1415.6
27	1489.71
28	1575.23
29	1583.8
30	1657.62
31	3106.96
32	3110.2
33	3149.72
34	3151.24
35	3173.61

Optimised geometry:

C	-0.0389500000	-0.0662450000	0.0000100000
C	1.3999610000	0.0164780000	0.0000020000
C	2.0782330000	1.2179030000	-0.0000010000
C	1.4086120000	2.4429500000	0.0000010000
C	0.0129140000	2.4092660000	0.0000000000
C	-0.6876830000	1.2207410000	0.0000000000
H	1.9417600000	-0.9235730000	0.0000030000
H	3.1655610000	1.2080260000	-0.0000030000
H	1.9493840000	3.3808880000	-0.0000010000
H	-0.5398950000	3.3456690000	-0.0000010000
H	-1.7726580000	1.2194390000	0.0000010000
O	-0.6682100000	-1.1570290000	-0.0000110000

C_6H_5 (anion, singlet)

Zero-point corrected energy (Eh): -231.48086545

Mode	Frequency (cm^{-1})
6	366.85
7	402.76
8	597.91
9	631.61
10	687.66
11	742.02
12	865.83
13	871.43
14	980.04
15	982.73
16	988.11
17	1012.45
18	1056.63
19	1070.22
20	1147.87
21	1198.2
22	1245.71
23	1336.75
24	1433.28
25	1461.65
26	1601.14
27	1603.43
28	3017.19
29	3018.55
30	3087.03
31	3093.0
32	3144.68

Optimised geometry:

C	-0.0526570000	-0.0847410000	-0.0000000000
C	1.3577970000	0.0248590000	0.0000020000
C	2.0765670000	1.2203450000	0.0000030000
C	1.4054900000	2.4377500000	0.0000010000
C	0.0156230000	2.4116150000	0.0000010000
C	-0.6616390000	1.1921580000	0.0000000000
H	1.9568550000	-0.8901160000	-0.0000040000
H	3.1657350000	1.2067170000	0.0000020000
H	1.9484670000	3.3773380000	-0.0000030000
H	-0.5397940000	3.3486500000	-0.0000010000
H	-1.7534920000	1.2545280000	0.0000010000

C_6H_5 (neutral, doublet)

Zero-point corrected energy (Eh): -231.44816475

Mode	Frequency (cm^{-1})
6	397.28
7	424.48
8	598.1
9	617.24
10	685.09
11	729.74
12	831.61
13	909.48
14	988.65
15	990.73
16	1016.73
17	1037.21
18	1062.08
19	1082.22
20	1171.67
21	1178.87
22	1299.16

23	1319.36
24	1474.65
25	1481.4
26	1611.24
27	1666.21
28	3178.82
29	3184.75
30	3197.88
31	3201.34
32	3211.88

Optimised geometry:

C	-1.3050850000	-0.0739730000	0.0747350000
C	-0.6897170000	1.1699160000	0.0273890000
C	0.6986850000	1.2627180000	-0.0689640000
C	1.3869050000	0.0807550000	-0.1121100000
C	0.8386960000	-1.1723230000	-0.0693050000
C	-0.5511790000	-1.2391070000	0.0271320000
H	-2.3816910000	-0.1359050000	0.1493690000
H	-1.2853560000	2.0723420000	0.0652130000
H	1.1929100000	2.2235490000	-0.1068130000
H	1.4396960000	-2.0702290000	-0.1074930000
H	-1.0395740000	-2.2038120000	0.0647180000

C_6H_5 (cation, singlet)

Zero-point corrected energy (Eh): -231.15281234

Mode	Frequency (cm^{-1})
6	392.87
7	406.47
8	407.68
9	476.42
10	562.55

11	659.39
12	709.11
13	868.19
14	894.56
15	965.18
16	987.0
17	1001.23
18	1015.61
19	1088.71
20	1103.48
21	1146.36
22	1181.22
23	1296.92
24	1373.81
25	1498.63
26	1508.92
27	1826.0
28	3205.91
29	3208.95
30	3240.62
31	3250.84
32	3253.99

Optimised geometry:

C	-0.3888240000	2.1387630000	-0.0000020000
C	-1.6558820000	1.7798880000	0.0000010000
C	-1.5897750000	0.3569110000	-0.0000000000
C	-0.3792550000	-0.3191780000	-0.0000090000
C	0.8259680000	0.3663110000	-0.0000020000
C	0.8809870000	1.7897700000	-0.0000020000
H	-0.3750540000	-1.3984210000	-0.0000050000
H	1.7808470000	-0.1420820000	0.0000050000
H	-2.5657860000	2.3583410000	0.0000040000

H	-2.5406620000	-0.1589100000	0.0000050000
H	1.7863570000	2.3752960000	0.0000020000

C_6H_6O (neutral, singlet)

Zero-point corrected energy (Eh): -307.35546440

Mode	Frequency (cm^{-1})
6	60.01
7	270.98
8	354.87
9	404.77
10	494.78
11	533.9
12	607.7
13	683.71
14	736.51
15	772.76
16	815.99
17	849.4
18	936.79
19	951.74
20	960.56
21	982.69
22	1041.7
23	1109.91
24	1125.52
25	1145.56
26	1266.71
27	1320.99
28	1411.17

29	1526.4
30	1540.78
31	1613.61
32	1715.29
33	3239.72
34	3255.43
35	3266.35
36	3275.23
37	3282.29
38	3889.54

Optimised geometry:

C	-0.9481890000	8.6633910000	-1.6782480000
C	-0.7526380000	7.6369020000	-2.5858220000
C	-0.6173330000	6.3233740000	-2.1548490000
C	-0.6801760000	6.0458010000	-0.7987230000
C	-0.8758040000	7.0656150000	0.1212610000
C	-1.0098230000	8.3750770000	-0.3207040000
O	-1.2042240000	9.4201280000	0.5352310000
H	-1.0548790000	9.6895900000	-1.9992860000
H	-0.7052890000	7.8661360000	-3.6415710000
H	-0.4647610000	5.5269460000	-2.8686260000
H	-0.5766570000	5.0283150000	-0.4476030000
H	-0.9245510000	6.8452210000	1.1811030000
H	-1.2251360000	9.0863350000	1.4359300000

C_6H_6O (cation, doublet)

Zero-point corrected energy (Eh): -307.05111280

Mode	Frequency (cm^{-1})
6	177.87
7	359.22
8	414.34
9	439.83

10	525.52
11	561.42
12	626.22
13	630.92
14	812.43
15	822.8
16	829.2
17	964.19
18	998.24
19	1005.21
20	1029.98
21	1041.17
22	1108.19
23	1158.01
24	1192.3
25	1207.1
26	1371.7
27	1402.96
28	1410.82
29	1479.72
30	1530.93
31	1564.29
32	1694.01
33	3202.32
34	3217.4
35	3224.52
36	3232.34
37	3238.28
38	3735.1

Optimised geometry:

O	2.2407400000	-0.1064000000	-0.0920480000
C	0.9380820000	-0.1086580000	-0.0754600000
C	0.1732110000	-1.3063970000	0.0529860000
C	-1.1827330000	-1.2128870000	0.0608250000
C	-1.8065560000	0.0539370000	-0.0569180000
C	-1.0503230000	1.2372100000	-0.1837780000
C	0.3087090000	1.1707680000	-0.1944050000
H	2.6252150000	-0.9932080000	-0.0087010000
H	0.6791330000	-2.2587660000	0.1411590000
H	-1.7921870000	-2.0991220000	0.1564140000
H	-2.8857470000	0.1111250000	-0.0489720000
H	-1.5536380000	2.1882150000	-0.2716740000
H	0.9406550000	2.0418340000	-0.2886980000

C_6H_6 (neutral, singlet)

Zero-point corrected energy (Eh): -232.12060333

Mode	Frequency (cm^{-1})
6	414.17
7	414.58
8	619.31
9	620.21
10	694.73
11	728.91
12	876.05
13	877.63
14	1003.33
15	1004.43
16	1025.04
17	1033.82
18	1034.35
19	1072.07

20	1072.16
21	1164.9
22	1198.52
23	1200.36
24	1320.81
25	1382.95
26	1521.26
27	1523.76
28	1672.5
29	1673.26
30	3177.49
31	3187.15
32	3187.8
33	3204.34
34	3204.56
35	3214.91

Optimised geometry:

C	-1.3698360000	-0.0754240000	0.1800920000
C	-0.7804990000	1.1740690000	0.0511270000
C	0.5742500000	1.2764340000	-0.2308060000
C	1.3395590000	0.1292190000	-0.3838030000
C	0.7502120000	-1.1203030000	-0.2548440000
C	-0.6044920000	-1.2226540000	0.0270930000
H	-2.4256590000	-0.1550690000	0.3997590000
H	-1.3769180000	2.0681730000	0.1704280000
H	1.0334220000	2.2502930000	-0.3313500000
H	1.3467880000	-2.0142940000	-0.3741950000
H	-1.0638310000	-2.1964320000	0.1276750000
H	2.3953800000	0.2088590000	-0.6034830000

C_6H_6 (cation, doublet)

Zero-point corrected energy (Eh): -231.73065145

Mode	Frequency (cm^{-1})
6	184.02
7	311.73
8	447.04
9	561.64
10	580.73
11	692.74
12	794.18
13	863.17
14	896.87
15	964.83
16	1002.2
17	1007.21
18	1023.1
19	1059.33
20	1144.03
21	1160.36
22	1187.4
23	1274.76
24	1319.38
25	1407.01
26	1447.83
27	1473.34
28	1558.95
29	1616.02
30	2980.23
31	2989.28
32	3200.81
33	3203.88
34	3217.78

35	3222.07

Optimised geometry:

C	-0.6126250000	2.2474860000	0.2083000000
C	0.5626030000	1.7120250000	-0.2892940000
C	1.1885950000	0.8377230000	0.5508460000
C	0.6281410000	0.5181220000	1.8671610000
H	2.1164070000	0.3595080000	0.2625130000
H	0.9491540000	1.9686820000	-1.2649030000
C	-0.6278740000	1.1677790000	2.2535900000
C	-1.2566690000	2.0425550000	1.4160470000
H	0.5160300000	-0.5754970000	1.9595930000
H	-1.0444540000	0.9337010000	3.2253500000
H	-2.1825550000	2.5376120000	1.6704950000
H	1.3895370000	0.7073840000	2.6428130000

C_6H_7O (neutral, doublet)

Zero-point corrected energy (Eh): -307.88747959

Mode	Frequency (cm^{-1})
6	132.46
7	283.9
8	398.22
9	427.19
10	473.72
11	498.02
12	531.54
13	583.27
14	664.1
15	748.66
16	797.17
17	873.64
18	942.16

19	948.24
20	978.14
21	983.84
22	1017.05
23	1120.18
24	1182.3
25	1186.37
26	1189.49
27	1277.71
28	1323.32
29	1364.61
30	1442.04
31	1445.33
32	1477.55
33	1572.53
34	1654.62
35	2972.16
36	2974.52
37	3159.06
38	3180.64
39	3199.79
40	3223.52
41	3828.41

Optimised geometry:

O	2.3273680000	-0.1117970000	0.0638230000
C	0.9679610000	-0.1234200000	0.0319580000
C	0.2149480000	-1.2507990000	0.0356630000
C	-1.1968700000	-1.1748510000	0.0009330000
C	-1.8258160000	0.0884180000	-0.0378920000
C	-1.1086980000	1.2390760000	-0.0429020000
C	0.3873370000	1.2478460000	-0.0079560000

H	2.6478150000	-1.0189230000	0.0879500000
H	0.7032510000	-2.2185670000	0.0657300000
H	-1.7847730000	-2.0796150000	0.0042150000
H	-2.9063770000	0.1362290000	-0.0641640000
H	-1.6106140000	2.1962760000	-0.0728800000
H	0.7601820000	1.8139300000	0.8572810000
H	0.8008760000	1.7806650000	-0.8758230000

C_6H_7O (cation, singlet)

Zero-point corrected energy (Eh): -307.66466980

Mode	Frequency (cm^{-1})
6	150.15
7	314.42
8	347.98
9	427.83
10	513.19
11	569.45
12	593.03
13	633.26
14	798.92
15	829.1
16	834.4
17	903.15
18	908.36
19	989.2
20	1011.39
21	1016.06
22	1061.44
23	1162.54
24	1186.55
25	1204.87

26	1210.4
27	1340.28
28	1364.49
29	1402.97
30	1427.82
31	1534.76
32	1547.5
33	1624.73
34	1705.39
35	3010.36
36	3027.94
37	3197.88
38	3208.26
39	3214.01
40	3236.17
41	3750.87

Optimised geometry:

C	-4.2824270000	2.7687220000	0.0226940000
C	-4.3327160000	1.4247600000	0.0060670000
C	-3.1161890000	0.5960950000	0.0029650000
C	-1.8404500000	1.3320770000	0.0190590000
C	-3.0086030000	3.4088700000	0.0377760000
H	-5.1805610000	3.3718130000	0.0249140000
H	-5.2887130000	0.9185210000	-0.0055690000
H	-3.1393690000	-0.0826850000	-0.8613940000
H	-0.9260280000	0.7542000000	0.0171890000
H	-0.8609830000	3.2283350000	0.0477210000
O	-2.8922650000	4.7031350000	0.0542960000
H	-3.7458330000	5.1612980000	0.0551040000
H	-3.1490080000	-0.1031520000	0.8505490000
C	-1.7882240000	2.6753870000	0.0357090000

C_6H_7 (neutral, doublet)

Zero-point corrected energy (Eh): -232.64910667

Mode	Frequency (cm^{-1})
6	164.39
7	386.75
8	528.32
9	561.77
10	589.87
11	639.19
12	740.72
13	788.07
14	887.21
15	936.13
16	981.94
17	989.32
18	993.13
19	994.67
20	999.87
21	1102.79
22	1168.33
23	1187.8
24	1200.12
25	1315.09
26	1368.25
27	1421.32
28	1432.37
29	1462.95
30	1567.34
31	1633.3
32	2956.13

33	2961.23
34	3174.11
35	3175.54
36	3193.37
37	3194.98
38	3219.1

Optimised geometry:

C	1.5547130000	-0.2783380000	-0.1320460000
C	0.6893880000	-1.3167440000	-0.2239440000
C	-0.6988360000	-1.1400290000	-0.0238790000
C	-1.1992800000	0.1469530000	0.2789440000
C	-0.3773260000	1.2189740000	0.3824260000
C	1.1002880000	1.1101570000	0.1844310000
H	2.6133940000	-0.4367360000	-0.2889920000
H	1.0662140000	-2.3045080000	-0.4555870000
H	-1.3713490000	-1.9808670000	-0.1017140000
H	-0.7826120000	2.1947050000	0.6153370000
H	1.6262150000	1.4820030000	1.0763460000
H	1.4237140000	1.8000480000	-0.6091890000
H	-2.2630620000	0.2755710000	0.4309870000

C_6H_7 (cation, singlet)

Zero-point corrected energy (Eh): -232.40512411

Mode	Frequency (cm^{-1})
6	186.92
7	330.16
8	407.77
9	583.96
10	592.48
11	657.24
12	818.29
13	851.99

14	913.78
15	993.77
16	1003.78
17	1024.9
18	1029.24
19	1066.17
20	1087.79
21	1152.72
22	1157.11
23	1205.91
24	1213.8
25	1267.04
26	1363.88
27	1438.31
28	1486.1
29	1494.12
30	1598.91
31	1670.51
32	2977.56
33	2986.91
34	3197.49
35	3209.77
36	3211.59
37	3233.76
38	3236.54

Optimised geometry:

C	1.5601990000	-0.2764890000	-0.1365190000
C	0.7012940000	-1.3272910000	-0.2279090000
C	-0.6686940000	-1.1004550000	-0.0176490000
C	-1.2045410000	0.1622510000	0.2840680000
C	-0.3612290000	1.2252500000	0.3797130000

C	1.0767180000	1.0668860000	0.1742700000
H	2.6222590000	-0.4121320000	-0.2919980000
H	1.0538390000	-2.3212720000	-0.4571320000
H	-1.3464750000	-1.9420850000	-0.0920100000
H	-0.7368780000	2.2132400000	0.6106530000
H	1.6199580000	1.4606640000	1.0505320000
H	1.4184980000	1.7714620000	-0.6032930000
H	-2.2672700000	0.2743500000	0.4351610000

CH_2O (neutral, singlet)

Zero-point corrected energy (Eh): -114.39532349

Mode	Frequency (cm^{-1})
6	1092.76
7	1219.19
8	1356.34
9	1513.76
10	2861.06
11	3749.67

Optimised geometry:

O	2.3423620000	0.0315420000	-0.1203110000
C	1.0435800000	0.1682040000	-0.2004150000
H	0.9408370000	1.2247960000	-0.5341820000
H	2.5329250000	-0.8706760000	0.1673540000

CH_2O (cation, doublet)

Zero-point corrected energy (Eh): -114.07223454

Mode	Frequency (cm^{-1})
6	930.77
7	977.39
8	1223.44

9	1736.31
10	3032.36
11	3474.97

Optimised geometry:

O 2.3320020000 -0.0312560000 -0.0814630000
C 1.1676240000 0.2728980000 -0.2475230000
H 0.8154860000 1.2697480000 -0.5534470000
H 2.5445930000 -0.9575250000 0.1948790000

CH_2 (neutral, triplet)

Zero-point corrected energy (Eh): -39.11967157

Mode	Frequency (cm^{-1})
6	1025.05
7	3151.94
8	3394.12

Optimised geometry:

C -1.3474430000 -1.3917300000 0.1596870000
H -1.3367360000 -0.3283900000 0.3196280000
H -0.7152580000 -2.2213800000 0.4212310000

CH_2 (cation, doublet)

Zero-point corrected energy (Eh): -38.73846172

Mode	Frequency (cm^{-1})
6	1218.79
7	3007.7
8	3317.86

Optimised geometry:

C -1.8657380000 2.2706160000 -0.1353030000

H	-0.9759200000	2.8463960000	0.1178670000
H	-2.7553520000	1.6946470000	-0.3887540000

CH (neutral, doublet)

Zero-point corrected energy (Eh): -38.46671290

Mode	Frequency (cm^{-1})
5	2855.61

Optimised geometry:

C	-1.4825090000	-1.4518240000	0.0695500000
H	-1.2315540000	-0.4025910000	0.3723560000

CH (cation, singlet)

Zero-point corrected energy (Eh): -38.07606343

Mode	Frequency (cm^{-1})
5	2803.64

Optimised geometry:

C	-1.4841300000	-1.4586000000	0.0675930000
H	-1.2299330000	-0.3958150000	0.3743130000

COH (neutral, doublet)

Zero-point corrected energy (Eh): -113.78016566

Mode	Frequency (cm^{-1})
6	1145.79
7	1422.31
8	3400.08

Optimised geometry:

C	0.5765520000	1.9845130000	-0.1152270000
O	-0.6230990000	1.9770770000	0.3003850000
H	-1.2232030000	2.5605600000	-0.2125780000

COH (cation, singlet)

Zero-point corrected energy (Eh): -113.49169827

Mode	Frequency (cm^{-1})
6	154.18
7	2000.39
8	3452.65

Optimised geometry:

O	2.0734010000	-0.4318060000	0.0619530000
C	1.0317480000	0.0658200000	0.0291120000
H	2.9693820000	-0.8600460000	0.0900280000

COH (anion, singlet)

Zero-point corrected energy (Eh): -113.84275006

Mode	Frequency (cm^{-1})
6	1080.85
7	1273.87
8	1764.89

Optimised geometry:

O	2.0805550000	1.3658990000	0.4437660000
C	1.1625140000	0.5980290000	0.1829830000
H	0.0842420000	1.2934850000	-0.0036770000

CO (neutral, singlet)

Zero-point corrected energy (Eh): -113.32777161

Mode	Frequency (cm^{-1})
5	2236.22

Optimised geometry:

C	-1.3791030000	3.7499440000	0.0000000000
O	-0.2546370000	3.7009160000	0.0000000000

CO (cation, doublet)

Zero-point corrected energy (Eh): -112.80637477

Mode	Frequency (cm^{-1})
5	2325.76

Optimised geometry:

O	2.2059550000	-0.1142720000	0.0585380000
C	1.0981880000	-0.1268130000	0.0342160000

H_2O (neutral, singlet)

Zero-point corrected energy (Eh): -76.40470825

Mode	Frequency (cm^{-1})
6	1640.11
7	3830.4
8	3930.47

Optimised geometry:

O	2.3520420000	-0.0184170000	0.2189340000
H	2.6529160000	-0.8897730000	-0.0484960000
H	1.8220180000	0.2866720000	-0.5208630000

H_2O (cation, doublet)

Zero-point corrected energy (Eh): -75.95148401

Mode	Frequency (cm^{-1})
6	1450.4
7	3363.91
8	3406.95

Optimised geometry:

O	2.4540730000	-0.2843270000	0.1432830000
H	2.5994420000	-1.1534600000	-0.3346140000
H	2.9711180000	-0.2828640000	1.0021120000

H (neutral, doublet)

Electronic energy (Eh): -0.494118969290

Geometry:

H	0	0	0

H_2 (neutral, singlet)

Zero-point corrected energy (Eh): -1.15133732

Mode	Frequency (cm^{-1})
5	4454.32

Optimised geometry:

H	-0.4525460000	-0.4190350000	-0.0087500000
H	-0.0509640000	-0.5704650000	0.5940100000

H_2 (cation, doublet)

Zero-point corrected energy (Eh): -0.58960526

Mode	Frequency (cm^{-1})
5	1873.72

Optimised geometry:

H	-0.5599050000	-0.3785520000	-0.1698910000

H 0.0563950000 -0.6109480000 0.7551510000

H_3 (neutral, doublet)

Zero-point corrected energy (Eh): -1.63303365

Mode	Frequency (cm^{-1})
6	848.53
7	848.68
8	2070.66

Optimised geometry:

H -0.1866400000 -0.0000000000 -0.0000000000
H 0.7400010000 0.0000000000 0.0000000000
H 1.6666380000 -0.0000000000 -0.0000000000

H_3 (cation, singlet)

Zero-point corrected energy (Eh): -1.30440400

Mode	Frequency (cm^{-1})
6	2603.98
7	2606.09
8	3402.98

Optimised geometry:

H 0.0000000000 0.4216270000 0.0000000000
H -0.4376950000 -0.3363870000 0.0000000000
H 0.4376960000 -0.3363870000 0.0000000000

HCO (neutral, doublet)

Zero-point corrected energy (Eh): -113.84466362

Mode	Frequency (cm^{-1})
6	1099.02

Mode	Frequency (cm^{-1})
7	1970.51
8	2649.8

Optimised geometry:

```
C    0.0139870000   -0.0116850000    0.0000000000
O    1.1847610000   -0.0055340000    0.0000000000
H   -0.6187480000    0.9172190000    0.0000000000
```

HCO (cation, singlet)

Zero-point corrected energy (Eh): -113.54730295

Mode	Frequency (cm^{-1})
5	690.85
6	699.36
7	1791.21
8	3431.34

Optimised geometry:

```
O    0.6182620000    2.2981790000    0.0015350000
C   -0.4808600000    2.2275800000   -0.0039160000
H   -1.5727240000    2.1575340000   -0.0093420000
```

HCO (anion, singlet)

Zero-point corrected energy (Eh): -113.76296617

Mode	Frequency (cm^{-1})
5	867.34
6	1585.45
7	2345.91
8	3451.48

Optimised geometry:

```
H   -0.0130430000    0.0000000000    0.0000000000
```

C	1.0515310000	0.0000000000	0.0000010000
O	2.2615120000	0.0000000000	0.0000000000

HO (neutral, doublet)

Zero-point corrected energy (Eh): -75.72706775

Mode	Frequency (cm^{-1})
5	3741.13

Optimised geometry:

O	-1.9037180000	2.2459230000	0.1462320000
H	-2.6971920000	1.7323470000	0.3721380000

HO (cation, singlet)

Zero-point corrected energy (Eh): -75.15116879

Mode	Frequency (cm^{-1})
5	3072.31

Optimised geometry:

O	2.3067990000	-0.0893790000	0.0602800000
H	2.7826110000	-1.0085590000	0.0890800000

HO (anion, singlet)

Zero-point corrected energy (Eh): -75.74988454

Mode	Frequency (cm^{-1})
5	3703.34

Optimised geometry:

O	-0.0028680000	0.0000000000	0.0000000000
H	0.9670340000	0.0000000000	0.0000000000

H_2 loss transition state

Zero-point corrected energy (Eh): -307.50402235

Mode	Frequency (cm^{-1})
6	-1720.71
7	114.47
8	189.48
9	368.97
10	391.54
11	438.12
12	525.96
13	601.89
14	606.17
15	644.66
16	777.85
17	804.34
18	820.4
19	846.15
20	968.28
21	1016.85
22	1021.23
23	1033.64
24	1034.65
25	1066.92
26	1122.43
27	1193.02
28	1198.68
29	1335.62
30	1363.77

31	1395.83
32	1497.11
33	1522.28
34	1604.65
35	1663.8
36	2918.81
37	3211.28
38	3215.54
39	3217.92
40	3233.94
41	3237.78

Optimised geometry:

O	-3.6632450000	4.7083980000	0.1925180000
C	-2.1478500000	2.9105160000	0.3087600000
C	-3.4607890000	3.4052900000	0.1244210000
C	-1.9808280000	1.5612370000	0.4806910000
C	-3.0964420000	0.7177840000	0.4199100000
C	-4.3900840000	1.2031420000	0.1953160000
C	-4.5822790000	2.5487010000	0.0200470000
H	-2.9526020000	-0.3470870000	0.5422920000
H	-1.3186480000	3.6034580000	0.3453130000
H	-0.9993030000	1.1479900000	0.6558270000
H	-5.2246940000	0.5200190000	0.1552440000
H	-5.5606160000	2.9732180000	-0.1578810000
H	-3.1927850000	5.4382570000	-0.8793700000
H	-4.0554150000	5.3142910000	-0.9952610000

CO loss intermediate 1

Zero-point corrected energy (Eh): -307.60412465

Mode	Frequency (cm^{-1})
6	112.98
7	182.0

8	275.09
9	409.93
10	457.49
11	501.09
12	610.58
13	648.67
14	748.96
15	782.53
16	887.34
17	962.59
18	975.62
19	1009.8
20	1030.99
21	1077.24
22	1131.4
23	1132.45
24	1183.23
25	1222.85
26	1251.93
27	1333.32
28	1350.92
29	1386.71
30	1420.03
31	1460.11
32	1530.73
33	1573.34
34	1854.69
35	2966.4
36	3047.54
37	3056.94

38	3162.63
39	3177.35
40	3198.11
41	3223.08

Optimised geometry:

C	0.6466110000	-0.8377580000	-0.0258330000
C	-0.9072360000	0.9500190000	-0.8771920000
C	1.6592760000	0.2833990000	0.2035410000
C	1.1171950000	1.6376390000	0.1213880000
C	-0.1714480000	1.9525640000	-0.3052420000
H	-0.5353560000	2.9684600000	-0.2550390000
H	-1.8896160000	1.1218850000	-1.3024570000
H	1.1203250000	-1.7823700000	-0.2741640000
H	2.4714620000	0.2512020000	-0.5440450000
H	2.1980180000	0.1883760000	1.1519390000
H	1.7793250000	2.4490150000	0.4072180000
H	0.0493010000	-0.9712800000	0.8815600000
C	-0.2746530000	-0.3941450000	-1.1166800000
O	-0.4659600000	-0.9065790000	-2.1825000000

CO loss transition state 1

Zero-point corrected energy (Eh): -307.55502755

Mode	Frequency (cm^{-1})
6	-839.28
7	55.78
8	227.11
9	419.41
10	447.11
11	464.09
12	514.58
13	547.15
14	657.96

15	726.76
16	755.35
17	840.25
18	914.13
19	948.69
20	980.19
21	985.31
22	1037.32
23	1120.91
24	1160.78
25	1177.23
26	1194.66
27	1234.93
28	1341.66
29	1368.25
30	1375.49
31	1388.21
32	1556.23
33	1655.57
34	1832.26
35	2304.5
36	2995.45
37	3085.82
38	3203.31
39	3206.68
40	3229.76
41	3252.01

Optimised geometry:

O	-5.2340930000	3.8025240000	1.1153840000
C	-4.6169010000	2.3049950000	-0.5649910000

C	-3.6095360000	1.5190970000	-1.0444130000
C	-4.3352910000	3.3794340000	0.4433810000
C	-2.1445910000	1.8708490000	-0.7337840000
C	-1.8636970000	2.9724310000	-0.0198270000
C	-2.9075790000	3.8680180000	0.5390710000
H	-3.7875520000	0.6935200000	-1.7173820000
H	-3.1664380000	1.1623630000	0.0210630000
H	-5.6524960000	2.1430690000	-0.8380060000
H	-2.6955370000	4.1476330000	1.5733650000
H	-1.4057770000	1.1893750000	-1.1230100000
H	-0.8281470000	3.2583950000	0.1119890000
H	-2.8460720000	4.8188880000	-0.0127080000

CO loss intermediate 2

Zero-point corrected energy (Eh): -307.63025157

Mode	Frequency (cm^{-1})
6	79.87
7	144.25
8	211.57
9	392.38
10	506.38
11	616.82
12	684.94
13	723.89
14	785.13
15	803.18
16	881.22
17	896.2
18	925.74
19	970.11
20	985.1
21	1038.52
22	1064.33

23	1122.8
24	1160.0
25	1195.61
26	1249.28
27	1285.12
28	1316.05
29	1341.61
30	1368.62
31	1475.71
32	1491.06
33	1690.04
34	2347.91
35	3065.69
36	3072.73
37	3107.57
38	3126.98
39	3159.12
40	3223.74
41	3254.34

Optimised geometry:

C	0.4024090000	-0.5346660000	0.2461220000
C	-0.5581550000	0.3653190000	-0.6037790000
C	1.6833190000	0.3025900000	0.4005940000
C	1.2409270000	1.6998270000	0.0821910000
C	0.0375830000	1.7706710000	-0.4695750000
H	-0.4962370000	2.6482800000	-0.7965080000
H	-1.6136280000	0.2694130000	-0.3357050000
H	0.5611010000	-1.5208660000	-0.1825180000
H	2.4724560000	-0.0110100000	-0.2858920000
H	2.0924340000	0.2200960000	1.4052320000
H	1.8518800000	2.5669400000	0.2877220000
H	-0.0976220000	-0.6647650000	1.2022410000
C	-0.4613830000	0.0057530000	-1.9967650000

O -0.3287590000 -0.2450100000 -3.0758710000

CO loss transition state 2

Zero-point corrected energy (Eh): -307.56767941

Mode	Frequency (cm^{-1})
6	-563.98
7	144.63
8	184.22
9	407.6
10	445.31
11	477.32
12	550.9
13	625.26
14	688.43
15	787.26
16	870.75
17	901.95
18	919.15
19	954.31
20	995.65
21	1007.65
22	1040.91
23	1088.36
24	1138.75
25	1153.17
26	1193.42
27	1255.82
28	1336.48
29	1364.86

30	1399.77
31	1432.96
32	1475.52
33	1641.36
34	2069.41
35	3049.74
36	3085.1
37	3113.8
38	3146.92
39	3167.04
40	3204.98
41	3226.98

Optimised geometry:

C	0.4738080000	-0.6495930000	0.0371720000
C	-0.8528490000	0.7733200000	-0.8012290000
C	1.6747320000	0.2853620000	0.1771650000
C	1.1856320000	1.6941720000	0.0427490000
C	-0.0721850000	1.9033380000	-0.3725220000
H	-0.5685710000	2.8624830000	-0.3090260000
H	-1.8947860000	0.6277220000	-0.5286460000
H	0.6744650000	-1.6876920000	-0.2287090000
H	2.4125000000	0.0629840000	-0.5995750000
H	2.1689310000	0.1434160000	1.1371960000
H	1.8103190000	2.5171990000	0.3601730000
H	-0.1731820000	-0.6262650000	0.9123790000
C	-0.1820290000	-0.2427470000	-1.4844470000
O	0.1295410000	-0.7911290000	-2.4651910000

CO loss transition state 3

Zero-point corrected energy (Eh): -307.61394059

Mode	Frequency (cm^{-1})
6	-342.74

7	73.5
8	88.94
9	171.14
10	321.99
11	379.33
12	508.73
13	709.32
14	774.44
15	809.08
16	837.43
17	918.5
18	943.1
19	973.32
20	1012.73
21	1043.62
22	1075.77
23	1141.68
24	1155.93
25	1180.45
26	1239.19
27	1301.91
28	1314.85
29	1360.22
30	1403.08
31	1441.57
32	1456.28
33	1617.5
34	2313.3
35	3044.47
36	3061.99

37	3121.83
38	3148.62
39	3209.85
40	3223.55
41	3253.06

Optimised geometry:

C	0.3780230000	-0.4944490000	0.3417230000
C	-0.5681360000	0.4200850000	-0.3778480000
C	1.6895150000	0.2980370000	0.3938240000
C	1.2518910000	1.6894260000	0.0885500000
C	-0.0136980000	1.7535660000	-0.3665690000
H	-0.5493970000	2.6393270000	-0.6673580000
H	-1.6339950000	0.2354270000	-0.3623180000
H	0.4436780000	-1.4975980000	-0.0693890000
H	2.4086850000	-0.0370960000	-0.3606490000
H	2.1982760000	0.2264000000	1.3535830000
H	1.8898450000	2.5506440000	0.2309580000
H	-0.0415400000	-0.5925550000	1.3473260000
C	-0.4209500000	-0.1117360000	-2.2669740000
O	-0.2198890000	-0.1968490000	-3.3611830000

Para- to meta-protonated isomerisation transition state

Zero-point corrected energy (Eh): -307.63036240

Mode	Frequency (cm^{-1})
6	-809.97
7	194.08
8	365.44
9	384.51
10	405.13
11	507.6
12	520.8
13	603.64

14	652.75
15	738.91
16	834.17
17	858.74
18	922.22
19	978.6
20	1008.73
21	1023.64
22	1047.36
23	1081.42
24	1154.93
25	1191.72
26	1193.7
27	1208.33
28	1343.63
29	1355.88
30	1406.65
31	1487.86
32	1530.1
33	1602.57
34	1672.63
35	2297.12
36	3185.71
37	3197.84
38	3210.92
39	3223.51
40	3231.64
41	3820.28

Optimised geometry:

O	-2.8903124557	4.7002674674	0.2520506457
C	-1.8645437618	2.6268691787	0.0792899808
C	-3.0161707028	3.3835853630	0.0991434232
C	-1.9530945146	1.2195864610	-0.0672231814
C	-3.2355054508	0.5963238912	-0.2512682946
C	-4.3857167745	1.3892454246	-0.2055011641
C	-4.2726118683	2.7477893860	-0.0464568188
H	-2.4250117464	0.7877620196	0.9652065782
H	-3.7394799644	5.1564475771	0.2361001387
H	-0.9027313600	3.1074323969	0.1784740960
H	-1.0568949264	0.6345380367	-0.2255019154
H	-3.2820562766	-0.4758846096	-0.3755347607
H	-5.3568337781	0.9299852052	-0.3107441482
H	-5.1704064198	3.3534282024	-0.0209535794

Meta- to ortho-protonated isomerisation transition state

Zero-point corrected energy (Eh): -307.62756000

Mode	Frequency (cm^{-1})
6	-811.16
7	211.18
8	394.0
9	399.01
10	407.01
11	482.33
12	524.18
13	615.96
14	667.42
15	755.82
16	840.96
17	860.27
18	949.08
19	993.81
20	1021.51

21	1045.03
22	1056.4
23	1088.9
24	1168.73
25	1195.42
26	1199.71
27	1213.16
28	1329.92
29	1365.61
30	1396.62
31	1483.03
32	1534.16
33	1633.6
34	1655.32
35	2307.48
36	3195.48
37	3207.92
38	3218.07
39	3221.52
40	3239.69
41	3810.12

Optimised geometry:

O	0.3031214815	2.8960964230	0.0712732630
C	1.3214189367	0.8328603165	-0.2413854804
C	1.2596230679	-0.6037031278	-0.3060085882
C	0.0042954178	-1.2548789782	-0.2125935367
C	-1.1221571212	-0.4973977059	-0.0102039876
C	-1.0549486134	0.8964450827	0.0925440901
C	0.1496320619	1.5734754045	-0.0115996612
H	2.1532321853	-1.1450083596	-0.5854021689
H	-0.5370365188	3.3527118025	0.1975866296
H	-0.0450463822	-2.3296625917	-0.2927930190

H	-2.0853628057	-0.9787953362	0.0755956356
H	-1.9644552125	1.4570227075	0.2682550219
H	2.2751970580	1.3354320198	-0.3266026752
H	1.4806898311	-0.1675225104	0.8105250084

Ortho- to O-protonated isomerisation transition state

Zero-point corrected energy (Eh): -307.79381960

Mode	Frequency (cm^{-1})
6	-1882.1
7	229.18
8	265.09
9	416.54
10	450.92
11	483.98
12	532.48
13	609.53
14	661.54
15	712.88
16	783.14
17	832.24
18	905.81
19	963.12
20	1007.3
21	1026.64
22	1043.79
23	1061.13
24	1096.46
25	1138.8
26	1184.91
27	1197.24

28	1241.85
29	1346.08
30	1360.37
31	1496.4
32	1518.61
33	1617.15
34	1664.13
35	1786.97
36	3123.33
37	3212.54
38	3222.6
39	3227.09
40	3238.35
41	3752.16

Optimised geometry:

O	-3.5736128128	4.7438805966	0.0111142797
C	-4.3790778071	2.6337616748	-0.2865820564
C	-4.2662970274	1.2336361951	-0.1862195626
C	-3.0187833563	0.6645691426	-0.0740191963
C	-1.8696148998	1.4635068568	-0.0545140728
C	-1.9386714268	2.8492877929	-0.1032941721
C	-3.1955913184	3.3834649417	-0.2282687378
H	-4.3767309188	3.8160169700	0.5683884816
H	-5.1525733258	0.6216391735	-0.2619287351
H	-2.9183278615	-0.4086771479	-0.0178847689
H	-0.9003536567	0.9937576782	0.0351531713
H	-1.0606887776	3.4726685764	-0.0152670514
H	-2.9627283606	5.1890938966	0.6182396449
H	-5.2988783505	3.0939115227	-0.6491700841

Ortho- to protonated cyclohexa-2,4-dien-1-one isomerisation transition state

Zero-point corrected energy (Eh): -307.55879703

Mode	Frequency (cm^{-1})
6	-1881.08
7	126.52
8	276.73
9	376.31
10	444.16
11	534.88
12	582.97
13	629.58
14	713.05
15	741.5
16	826.91
17	924.54
18	931.76
19	986.78
20	996.92
21	1043.98
22	1050.91
23	1068.16
24	1134.5
25	1146.89
26	1203.76
27	1264.12
28	1321.12
29	1402.81
30	1415.81
31	1448.2
32	1536.45
33	1639.99
34	1679.09

35	1827.92
36	3028.75
37	3120.09
38	3145.69
39	3200.38
40	3205.55
41	3232.67

Optimised geometry:

C	-4.5408460000	2.8932680000	0.3673320000
C	-4.3041710000	1.4680760000	-0.0517630000
C	-3.0845170000	0.9398610000	-0.3034630000
C	-1.8953660000	1.7032820000	-0.0897860000
C	-1.9756030000	3.0532670000	0.1975650000
C	-3.3195270000	3.6288740000	-0.0006950000
H	-4.6975140000	2.9153390000	1.4535280000
H	-5.1854900000	0.8471850000	-0.1462340000
H	-2.9964120000	-0.0960540000	-0.5934400000
H	-0.9316680000	1.2107110000	-0.1333530000
H	-1.2563310000	3.5641170000	0.8340520000
O	-3.2196490000	4.5842590000	-0.7766160000
H	-1.9259020000	4.0406320000	-0.7942590000
H	-5.4247750000	3.3165710000	-0.1092280000

O- to ipso-protonated isomerisation transition state

Zero-point corrected energy (Eh): -307.57246662

Mode	Frequency (cm^{-1})
6	-1649.24
7	205.99
8	241.11
9	368.63
10	398.1
11	442.45

12	511.33
13	610.14
14	663.79
15	742.12
16	789.31
17	844.48
18	875.6
19	979.77
20	1017.09
21	1027.59
22	1044.57
23	1064.94
24	1108.3
25	1122.73
26	1183.13
27	1200.92
28	1208.48
29	1360.66
30	1376.18
31	1505.0
32	1518.12
33	1644.62
34	1654.4
35	2276.93
36	3202.37
37	3215.3
38	3220.63
39	3231.48
40	3236.6
41	3729.96

Optimised geometry:

O	-3.3474480000	4.8958500000	-0.5673190000
C	-4.4809250000	2.6558060000	-0.3404520000
C	-4.3795540000	1.3421090000	0.0589990000
C	-3.1644500000	0.8486420000	0.5361120000
C	-2.0333450000	1.6530150000	0.6249390000
C	-3.3362320000	3.4580530000	-0.2077460000
C	-2.0940570000	2.9720740000	0.2224130000
H	-3.5828960000	4.3545710000	0.6282850000
H	-4.1956730000	5.1183320000	-0.9852520000
H	-5.4099470000	3.0644300000	-0.7152520000
H	-5.2416660000	0.6954480000	-0.0035060000
H	-1.1019630000	1.2471920000	0.9896230000
H	-1.2395030000	3.6329800000	0.2540360000
H	-3.1019130000	-0.1862340000	0.8409840000

Microsolvation Data for Protonated Phenol

Protonated cyclohexa-2,4-dien-1-one + 1H_2O

Zero-point corrected energy (Eh): -384.04481696

Mode	Frequency (cm^{-1})
6	44.81
7	75.76
8	134.95
9	153.06
10	178.27
11	213.52
12	314.12
13	323.48
14	366.24
15	389.86
16	463.65
17	526.06

18	554.4
19	663.57
20	720.93
21	763.16
22	933.12
23	960.67
24	975.44
25	992.95
26	1012.89
27	1057.71
28	1084.82
29	1158.62
30	1184.12
31	1186.4
32	1211.27
33	1325.09
34	1345.45
35	1397.02
36	1415.21
37	1438.55
38	1554.17
39	1594.37
40	1647.21
41	1906.86
42	2990.07
43	2996.99
44	3125.73
45	3159.02
46	3186.28
47	3203.53

48	3231.58
49	3803.18
50	3890.58

Optimised geometry:

O	-1.9773450000	4.0693940000	-2.2916690000
C	-3.6973520000	3.2968000000	-0.8177250000
C	-3.9247900000	1.9215280000	-0.3782020000
C	-2.9456790000	1.1509920000	0.2316900000
C	-1.6446470000	1.5762370000	0.1866650000
C	-2.2547710000	3.4853050000	-1.2973180000
C	-1.2192500000	2.8724410000	-0.3597040000
H	-3.7989430000	3.9458140000	0.0662380000
H	-4.4116690000	3.6286880000	-1.5645640000
H	-1.1340090000	3.5763370000	0.4832420000
H	-4.9117080000	1.4991750000	-0.5203300000
H	-0.8665430000	0.9039720000	0.5303390000
H	-0.2360250000	2.8252260000	-0.8244330000
H	-3.1867330000	0.1706210000	0.6134000000
O	-3.3708300000	1.2734210000	-2.8108260000
H	-3.4784960000	0.3715750000	-3.1287810000
H	-3.3765760000	1.8172920000	-3.6060440000

Protonated cyclohexa-2,4-dien-1-one + 2H_2O

Zero-point corrected energy (Eh): -460.46992030

Mode	Frequency (cm^{-1})
6	14.13
7	28.24
8	92.06
9	102.77
10	104.62
11	152.76
12	189.3
13	193.18

14	212.75
15	231.35
16	271.65
17	298.41
18	331.67
19	344.76
20	372.68
21	393.35
22	459.52
23	531.99
24	544.23
25	667.34
26	717.61
27	747.28
28	932.29
29	968.29
30	982.75
31	997.55
32	1022.32
33	1060.77
34	1095.71
35	1171.9
36	1188.39
37	1192.06
38	1210.2
39	1323.75
40	1340.87
41	1393.85
42	1412.18
43	1445.21

44	1576.54
45	1580.71
46	1647.97
47	1650.01
48	1896.44
49	2936.25
50	2942.61
51	3141.66
52	3144.47
53	3184.1
54	3186.56
55	3231.35
56	3807.39
57	3808.18
58	3893.32
59	3896.85

Optimised geometry:

O	-2.3709640000	4.2578770000	-2.1723990000
C	-4.0310930000	3.2233260000	-0.7967750000
C	-4.1875180000	1.8625310000	-0.3025280000
C	-3.1595140000	1.1125660000	0.2273970000
C	-1.8685480000	1.5701250000	0.0847590000
C	-2.6093740000	3.5337260000	-1.2619990000
C	-1.5237070000	2.9024170000	-0.3936610000
H	-4.1517630000	3.8992410000	0.0702810000
H	-4.7687810000	3.4955720000	-1.5466490000
H	-1.5100570000	3.5682090000	0.4890470000
H	-5.1698110000	1.4100690000	-0.3791900000
H	-1.0532860000	0.8933480000	0.3150310000
H	-0.5469920000	2.9497680000	-0.8682450000
H	-3.3470950000	0.1176400000	0.6009840000
O	-2.7496160000	1.3822480000	-2.8823060000
H	-2.7705620000	0.5001720000	-3.2648080000
H	-2.5871870000	1.9696680000	-3.6281520000

O	-2.8646620000	5.0015280000	1.5361380000
H	-2.7030060000	5.9278280000	1.3287030000
H	-3.0240400000	4.9917050000	2.4850790000

Protonated cyclohexa-2,4-dien-1-one + 3H_2O

Zero-point corrected energy (Eh): -536.89364417

Mode	Frequency (cm^{-1})
6	28.33
7	37.49
8	49.94
9	87.14
10	97.66
11	106.72
12	117.87
13	151.18
14	196.4
15	210.7
16	217.64
17	226.74
18	227.06
19	282.13
20	290.04
21	299.23
22	326.14
23	339.91
24	372.68
25	397.69
26	459.27
27	484.8
28	535.79

29	543.35
30	658.84
31	670.11
32	724.23
33	727.91
34	929.47
35	977.0
36	983.14
37	995.88
38	1023.63
39	1061.57
40	1109.04
41	1172.02
42	1193.5
43	1196.62
44	1213.7
45	1329.38
46	1343.88
47	1376.23
48	1396.61
49	1445.1
50	1579.2
51	1581.59
52	1626.06
53	1648.12
54	1664.04
55	1875.54
56	2879.15
57	2926.86
58	3140.71

59	3146.77
60	3183.96
61	3188.11
62	3231.0
63	3623.54
64	3810.46
65	3812.18
66	3884.63
67	3898.92
68	3904.88

Optimised geometry:

O	-1.8101410000	4.1722830000	-2.2136350000
C	-3.6469020000	3.4559020000	-0.8631220000
C	-4.0515610000	2.1726440000	-0.3091680000
C	-3.1791880000	1.2716360000	0.2615480000
C	-1.8258990000	1.4827770000	0.1191970000
C	-2.1910330000	3.4988120000	-1.3059640000
C	-1.2438200000	2.6990780000	-0.4254860000
H	-3.6630990000	4.1730780000	-0.0192800000
H	-4.3185060000	3.8306670000	-1.6304780000
H	-1.1060960000	3.3894760000	0.4330920000
H	-5.1039670000	1.9158790000	-0.3551510000
H	-1.1455660000	0.6916080000	0.4141820000
H	-0.2760690000	2.5453500000	-0.8958780000
H	-3.5465240000	0.3524010000	0.6910930000
O	-2.6989190000	1.3668620000	-2.8698780000
H	-2.8339900000	0.4698360000	-3.1889020000
H	-2.4282640000	1.8650070000	-3.6482220000
O	-2.1381920000	4.9753680000	1.3521200000
H	-2.2044640000	5.7452710000	0.7597360000
H	-2.0537630000	5.3256350000	2.2424110000
O	-2.7402430000	6.5875730000	-0.8067880000
H	-2.2541210000	6.4398600000	-1.6246070000
H	-3.0399580000	7.5001120000	-0.8396960000

Protonated cyclohexa-2,4-dien-1-one + 4H_2O

Zero-point corrected energy (Eh): -613.31346464

Mode	Frequency (cm^{-1})
6	25.92
7	29.87
8	38.54
9	44.5
10	55.73
11	83.3
12	90.78
13	105.84
14	113.06
15	127.21
16	146.46
17	152.28
18	191.72
19	200.07
20	216.59
21	227.13
22	228.27
23	248.65
24	255.97
25	265.7
26	271.78
27	293.38
28	307.84
29	345.14
30	375.8
31	398.14
32	460.42

33	489.84
34	537.69
35	546.45
36	648.88
37	679.54
38	725.21
39	732.35
40	929.1
41	978.08
42	983.28
43	995.75
44	1027.29
45	1067.74
46	1120.1
47	1169.49
48	1193.19
49	1201.98
50	1216.78
51	1333.17
52	1346.07
53	1373.98
54	1395.51
55	1442.16
56	1572.8
57	1584.89
58	1625.39
59	1644.41
60	1648.62
61	1663.95
62	1866.69

63	2899.15
64	2931.75
65	3144.47
66	3147.29
67	3183.28
68	3186.48
69	3230.49
70	3619.33
71	3806.76
72	3812.14
73	3820.37
74	3887.35
75	3903.97
76	3906.68
77	3908.71

Optimised geometry:

O	-1.2251260000	4.6583310000	-0.9226090000
C	-1.1328280000	2.8002170000	0.5755630000
C	-1.7485320000	3.6648550000	-0.5128350000
C	-1.4726570000	1.3863300000	0.4664760000
C	-2.5872630000	0.9115580000	-0.1820870000
C	-3.3365840000	1.7879460000	-0.9417750000
C	-3.1296840000	3.2263350000	-0.9593200000
H	-2.8015150000	-0.1459810000	-0.1993520000
H	-0.0670690000	2.9814370000	0.6850940000
H	-0.7964950000	0.6742320000	0.9261900000
H	-4.1203060000	1.4131260000	-1.5917470000
H	-1.6328240000	3.1670680000	1.4923950000
H	-3.4484930000	3.6798540000	-1.8940260000
H	-3.7668090000	3.6392940000	-0.1499420000
O	-3.4608650000	4.3163380000	1.8799700000
H	-4.1117170000	4.4463010000	2.5737980000
H	-2.8313050000	5.0555060000	1.9544900000
O	-1.1787360000	5.8645340000	1.7251700000
H	-0.9187230000	6.0826800000	0.8237900000

H	-0.8069530000	6.5514960000	2.2843430000
O	-5.2865140000	2.4633380000	-3.1906950000
H	-6.2005840000	2.7495280000	-3.1068920000
H	-5.1702050000	2.3034260000	-4.1315020000
O	-0.5501900000	2.0828690000	-2.3338840000
H	-0.2950340000	1.3478490000	-2.8984440000
H	-0.0998690000	2.8482590000	-2.7055720000

Protonated cyclohexa-2,4-dien-1-one + 10H_2O

Zero-point corrected energy (Eh): -1071.85486236

Mode	Frequency (cm^{-1})
6	13.24
7	27.37
8	29.36
9	33.42
10	41.99
11	45.39
12	50.3
13	55.56
14	57.94
15	66.41
16	69.24
17	78.64
18	84.71
19	91.71
20	105.53
21	112.49
22	122.09
23	134.04
24	140.78
25	149.04

26	151.3
27	159.88
28	174.09
29	183.86
30	189.75
31	221.97
32	231.01
33	238.54
34	248.05
35	250.48
36	252.68
37	255.53
38	277.53
39	286.14
40	296.28
41	305.73
42	314.1
43	317.8
44	320.86
45	327.17
46	337.44
47	345.88
48	363.98
49	400.8
50	413.87
51	420.76
52	424.99
53	432.08
54	452.56
55	475.68

56	488.98
57	511.31
58	526.25
59	542.79
60	551.15
61	562.7
62	628.98
63	661.82
64	682.36
65	698.69
66	714.82
67	726.93
68	740.1
69	751.78
70	769.93
71	798.87
72	842.86
73	887.69
74	911.22
75	930.02
76	950.6
77	965.93
78	972.37
79	988.51
80	1032.78
81	1066.73
82	1141.99
83	1164.35
84	1198.29
85	1212.86

86	1240.15
87	1347.56
88	1360.43
89	1366.55
90	1402.26
91	1445.68
92	1573.08
93	1588.38
94	1606.34
95	1619.9
96	1632.3
97	1639.1
98	1650.08
99	1657.73
100	1665.47
101	1677.82
102	1679.65
103	1699.07
104	1823.17
105	2766.28
106	2977.6
107	3059.98
108	3126.81
109	3188.05
110	3190.97
111	3231.62
112	3295.97
113	3393.82
114	3446.93
115	3482.1

116	3530.51
117	3550.49
118	3614.42
119	3637.84
120	3647.23
121	3686.12
122	3772.29
123	3811.96
124	3823.11
125	3879.72
126	3880.08
127	3883.04
128	3885.51
129	3895.52
130	3900.7
131	3910.49

Optimised geometry:

O	-1.8732650000	3.9251120000	-2.3500610000
C	-3.2717760000	2.3414610000	-1.2630350000
C	-3.1206460000	1.1282120000	-0.4756650000
C	-2.0391750000	0.8650120000	0.3424240000
C	-1.0111010000	1.7739330000	0.3968450000
C	-2.0317430000	3.1776980000	-1.4154770000
C	-1.0112420000	3.0521670000	-0.3129860000
H	-3.9980810000	2.9534400000	-0.6713370000
H	-3.7872030000	2.1607150000	-2.2104320000
H	-1.3263350000	3.7922400000	0.4435990000
H	-3.9407100000	0.4204620000	-0.5246480000
H	-0.1286010000	1.5708470000	0.9933040000
H	-0.0083980000	3.3539410000	-0.6111700000
H	-1.9967190000	-0.0481700000	0.9155050000
O	-5.2765110000	2.1259220000	-3.8318830000
H	-5.1898050000	1.9309030000	-4.7680740000
H	-5.1407600000	3.0958410000	-3.7325350000

O	-2.9503290000	5.5649590000	0.1955150000
H	-2.2098680000	6.0394490000	-0.2177060000
H	-3.7815260000	5.8906300000	-0.1874000000
O	-0.5957460000	6.1556100000	-1.1097380000
H	-0.1180210000	6.9421420000	-1.3842970000
H	-0.7538960000	5.6359360000	-1.9079350000
O	-3.1406030000	3.7221600000	2.1092400000
H	-3.0948180000	3.9504650000	3.0411170000
H	-3.0412740000	4.5599560000	1.6091940000
O	1.9345410000	2.5086970000	0.7313630000
H	2.3292120000	3.1523260000	1.3265670000
H	2.6820950000	2.0220850000	0.3726510000
O	-4.6265440000	4.6717390000	-3.2944390000
H	-3.6727800000	4.6720630000	-3.1528940000
H	-5.0121900000	5.1217360000	-2.5235430000
O	-6.1056980000	0.8702750000	0.1041570000
H	-6.5884520000	0.8926460000	-0.7589850000
H	-6.5982580000	0.2669680000	0.6661050000
O	-5.4906260000	3.4829740000	0.6206120000
H	-5.8697400000	2.5839970000	0.5720230000
H	-4.8480030000	3.4864160000	1.3504040000
O	-7.2125360000	0.9606110000	-2.3118330000
H	-8.0845600000	1.3180890000	-2.4915410000
H	-6.6012410000	1.3872950000	-2.9453020000
O	-5.5211110000	5.7330610000	-0.8463700000
H	-6.2183550000	6.3790300000	-0.7099530000
H	-5.7318300000	4.9515570000	-0.2916640000

Ipso-protonated + 1H_2O

Zero-point corrected energy (Eh): -384.04953825

Mode	Frequency (cm^{-1})
6	32.56
7	87.1
8	190.24
9	210.42
10	216.25
11	290.13

12	327.92
13	358.87
14	374.42
15	428.09
16	511.63
17	526.52
18	602.23
19	617.56
20	745.83
21	806.16
22	816.55
23	895.49
24	987.54
25	1030.7
26	1044.9
27	1056.69
28	1078.0
29	1104.46
30	1114.03
31	1195.76
32	1203.47
33	1249.37
34	1300.72
35	1377.42
36	1456.52
37	1494.58
38	1525.16
39	1597.32
40	1650.51
41	1666.14

42	2589.14
43	3203.34
44	3208.6
45	3215.11
46	3228.63
47	3233.05
48	3461.53
49	3807.55
50	3891.0

Optimised geometry:

O	-2.8531350000	4.8903570000	-0.0600710000
C	-2.9633990000	3.5627810000	0.2101160000
C	-1.7554170000	2.7832670000	0.0050370000
C	-1.8165500000	1.4169830000	-0.0749710000
C	-3.0609620000	0.7843360000	-0.0707370000
C	-4.2612190000	1.4939050000	0.0432460000
C	-4.2370590000	2.8597700000	0.1231300000
H	-2.8977560000	3.3384660000	1.3377760000
H	-5.1404910000	3.4503910000	0.2094240000
H	-3.7039820000	5.3504930000	0.1038130000
H	-0.8206380000	3.3279360000	-0.0036900000
H	-0.9117160000	0.8360500000	-0.1691650000
H	-3.0986830000	-0.2938560000	-0.1513380000
H	-5.2029760000	0.9661320000	0.0409840000
O	-5.3302070000	5.9219310000	0.3571600000
H	-5.5311040000	6.4577320000	1.1313410000
H	-5.7279800000	6.3892050000	-0.3849680000

Meta-protonated + 1H_2O

Zero-point corrected energy (Eh): -384.06880316

Mode	Frequency (cm^{-1})
6	45.04
7	90.45

8	171.57
9	190.85
10	213.37
11	215.26
12	300.66
13	364.72
14	392.81
15	440.77
16	507.83
17	513.93
18	602.19
19	679.2
20	811.28
21	818.41
22	865.42
23	922.2
24	956.63
25	994.57
26	1032.53
27	1043.85
28	1072.02
29	1133.65
30	1148.23
31	1203.31
32	1260.16
33	1285.73
34	1335.18
35	1363.6
36	1442.37
37	1482.82

38	1509.77
39	1627.69
40	1647.68
41	1673.34
42	2970.98
43	2975.81
44	3184.69
45	3212.87
46	3216.72
47	3232.42
48	3411.9
49	3807.13
50	3893.05

Optimised geometry:

O	0.2560930000	2.9470380000	-0.0031410000
C	1.3253270000	0.8732370000	-0.0541570000
C	1.2837760000	-0.5823540000	-0.0717820000
C	-0.0218910000	-1.2266080000	-0.0502420000
C	-1.1542640000	-0.4730580000	-0.0147540000
C	-1.0534210000	0.9252180000	0.0008920000
C	0.1743070000	1.6201030000	-0.0182720000
H	1.8589460000	-0.9574570000	-0.9372220000
H	-0.6300150000	3.3721790000	0.0232020000
H	-0.0654140000	-2.3065850000	-0.0630720000
H	-2.1312860000	-0.9318460000	0.0016590000
H	-1.9621540000	1.5166400000	0.0292630000
H	2.2836880000	1.3746880000	-0.0696120000
H	1.8974020000	-0.9789300000	0.7568860000
O	-2.2459960000	3.9587660000	0.0689400000
H	-2.5173150000	4.4549450000	0.8481310000
H	-2.5537470000	4.4742130000	-0.6837570000

Meta-protonated + $2H_2O$

Zero-point corrected energy (Eh): -460.49082704

Mode	Frequency (cm^{-1})
6	7.47
7	35.6
8	41.52
9	54.66
10	88.96
11	164.18
12	181.01
13	209.56
14	215.42
15	229.82
16	269.49
17	293.21
18	349.92
19	379.01
20	439.74
21	448.7
22	517.72
23	546.29
24	609.2
25	667.39
26	810.16
27	836.85
28	849.73
29	896.81
30	976.16
31	1000.41
32	1013.18
33	1039.39
34	1066.28

35	1097.16
36	1164.72
37	1202.93
38	1256.66
39	1285.09
40	1334.65
41	1375.3
42	1442.52
43	1485.68
44	1513.5
45	1625.05
46	1638.79
47	1646.34
48	1668.73
49	2476.5
50	3095.04
51	3189.23
52	3214.91
53	3218.07
54	3232.2
55	3449.88
56	3810.39
57	3817.82
58	3896.97
59	3908.99

Optimised geometry:

O	0.2656160000	2.9489960000	0.1120010000
C	1.3679140000	0.8939430000	0.2446290000
C	1.3581140000	-0.5475020000	0.1737320000
C	0.0869860000	-1.2222450000	0.0747900000
C	-1.0594150000	-0.4922700000	-0.0703170000

C	-0.9964610000	0.9031460000	-0.0592760000
C	0.2069670000	1.6173670000	0.0939870000
H	1.7888060000	-0.7643940000	-0.8672430000
H	-0.6252800000	3.3543810000	0.0412620000
H	0.0702520000	-2.3024430000	0.0578750000
H	-2.0162800000	-0.9781420000	-0.1878610000
H	-1.9104770000	1.4745550000	-0.1737010000
H	2.3058260000	1.4173330000	0.3704620000
H	2.1087380000	-1.0455810000	0.7902720000
O	-2.2573560000	3.9278210000	-0.0877830000
H	-2.6327520000	4.3809990000	0.6739150000
H	-2.4849560000	4.4722990000	-0.8480780000
O	2.4149260000	-1.3291340000	-2.4964160000
H	3.1829940000	-1.8965210000	-2.6110190000
H	2.3145500000	-0.8635150000	-3.3316850000

Meta-protonated + 3H_2O

Zero-point corrected energy (Eh): -536.90936424

Mode	Frequency (cm^{-1})
6	-38.39
7	-20.76
8	-10.46
9	14.8
10	45.78
11	55.09
12	58.33
13	88.22
14	136.77
15	157.75
16	160.48
17	176.76
18	180.95
19	206.09
20	212.05

21	219.55
22	280.11
23	283.62
24	340.33
25	371.01
26	436.98
27	443.95
28	519.02
29	532.44
30	609.52
31	691.29
32	821.56
33	826.26
34	835.89
35	922.57
36	978.21
37	999.66
38	1024.63
39	1052.25
40	1065.1
41	1141.06
42	1146.59
43	1197.34
44	1225.15
45	1283.2
46	1329.41
47	1365.82
48	1453.09
49	1485.55
50	1505.19

51	1630.09
52	1639.88
53	1642.99
54	1645.59
55	1672.34
56	2771.08
57	2802.7
58	3187.5
59	3212.76
60	3215.14
61	3230.41
62	3478.2
63	3812.41
64	3825.66
65	3826.71
66	3899.88
67	3916.64
68	3917.02

Optimised geometry:

O	0.1646800000	3.0108010000	0.0565750000
C	1.2427390000	0.9371050000	-0.0505840000
C	1.2074740000	-0.5028220000	-0.1110530000
C	-0.0779220000	-1.1539610000	-0.1140600000
C	-1.2233170000	-0.4119400000	-0.0604640000
C	-1.1370590000	0.9827350000	-0.0032300000
C	0.0853050000	1.6783390000	0.0022650000
H	1.8188320000	-0.8692700000	-0.9722880000
H	-0.7225990000	3.4246080000	0.0942530000
H	-0.1111130000	-2.2331860000	-0.1603040000
H	-2.1955040000	-0.8814130000	-0.0621960000
H	-2.0493710000	1.5668100000	0.0392240000
H	2.1977990000	1.4446710000	-0.0458870000
H	1.8561120000	-0.9360970000	0.6896440000

O	-2.3629650000	4.0254120000	0.1585610000
H	-2.6337360000	4.4995550000	0.9509970000
H	-2.6726800000	4.5605240000	-0.5788730000
O	2.8413660000	-1.5043640000	-2.5129340000
H	3.4901130000	-2.2125070000	-2.5454130000
H	2.7992210000	-1.1621350000	-3.4099740000
O	2.9780830000	-1.6742720000	2.1093670000
H	3.8869660000	-1.9792330000	2.0432170000
H	2.7702380000	-1.7089670000	3.0470130000

Meta-protonated + 4H_2O

Zero-point corrected energy (Eh): -613.32791900

Mode	Frequency (cm^{-1})
6	-18.84
7	-16.02
8	17.85
9	32.3
10	40.17
11	47.15
12	58.84
13	92.56
14	128.75
15	157.49
16	168.44
17	175.66
18	182.37
19	194.01
20	198.35
21	205.49
22	207.63
23	220.9
24	225.12

25	255.38
26	268.78
27	285.75
28	329.49
29	333.22
30	404.17
31	435.23
32	451.02
33	517.24
34	531.33
35	609.15
36	642.6
37	681.84
38	806.1
39	824.35
40	834.43
41	912.56
42	973.44
43	990.27
44	1012.64
45	1055.61
46	1085.48
47	1114.5
48	1160.57
49	1185.73
50	1203.72
51	1281.63
52	1327.11
53	1365.15
54	1445.42

55	1481.89
56	1505.92
57	1619.81
58	1624.11
59	1644.37
60	1648.03
61	1653.61
62	1672.96
63	2857.44
64	2974.14
65	3186.46
66	3217.3
67	3222.89
68	3235.51
69	3492.55
70	3696.63
71	3814.07
72	3815.55
73	3823.65
74	3897.49
75	3901.85
76	3904.57
77	3914.05

Optimised geometry:

O	-0.0598680000	2.8523590000	0.0118250000
C	1.1732220000	0.8992750000	-0.3575000000
C	1.2570470000	-0.5392730000	-0.4657550000
C	0.0347890000	-1.3032150000	-0.4163610000
C	-1.1546610000	-0.6719710000	-0.1807170000
C	-1.1795930000	0.7168600000	-0.0362580000
C	-0.0277810000	1.5223160000	-0.1222160000

H	1.9814000000	-0.8843290000	-1.2251990000
H	-0.9703820000	3.1765110000	0.1683070000
H	0.0580930000	-2.3780270000	-0.5264470000
H	-2.0642660000	-1.2486420000	-0.1121330000
H	-2.1253460000	1.2112420000	0.1550470000
H	2.0789550000	1.4875280000	-0.4066630000
H	1.8536490000	-0.8418240000	0.4134660000
O	-2.6392320000	3.6280530000	0.4579250000
H	-2.8450640000	4.0330990000	1.3061430000
H	-3.0740980000	4.1776660000	-0.2013780000
O	4.4705430000	-1.5470370000	-1.2194060000
H	4.7119980000	-2.4297410000	-1.5155730000
H	5.1314800000	-0.9662380000	-1.6085490000
O	3.4892740000	-0.9181640000	1.3351030000
H	4.0739110000	-1.2400960000	0.6323680000
H	3.8364210000	-1.2515240000	2.1653920000
O	-1.9238790000	-3.7078420000	-0.3995080000
H	-2.1276700000	-4.2898370000	0.3378420000
H	-2.3306490000	-4.1317780000	-1.1602660000

O-protonated + 1H_2O

Zero-point corrected energy (Eh): -384.08657666

Mode	Frequency (cm^{-1})
6	23.11
7	55.73
8	98.57
9	224.78
10	335.87
11	364.12
12	407.14
13	420.56
14	440.21
15	489.39
16	517.68
17	538.99

18	620.96
19	702.85
20	774.47
21	795.78
22	845.88
23	945.76
24	1013.46
25	1021.11
26	1050.31
27	1050.92
28	1062.41
29	1105.92
30	1150.46
31	1188.24
32	1195.09
33	1326.19
34	1343.91
35	1359.39
36	1506.37
37	1526.8
38	1614.61
39	1646.58
40	1660.52
41	1699.31
42	2057.52
43	3198.25
44	3212.52
45	3220.41
46	3226.94
47	3233.89

48	3755.8
49	3791.35
50	3878.63

Optimised geometry:

C	-4.2522770000	2.8341680000	-0.0100210000
C	-4.2911640000	1.4501170000	0.0913220000
C	-3.1133090000	0.7239330000	0.1978700000
C	-1.8825810000	1.3659980000	0.2022740000
C	-3.0121810000	3.4258590000	-0.0067060000
H	-5.1504720000	3.4295540000	-0.0798720000
H	-5.2449560000	0.9440460000	0.0946410000
H	-0.9699540000	0.7956720000	0.2899880000
H	-0.8695910000	3.2683920000	0.0964610000
O	-2.9893020000	4.8777970000	-0.1337060000
H	-2.4714690000	5.3000110000	0.5674550000
H	-3.1542630000	-0.3519180000	0.2823370000
C	-1.8180530000	2.7482470000	0.0939620000
H	-2.7146800000	5.2186130000	-1.1108150000
O	-2.3706650000	5.6727550000	-2.3689430000
H	-1.9711020000	5.0771370000	-3.0126730000
H	-2.9761410000	6.2538680000	-2.8432720000

O-protonated + 2H_2O

Zero-point corrected energy (Eh): -460.52571023

Mode	Frequency (cm^{-1})
6	11.18
7	53.13
8	58.36
9	65.19
10	83.05
11	124.26
12	221.33
13	264.1

14	325.77
15	332.26
16	358.34
17	400.61
18	423.74
19	445.45
20	447.5
21	523.51
22	541.6
23	624.15
24	709.13
25	781.62
26	807.48
27	853.13
28	907.6
29	949.84
30	1013.55
31	1025.16
32	1047.42
33	1053.66
34	1105.65
35	1125.49
36	1178.34
37	1186.41
38	1205.95
39	1320.22
40	1343.5
41	1391.8
42	1508.09
43	1532.15

44	1637.07
45	1643.29
46	1662.56
47	1682.74
48	1729.92
49	2626.2
50	2778.84
51	3203.99
52	3210.44
53	3217.21
54	3223.69
55	3231.42
56	3805.12
57	3806.51
58	3892.32
59	3894.77

Optimised geometry:

C	-4.2511780000	2.8481020000	-0.1175620000
C	-4.3036610000	1.4617820000	-0.0858910000
C	-3.1456160000	0.7234070000	0.1142710000
C	-1.9240110000	1.3599460000	0.2810300000
C	-3.0202580000	3.4419640000	0.0435990000
H	-5.1378240000	3.4497110000	-0.2539550000
H	-5.2527750000	0.9617640000	-0.2096960000
H	-1.0259270000	0.7813810000	0.4391420000
H	-0.9070690000	3.2629840000	0.3696590000
O	-2.9803520000	4.8822080000	0.0003540000
H	-2.3351560000	5.2745990000	0.6882300000
H	-3.1959660000	-0.3550040000	0.1437950000
C	-1.8475640000	2.7455080000	0.2412340000
H	-2.8176410000	5.2347270000	-0.9511300000
O	-2.5695750000	5.7507110000	-2.3260950000
H	-2.1370420000	5.2036390000	-2.9897560000
H	-3.2449930000	6.2628200000	-2.7826830000

O	-1.3456760000	5.7376860000	1.7444860000
H	-1.5286550000	5.5610450000	2.6733440000
H	-0.9143630000	6.5974460000	1.7063720000

O-protonated + 3H_2O

Zero-point corrected energy (Eh): -536.95527916

Mode	Frequency (cm^{-1})
6	16.92
7	26.84
8	44.41
9	64.79
10	76.43
11	88.53
12	117.0
13	125.8
14	224.47
15	242.16
16	254.53
17	269.73
18	291.84
19	318.92
20	367.41
21	389.34
22	393.58
23	422.14
24	471.92
25	513.94
26	543.5
27	614.31
28	628.35

29	712.82
30	780.84
31	810.09
32	831.14
33	858.41
34	946.54
35	984.49
36	1011.17
37	1024.91
38	1040.58
39	1059.8
40	1112.56
41	1182.3
42	1185.55
43	1207.38
44	1234.45
45	1327.52
46	1357.75
47	1395.91
48	1492.38
49	1526.53
50	1565.46
51	1627.4
52	1643.13
53	1653.15
54	1675.44
55	1699.07
56	1816.29
57	3022.98
58	3199.43

59	3206.43
60	3213.46
61	3219.56
62	3229.87
63	3312.28
64	3813.74
65	3814.34
66	3862.5
67	3901.93
68	3902.26

Optimised geometry:

C	-4.1384030000	2.8273090000	0.0748500000
C	-4.1780350000	1.4904030000	0.4428840000
C	-3.0628200000	0.6827450000	0.2715450000
C	-1.8974030000	1.2085480000	-0.2652340000
C	-2.9599040000	3.3181670000	-0.4465190000
H	-5.0019280000	3.4699960000	0.1795640000
H	-5.0883870000	1.0779750000	0.8527080000
H	-1.0299220000	0.5797020000	-0.4015510000
H	-0.9298590000	2.9730050000	-1.0433930000
O	-2.9429540000	4.6931440000	-0.8117750000
H	-3.2057760000	5.3660760000	0.0168330000
H	-3.1037650000	-0.3590630000	0.5527480000
C	-1.8317250000	2.5472050000	-0.6271730000
H	-2.1446960000	4.9640140000	-1.3595850000
O	-0.9151520000	5.3245680000	-2.2812250000
H	-1.0041060000	5.3077000000	-3.2393660000
H	-0.2457200000	5.9848390000	-2.0783620000
O	-3.6088900000	6.1733210000	0.9850100000
H	-3.1330120000	6.1299450000	1.8200080000
H	-4.5769560000	6.0855660000	1.1727280000
O	-6.2148220000	5.7593950000	1.2461320000
H	-6.6644460000	5.4994130000	2.0558820000
H	-6.8312900000	6.3293800000	0.7759180000

Ortho-protonated + 1H_2O

Zero-point corrected energy (Eh): -384.09442346

Mode	Frequency (cm^{-1})
6	-22.73
7	84.08
8	118.45
9	158.77
10	244.97
11	257.09
12	379.37
13	383.17
14	446.8
15	473.14
16	501.12
17	506.66
18	599.59
19	743.53
20	805.37
21	842.21
22	935.29
23	950.85
24	985.42
25	1032.48
26	1035.64
27	1067.94
28	1098.02
29	1193.38
30	1200.6
31	1202.31
32	1262.39

33	1341.69
34	1358.19
35	1402.52
36	1466.19
37	1514.12
38	1549.16
39	1609.48
40	1651.99
41	1717.63
42	3025.85
43	3043.81
44	3055.71
45	3202.18
46	3214.74
47	3233.62
48	3238.42
49	3801.59
50	3885.08

Optimised geometry:

C	-4.3095520000	2.8110100000	-0.0496150000
C	-4.2325240000	1.3303810000	-0.0417150000
C	-3.0562330000	0.6926460000	-0.0028160000
C	-1.8396690000	1.4554240000	0.0285540000
C	-1.8020500000	2.8187650000	0.0178520000
C	-3.0116660000	3.5277920000	-0.0273650000
H	-4.9038820000	3.1526670000	0.8074810000
H	-5.1670630000	0.7879330000	-0.0649350000
H	-3.0015670000	-0.3848570000	0.0054490000
H	-0.9019740000	0.9157950000	0.0611520000
H	-0.8745550000	3.3702940000	0.0389870000
O	-2.9702440000	4.8132930000	-0.0451970000
H	-3.8702230000	5.2565860000	-0.0866910000
H	-4.8884430000	3.1551820000	-0.9146820000

O	-5.3170590000	5.9364760000	-0.1811250000
H	-5.5227240000	6.4537240000	-0.9673180000
H	-5.6281620000	6.4604330000	0.5648910000

Ortho-protonated + 2H_2O

Zero-point corrected energy (Eh): -460.52107665

Mode	Frequency (cm^{-1})
6	33.37
7	42.75
8	77.56
9	99.58
10	150.5
11	225.74
12	232.05
13	270.53
14	282.67
15	291.5
16	326.71
17	376.79
18	450.08
19	474.31
20	506.5
21	516.2
22	544.56
23	601.61
24	745.5
25	773.39
26	803.45
27	842.11
28	936.69

29	953.03
30	987.38
31	1033.49
32	1034.86
33	1066.65
34	1127.51
35	1193.59
36	1201.6
37	1214.57
38	1286.42
39	1353.56
40	1370.65
41	1420.56
42	1475.68
43	1551.06
44	1558.91
45	1616.64
46	1627.16
47	1659.3
48	1720.24
49	2724.95
50	3007.25
51	3045.61
52	3202.07
53	3212.84
54	3233.23
55	3237.33
56	3543.08
57	3806.36
58	3873.05

59	3892.91

Optimised geometry:

C	-4.1849720000	2.6785110000	-0.0115640000
C	-4.0724280000	1.2045090000	-0.1178850000
C	-2.8832580000	0.5982360000	-0.2110110000
C	-1.6825390000	1.3897440000	-0.2143900000
C	-1.6768910000	2.7482210000	-0.1314020000
C	-2.9057240000	3.4292020000	-0.0406230000
H	-4.7168180000	2.9461710000	0.9131340000
H	-4.9923320000	0.6370930000	-0.1134390000
H	-2.8043510000	-0.4752510000	-0.2857040000
H	-0.7341070000	0.8739480000	-0.2905790000
H	-0.7639170000	3.3237380000	-0.1425230000
O	-2.8985200000	4.7084020000	0.0212520000
H	-3.8329370000	5.1195850000	0.0616200000
H	-4.8375500000	3.0737220000	-0.7997260000
O	-5.2724760000	5.6058840000	0.0846790000
H	-5.4753670000	6.5152030000	-0.1517850000
H	-5.7304140000	5.3954950000	0.9219820000
O	-6.2013380000	4.4270290000	2.3441950000
H	-7.1224250000	4.1583490000	2.4224790000
H	-5.9460540000	4.7128420000	3.2272460000

Ortho-protonated + 3H_2O

Zero-point corrected energy (Eh): -536.94319293

Mode	Frequency (cm^{-1})
6	-17.3
7	55.86
8	66.36
9	81.18
10	105.17
11	116.89
12	147.9
13	199.34

14	235.51
15	260.84
16	273.5
17	297.0
18	313.73
19	336.67
20	364.11
21	396.53
22	438.98
23	454.85
24	477.68
25	509.58
26	511.59
27	559.33
28	601.14
29	657.71
30	748.98
31	808.65
32	840.35
33	865.87
34	937.09
35	954.72
36	990.75
37	1029.82
38	1035.84
39	1048.73
40	1068.08
41	1192.57
42	1199.04
43	1213.7

44	1288.69
45	1338.71
46	1373.88
47	1419.33
48	1476.59
49	1543.2
50	1556.96
51	1612.28
52	1618.36
53	1628.84
54	1661.37
55	1718.63
56	2853.05
57	2993.71
58	3036.25
59	3200.83
60	3211.48
61	3232.48
62	3236.28
63	3407.52
64	3705.3
65	3789.02
66	3867.49
67	3886.22
68	3894.11

Optimised geometry:

C	-3.9656410000	2.6071740000	0.3405700000
C	-3.8378800000	1.1366730000	0.4390430000
C	-2.7120750000	0.5058360000	0.0811780000
C	-1.5982740000	1.2721850000	-0.4035630000
C	-1.6146360000	2.6285640000	-0.5287520000

C	-2.7797160000	3.3286420000	-0.1669820000
H	-4.2461320000	3.0244870000	1.3164860000
H	-4.6939650000	0.5926670000	0.8123110000
H	-2.6212940000	-0.5669850000	0.1516620000
H	-0.6982580000	0.7402870000	-0.6837650000
H	-0.7652390000	3.1842840000	-0.8955270000
O	-2.7918630000	4.6060610000	-0.2919250000
H	-3.6653110000	5.0480090000	-0.0268500000
H	-4.8296370000	2.8730820000	-0.2873060000
O	-5.0055730000	5.7863720000	0.2549530000
H	-4.9594630000	6.6983320000	0.5559550000
H	-5.6884440000	5.3207890000	0.7899120000
O	-6.7723540000	4.0503740000	1.2275460000
H	-7.0969950000	3.8407610000	0.3382440000
H	-7.5396700000	4.1241730000	1.8011330000
O	-6.3527560000	3.9179480000	-1.5045390000
H	-6.7584970000	3.6956820000	-2.3473780000
H	-6.0801610000	4.8406820000	-1.5716710000

Ortho-protonated + $4H_2O$

Zero-point corrected energy (Eh): -613.36779865

Mode	Frequency (cm^{-1})
6	22.1
7	37.99
8	50.8
9	63.16
10	68.99
11	80.36
12	90.16
13	111.31
14	166.51
15	192.77
16	198.79
17	247.03

18	263.74
19	269.49
20	281.63
21	297.66
22	298.41
23	317.41
24	320.31
25	334.22
26	438.76
27	446.2
28	457.5
29	490.43
30	509.44
31	525.33
32	598.64
33	624.34
34	677.95
35	749.79
36	766.17
37	805.83
38	840.59
39	886.77
40	937.74
41	955.33
42	987.12
43	1036.32
44	1056.34
45	1065.41
46	1113.38
47	1192.01

48	1205.63
49	1212.49
50	1291.52
51	1342.34
52	1375.64
53	1426.38
54	1479.23
55	1559.99
56	1571.17
57	1618.21
58	1631.57
59	1642.09
60	1658.25
61	1673.3
62	1719.99
63	2588.97
64	2998.18
65	3046.53
66	3198.69
67	3204.49
68	3230.38
69	3234.33
70	3393.39
71	3519.35
72	3618.67
73	3815.79
74	3881.64
75	3882.11
76	3889.09
77	3904.33

Optimised geometry:

C	-4.0929100000	2.7148270000	0.2208020000
C	-3.9532320000	1.2615070000	-0.0219040000
C	-2.8078080000	0.7382190000	-0.4775490000
C	-1.6907690000	1.6018330000	-0.7485520000
C	-1.7242740000	2.9502140000	-0.5743650000
C	-2.9132690000	3.5471990000	-0.1061720000
H	-4.3444670000	2.8858380000	1.2786850000
H	-4.8131640000	0.6412240000	0.1932030000
H	-2.7026340000	-0.3221920000	-0.6477770000
H	-0.7765250000	1.1521640000	-1.1138610000
H	-0.8765930000	3.5822790000	-0.7905470000
O	-2.9469220000	4.8169560000	0.0422020000
H	-3.8581730000	5.1722830000	0.3626080000
H	-4.9803040000	3.1059650000	-0.2916250000
O	-5.2310390000	5.6101630000	0.7560510000
H	-5.4088360000	6.5470440000	0.8707040000
H	-5.6120180000	5.1229490000	1.5221920000
O	-6.0562920000	3.8177770000	2.5286370000
H	-6.6604320000	3.2427850000	2.0165820000
H	-6.3957160000	3.8758210000	3.4245930000
O	-6.9500010000	-0.4830440000	0.7088050000
H	-7.3003120000	-1.0330080000	0.0014150000
H	-7.0565360000	-1.0040250000	1.5105180000
O	-7.2797340000	2.2802150000	0.6670140000
H	-7.3139670000	1.3086620000	0.7183530000
H	-8.0955920000	2.5575300000	0.2424780000

Ortho-protonated + 10H_2O

Zero-point corrected energy (Eh): -1071.89671717

Mode	Frequency (cm^{-1})
6	18.65
7	21.79
8	23.64
9	30.95
10	37.85
11	41.09

12	44.29
13	50.03
14	54.58
15	60.81
16	63.55
17	71.25
18	81.15
19	82.78
20	89.08
21	92.54
22	104.46
23	107.69
24	120.95
25	156.36
26	181.78
27	187.21
28	189.28
29	195.3
30	200.9
31	217.58
32	222.8
33	232.25
34	233.55
35	242.21
36	246.57
37	253.78
38	260.83
39	268.43
40	276.97
41	285.83

42	290.87
43	295.62
44	302.03
45	305.08
46	311.01
47	339.37
48	395.23
49	396.53
50	404.14
51	417.15
52	434.71
53	444.14
54	464.48
55	470.91
56	481.24
57	499.17
58	509.17
59	526.12
60	557.22
61	596.89
62	613.97
63	625.54
64	665.94
65	688.53
66	709.78
67	745.67
68	755.67
69	785.38
70	803.1
71	814.45

72	844.49
73	869.05
74	891.7
75	931.49
76	961.09
77	992.11
78	1015.91
79	1032.98
80	1039.6
81	1076.06
82	1105.95
83	1187.67
84	1201.05
85	1208.94
86	1289.01
87	1346.23
88	1371.3
89	1418.68
90	1476.7
91	1545.66
92	1550.19
93	1620.09
94	1621.57
95	1628.14
96	1629.75
97	1642.97
98	1649.82
99	1655.33
100	1664.38
101	1667.01

102	1679.67
103	1699.76
104	1714.85
105	2781.65
106	2963.88
107	2985.16
108	3071.92
109	3194.39
110	3219.86
111	3233.48
112	3236.81
113	3428.4
114	3455.92
115	3526.01
116	3543.24
117	3579.58
118	3598.43
119	3639.65
120	3650.37
121	3684.11
122	3819.76
123	3871.27
124	3880.92
125	3889.52
126	3889.94
127	3890.27
128	3893.39
129	3894.59
130	3909.06
131	3909.15

Optimised geometry:

C	-4.2173900000	2.8966330000	-0.3187730000
C	-3.9393360000	1.4780820000	-0.6168130000
C	-2.7064850000	1.0641710000	-0.9424750000
C	-1.6295310000	2.0117680000	-0.9774580000
C	-1.7785760000	3.3317270000	-0.6754070000
C	-3.0484450000	3.7952240000	-0.2924410000
H	-4.7984400000	2.9934710000	0.6034600000
H	-4.7854160000	0.8082360000	-0.5810860000
H	-2.5051610000	0.0303140000	-1.1776840000
H	-0.6443410000	1.6580670000	-1.2556340000
H	-0.9387690000	4.0095060000	-0.6672770000
O	-3.1832610000	5.0329420000	0.0341800000
H	-4.1340050000	5.2857540000	0.2700720000
H	-4.8956330000	3.2855720000	-1.0961900000
O	-5.6380720000	5.6944620000	0.5020920000
H	-5.8211350000	6.5399180000	0.9204850000
H	-6.1790790000	4.9885230000	0.9883150000
O	-7.0125790000	3.7919690000	1.4821930000
H	-7.2804720000	3.1830760000	0.7711200000
H	-6.9174940000	3.2528590000	2.2961190000
O	-6.3885340000	4.6623300000	-2.0095390000
H	-6.5756060000	5.1804620000	-2.7957120000
H	-6.2870430000	5.2892310000	-1.2755890000
O	-7.3099540000	2.3191260000	-0.8519860000
H	-7.2325070000	3.0810170000	-1.4510580000
H	-8.1057670000	1.8472530000	-1.1087370000
O	-4.4908240000	0.8157100000	2.3851480000
H	-3.7197060000	1.4130670000	2.4571070000
H	-4.1733040000	-0.0670600000	2.5873000000
O	1.7220690000	1.6464500000	-2.1206630000
H	1.8358120000	1.9555240000	-3.0241880000
H	2.2528160000	0.8471170000	-2.0561400000
O	-2.5258680000	2.7163330000	2.3714260000
H	-2.4682530000	3.3146920000	3.1202970000
H	-1.6092650000	2.4105240000	2.1965810000
O	-0.0623050000	1.7778760000	1.7284510000
H	0.4920100000	1.3124330000	2.3588000000
H	0.5293860000	2.3715250000	1.2260350000
O	-6.6711950000	2.0455060000	3.4853790000
H	-5.9045770000	1.4968620000	3.2343410000
H	-6.6782820000	2.1096810000	4.4415830000

O	1.3962190000	3.3709430000	0.0346050000
H	2.1072530000	3.9809440000	0.2437440000
H	1.6915190000	2.8439130000	-0.7272660000

Proton transfer + 1H_2O

Zero-point corrected energy (Eh): -384.06694703

Mode	Frequency (cm^{-1})
6	31.74
7	66.86
8	105.8
9	205.45
10	289.94
11	348.64
12	397.31
13	412.59
14	422.58
15	490.61
16	532.6
17	544.72
18	621.92
19	721.75
20	789.65
21	837.39
22	852.99
23	918.87
24	1007.61
25	1022.12
26	1047.44
27	1051.86
28	1052.87

29	1097.27
30	1176.1
31	1192.83
32	1197.32
33	1306.05
34	1349.05
35	1370.17
36	1491.01
37	1537.9
38	1548.82
39	1616.54
40	1646.13
41	1674.67
42	2049.11
43	3173.61
44	3205.34
45	3210.15
46	3222.16
47	3231.55
48	3694.72
49	3778.05
50	3819.15

Optimised geometry:

C	-0.8095600000	8.6951530000	-1.7454150000
C	-0.5422270000	7.6712320000	-2.6346270000
C	-0.4505150000	6.3447510000	-2.2094430000
C	-0.6424740000	6.0495980000	-0.8760480000
C	-0.9573060000	7.0696250000	0.0412180000
C	-0.9878220000	8.4040670000	-0.3926880000
O	-1.2589600000	9.4339700000	0.4363340000
H	-0.8481390000	9.7265940000	-2.0644070000
H	-0.3879390000	7.9092190000	-3.6775290000

H	-0.2185840000	5.5611740000	-2.9144030000
H	-0.5638330000	5.0315770000	-0.5209200000
H	-1.0499250000	6.8431780000	1.0971880000
H	-1.1183850000	9.1858120000	1.3566180000
O	-3.5351250000	7.0286640000	-1.0341050000
H	-2.5472450000	7.0347860000	-0.6197790000
H	-4.1002430000	6.3371260000	-0.6525440000
H	-3.9609330000	7.8989840000	-0.9608890000

Proton transfer + $2H_2O$

Zero-point corrected energy (Eh): -460.52477198

Mode	Frequency (cm^{-1})
6	51.54
7	56.02
8	86.75
9	138.72
10	202.67
11	230.15
12	304.01
13	356.61
14	381.51
15	398.72
16	424.21
17	428.76
18	484.73
19	515.26
20	534.1
21	547.45
22	620.66
23	626.57
24	717.81

25	795.52
26	822.7
27	863.83
28	943.97
29	1013.88
30	1017.96
31	1024.69
32	1042.05
33	1052.79
34	1104.94
35	1177.2
36	1190.54
37	1206.54
38	1242.45
39	1295.62
40	1330.82
41	1361.54
42	1507.43
43	1530.31
44	1576.57
45	1601.86
46	1668.15
47	1681.03
48	1740.96
49	2007.53
50	2808.66
51	3190.92
52	3203.16
53	3210.92
54	3218.43

55	3227.26
56	3691.03
57	3802.19
58	3804.83
59	3855.94

Optimised geometry:

C	-1.1320730000	8.6091170000	-1.8756160000
C	-0.7189150000	7.5143170000	-2.6255760000
C	-0.4038720000	6.3139250000	-1.9983420000
C	-0.4848980000	6.2121130000	-0.6146370000
C	-0.9095600000	7.2935810000	0.1444540000
C	-1.2305640000	8.4741040000	-0.5025700000
O	-1.7821710000	9.5570580000	0.1951550000
H	-1.3637410000	9.5572940000	-2.3400390000
H	-0.6284910000	7.6067190000	-3.6983900000
H	-0.0723280000	5.4691390000	-2.5839410000
H	-0.2213180000	5.2872520000	-0.1225960000
H	-0.9943730000	7.2135730000	1.2204400000
H	-1.4412300000	9.5933840000	1.0967100000
O	-3.8839820000	6.9613010000	-1.7097820000
H	-2.9685660000	6.8109640000	-1.9948900000
H	-4.2520000000	6.1152690000	-1.4330380000
H	-4.0818040000	8.0814430000	-0.9216260000
O	-4.2068590000	8.9626220000	-0.3184880000
H	-3.3020870000	9.3347290000	-0.0328080000
H	-4.7393540000	9.6373620000	-0.7601470000

Proton transfer + 3H_2O

Zero-point corrected energy (Eh): -536.95806085

Mode	Frequency (cm^{-1})
6	31.77
7	39.15
8	63.74
9	69.28

10	82.15
11	107.68
12	165.01
13	189.35
14	256.38
15	285.74
16	297.62
17	330.44
18	365.44
19	372.78
20	397.88
21	415.82
22	426.72
23	490.74
24	530.7
25	532.68
26	536.71
27	626.77
28	634.99
29	718.72
30	745.31
31	804.0
32	833.35
33	869.45
34	939.42
35	998.59
36	1011.8
37	1020.91
38	1034.21
39	1053.02

40	1102.37
41	1173.54
42	1191.86
43	1202.2
44	1272.76
45	1275.97
46	1332.35
47	1364.43
48	1508.44
49	1536.58
50	1616.59
51	1631.55
52	1663.32
53	1674.77
54	1679.31
55	1710.81
56	2298.69
57	2880.23
58	3184.79
59	3200.35
60	3209.82
61	3216.3
62	3225.35
63	3536.01
64	3569.26
65	3811.68
66	3820.05
67	3866.4
68	3899.62

Optimised geometry:

C	-1.0904920000	8.5475110000	-1.9953400000
C	-0.9690940000	7.3047750000	-2.6071560000
C	-0.7605420000	6.1597190000	-1.8437220000
C	-0.6531110000	6.2667500000	-0.4623890000
C	-0.7786350000	7.5009810000	0.1591940000
C	-1.0015610000	8.6320190000	-0.6136160000
O	-1.2259450000	9.8705500000	-0.0489070000
H	-1.2379430000	9.4488750000	-2.5731150000
H	-1.0217970000	7.2351650000	-3.6846220000
H	-0.6547660000	5.1986100000	-2.3248080000
H	-0.4698800000	5.3855810000	0.1353590000
H	-0.7035170000	7.5839370000	1.2360640000
H	-0.8232440000	9.9178820000	0.8250950000
O	-3.8687170000	7.1941380000	-1.3015070000
H	-3.0023430000	7.0801420000	-1.7439560000
H	-4.0250900000	6.4425080000	-0.6372050000
H	-3.9133600000	8.1491820000	-0.8674370000
O	-3.9506850000	9.4416900000	-0.2381310000
H	-3.0770710000	9.8540460000	-0.0972620000
H	-4.5490170000	10.1167070000	-0.5724290000
O	-4.2493680000	5.2323790000	0.2879450000
H	-4.8805300000	4.5515380000	0.0334730000
H	-4.3004170000	5.3090590000	1.2454860000

Proton transfer + $4H_2O$

Zero-point corrected energy (Eh): -613.38524025

Mode	Frequency (cm^{-1})
6	16.51
7	32.82
8	43.92
9	61.63
10	73.99
11	79.57
12	82.19
13	107.49

14	138.43
15	166.61
16	190.68
17	213.42
18	218.37
19	270.93
20	295.32
21	304.67
22	326.36
23	336.81
24	349.06
25	396.99
26	428.33
27	446.15
28	501.42
29	535.33
30	542.5
31	583.29
32	628.0
33	637.06
34	724.43
35	766.13
36	809.28
37	839.38
38	870.87
39	900.6
40	946.71
41	980.09
42	1017.24
43	1020.48

44	1034.24
45	1053.8
46	1103.52
47	1175.65
48	1190.93
49	1268.24
50	1290.75
51	1321.94
52	1342.99
53	1409.69
54	1514.12
55	1541.9
56	1607.73
57	1625.71
58	1644.3
59	1661.99
60	1667.93
61	1677.78
62	1705.8
63	2148.92
64	2943.87
65	3192.84
66	3199.4
67	3208.35
68	3213.16
69	3222.55
70	3415.7
71	3466.45
72	3541.18
73	3815.65

74	3821.82
75	3868.07
76	3905.55
77	3912.59

Optimised geometry:

C	-1.0897130000	8.5527940000	-1.7019940000
C	-0.8920960000	7.4154330000	-2.4755480000
C	-0.7597810000	6.1676800000	-1.8723750000
C	-0.7996560000	6.0724180000	-0.4856160000
C	-1.0048130000	7.1998750000	0.2969010000
C	-1.1585110000	8.4379840000	-0.3188490000
O	-1.4616080000	9.5693810000	0.3904720000
H	-1.1862900000	9.5298370000	-2.1541940000
H	-0.8302340000	7.5065130000	-3.5509420000
H	-0.5942050000	5.2872930000	-2.4757890000
H	-0.6632370000	5.1120430000	-0.0080170000
H	-1.0400540000	7.1314420000	1.3757410000
H	-1.2012400000	9.4688920000	1.3326620000
O	-3.9067770000	7.1644470000	-1.5254670000
H	-2.9825480000	7.0457170000	-1.8269450000
H	-4.1987220000	6.3668070000	-0.9764440000
H	-3.9897320000	8.0773360000	-0.9912240000
O	-4.0816940000	9.2746460000	-0.2441790000
H	-3.2111670000	9.6052380000	0.0674830000
H	-4.5696830000	10.0150690000	-0.6162220000
O	-4.7448060000	5.1835550000	-0.1405950000
H	-5.4671410000	4.6341750000	-0.4594430000
H	-4.2053410000	4.6314620000	0.4320010000
O	-0.6680220000	9.1900810000	2.9533800000
H	-1.1698340000	9.5035460000	3.7115860000
H	0.2515170000	9.3941740000	3.1499650000

Para-protonated + 1H_2O

Zero-point corrected energy (Eh): -384.10102942

Mode	Frequency (cm^{-1})
6	66.57

7	94.34
8	144.83
9	183.75
10	244.7
11	320.51
12	350.36
13	370.58
14	400.69
15	478.03
16	515.07
17	578.48
18	594.05
19	791.89
20	826.4
21	846.04
22	907.05
23	921.63
24	985.72
25	1013.72
26	1028.9
27	1064.87
28	1111.37
29	1166.73
30	1193.25
31	1210.24
32	1285.99
33	1355.78
34	1383.38
35	1417.81
36	1439.09

37	1542.14
38	1578.91
39	1639.05
40	1648.62
41	1713.11
42	3021.13
43	3036.27
44	3046.31
45	3202.85
46	3206.86
47	3223.41
48	3231.75
49	3804.48
50	3889.39

Optimised geometry:

C	-4.3062980000	2.7197190000	0.0218800000
C	-4.3254160000	1.3776190000	0.0055930000
C	-3.0925620000	0.5682030000	0.0027950000
C	-1.8313300000	1.3338140000	0.0193820000
C	-3.0426920000	3.3969960000	0.0371930000
H	-5.2116790000	3.3096790000	0.0238620000
H	-5.2704670000	0.8510560000	-0.0061620000
H	-3.1013910000	-0.1068170000	-0.8634620000
H	-0.9051180000	0.7749980000	0.0180750000
H	-0.8906850000	3.2452710000	0.0479790000
O	-2.9611980000	4.6792850000	0.0529140000
H	-3.8498130000	5.1473840000	0.0535340000
H	-3.1112000000	-0.1280690000	0.8518740000
C	-1.8073210000	2.6748690000	0.0357060000
O	-5.2769870000	5.8775020000	0.0537650000
H	-5.5294280000	6.4061260000	-0.7106370000
H	-5.5381970000	6.3879460000	0.8275220000

Para-protonated + $2H_2O$

Zero-point corrected energy (Eh): -460.51985810

Mode	Frequency (cm^{-1})
6	28.07
7	50.83
8	70.71
9	98.52
10	134.53
11	145.59
12	157.05
13	182.29
14	235.3
15	240.04
16	280.74
17	330.36
18	355.48
19	358.01
20	397.73
21	475.04
22	516.06
23	581.26
24	596.02
25	799.35
26	827.77
27	844.14
28	909.39
29	919.83
30	988.62
31	1012.77
32	1028.37

33	1072.62
34	1093.85
35	1160.79
36	1184.61
37	1203.98
38	1283.56
39	1341.7
40	1378.01
41	1413.78
42	1439.03
43	1539.73
44	1571.47
45	1631.62
46	1647.32
47	1649.12
48	1709.16
49	3019.38
50	3032.98
51	3108.79
52	3205.05
53	3209.66
54	3225.08
55	3231.35
56	3807.48
57	3819.88
58	3893.42
59	3906.81

Optimised geometry:

C -4.0843000000 2.7400960000 0.3359160000
C -3.9387520000 1.4224990000 0.5623260000

C	-2.6719410000	0.7166250000	0.3142540000
C	-1.5656740000	1.5474180000	-0.1894630000
C	-2.9691670000	3.4844870000	-0.1614590000
H	-5.0142160000	3.2585190000	0.5212030000
H	-4.7509030000	0.8293580000	0.9591770000
H	-2.8544390000	-0.1161280000	-0.3791720000
H	-0.6174370000	1.0636120000	-0.3819130000
H	-0.9046020000	3.4849380000	-0.7849430000
O	-3.0465200000	4.7481280000	-0.4016630000
H	-3.9474690000	5.1409470000	-0.2133500000
H	-2.3874010000	0.1821350000	1.2303360000
C	-1.7058630000	2.8630350000	-0.4149840000
O	-5.4109110000	5.7640300000	0.0964450000
H	-5.9428030000	6.0913700000	-0.6363320000
H	-5.4989050000	6.4160230000	0.7993890000
O	-4.1280020000	-1.0979730000	2.3458370000
H	-4.2613420000	-1.0355940000	3.2959780000
H	-4.3099330000	-2.0195170000	2.1403520000

Para-protonated + $3H_2O$

Zero-point corrected energy (Eh): -536.93748289

Mode	Frequency (cm^{-1})
6	18.34
7	24.57
8	44.73
9	56.25
10	72.99
11	99.9
12	117.06
13	135.59
14	136.2
15	149.14
16	160.04
17	183.91

18	216.87
19	220.91
20	236.27
21	263.51
22	267.79
23	342.44
24	345.94
25	357.92
26	393.25
27	474.75
28	517.79
29	585.77
30	598.58
31	804.22
32	830.1
33	842.47
34	912.71
35	917.84
36	992.24
37	1011.23
38	1029.13
39	1069.88
40	1077.72
41	1159.22
42	1174.43
43	1204.13
44	1282.35
45	1332.83
46	1374.86
47	1410.23

48	1442.46
49	1539.46
50	1564.67
51	1625.08
52	1645.56
53	1647.9
54	1648.38
55	1705.71
56	3018.35
57	3027.84
58	3177.38
59	3210.32
60	3216.99
61	3226.25
62	3232.77
63	3808.99
64	3821.76
65	3822.1
66	3896.71
67	3910.0
68	3910.17

Optimised geometry:

C	-4.1931960000	2.9224200000	-0.3675940000
C	-4.1823420000	1.5849100000	-0.5150230000
C	-3.1396150000	0.7406070000	0.0811060000
C	-2.1028020000	1.4448130000	0.8473750000
C	-3.1535600000	3.5430870000	0.3923990000
H	-4.9571580000	3.5464500000	-0.8090360000
H	-4.9354610000	1.0831550000	-1.1063340000
H	-2.6953520000	0.1233700000	-0.7121160000
H	-1.3435340000	0.8345680000	1.3155840000
H	-1.3571780000	3.3140390000	1.5583940000

O	-3.1127810000	4.8224980000	0.5686970000
H	-3.8662130000	5.3092280000	0.1327390000
H	-3.5807440000	-0.0361470000	0.7214600000
C	-2.1082970000	2.7812760000	0.9947530000
O	-5.1005770000	6.1053440000	-0.5929310000
H	-4.8985730000	6.7192030000	-1.3063340000
H	-5.7618200000	6.5428330000	-0.0471330000
O	-2.1409710000	-1.3719530000	2.1385220000
H	-1.7418980000	-2.2081670000	1.8823560000
H	-2.2725650000	-1.4514180000	3.0875550000
O	-4.2149550000	-0.9317270000	-2.3183530000
H	-4.5909440000	-1.8047100000	-2.1743990000
H	-4.0883580000	-0.8805880000	-3.2699630000

Para-protonated + 4H_2O

Zero-point corrected energy (Eh): -613.35514668

Mode	Frequency (cm^{-1})
6	-8.33
7	19.05
8	23.5
9	24.45
10	47.31
11	59.63
12	76.04
13	95.56
14	98.14
15	127.79
16	134.69
17	147.34
18	161.13
19	172.39
20	185.78
21	208.22

22	222.19
23	231.24
24	235.76
25	263.57
26	275.12
27	314.67
28	336.41
29	353.23
30	385.4
31	413.55
32	472.7
33	516.88
34	589.36
35	596.38
36	616.43
37	808.13
38	830.82
39	844.97
40	912.32
41	926.25
42	986.14
43	1009.57
44	1033.29
45	1071.29
46	1081.67
47	1157.15
48	1180.93
49	1201.16
50	1277.28
51	1349.36

52	1373.53
53	1407.7
54	1436.87
55	1542.1
56	1563.24
57	1628.6
58	1636.97
59	1642.88
60	1647.84
61	1654.78
62	1706.63
63	3015.95
64	3035.86
65	3166.07
66	3216.08
67	3223.45
68	3231.77
69	3235.48
70	3721.03
71	3814.55
72	3817.95
73	3821.64
74	3898.09
75	3904.35
76	3907.02
77	3909.74

Optimised geometry:

C	-4.3490440000	2.4787120000	-0.0189880000
C	-4.2874290000	1.1441820000	0.1367410000
C	-3.0742220000	0.4597850000	0.6066900000

C	-1.9269800000	1.3310270000	0.9136690000
C	-3.1973390000	3.2610130000	0.2922320000
H	-5.2446650000	2.9529240000	-0.3936460000
H	-5.1568920000	0.5542500000	-0.1139490000
H	-2.7790930000	-0.2846070000	-0.1464790000
H	-1.0360530000	0.8491480000	1.2907030000
H	-1.1566480000	3.3177010000	0.9890390000
O	-3.1926350000	4.5461500000	0.1613490000
H	-4.0640290000	4.9121060000	-0.1586930000
H	-3.2797100000	-0.1691440000	1.4823630000
C	-1.9861620000	2.6641230000	0.7640650000
O	-5.5038640000	5.5071330000	-0.6430100000
H	-5.6606350000	5.7660770000	-1.5559890000
H	-5.9821250000	6.1389590000	-0.0976680000
O	-1.4773860000	-1.1207680000	2.7711130000
H	-1.1347610000	-2.0098460000	2.6436580000
H	-1.3874100000	-0.9641660000	3.7153950000
O	-4.8612560000	-0.4562390000	-2.6858420000
H	-5.1713130000	-1.3565960000	-2.8226670000
H	-4.3788980000	-0.2388870000	-3.4891540000
O	-6.7162460000	1.4950080000	-1.6623550000
H	-6.2501660000	0.8428970000	-2.2048040000
H	-7.6512800000	1.3364500000	-1.8091710000

Para-protonated + 10H_2O

Zero-point corrected energy (Eh): -1071.89871188

Mode	Frequency (cm^{-1})
6	5.13
7	14.04
8	16.67
9	21.81
10	39.17
11	41.79
12	48.74
13	51.89
14	61.21

15	66.58
16	79.26
17	81.79
18	86.49
19	93.94
20	102.9
21	110.61
22	121.5
23	135.26
24	150.24
25	159.46
26	168.32
27	176.26
28	197.31
29	200.19
30	206.27
31	213.19
32	218.24
33	233.2
34	239.71
35	243.2
36	255.37
37	273.25
38	279.21
39	296.1
40	298.81
41	303.64
42	308.36
43	315.89
44	321.58

45	327.53
46	331.56
47	357.99
48	364.9
49	399.31
50	403.97
51	413.59
52	426.76
53	455.22
54	499.89
55	513.14
56	518.33
57	527.55
58	536.78
59	567.0
60	571.19
61	578.88
62	590.79
63	601.78
64	617.42
65	652.43
66	673.99
67	697.93
68	769.98
69	787.6
70	814.19
71	828.41
72	843.36
73	889.27
74	905.33

75	910.63
76	918.66
77	984.58
78	989.02
79	1018.7
80	1022.9
81	1056.86
82	1166.47
83	1186.53
84	1202.84
85	1211.97
86	1323.71
87	1350.43
88	1403.32
89	1414.26
90	1479.07
91	1550.69
92	1606.64
93	1627.8
94	1635.68
95	1635.84
96	1644.59
97	1648.42
98	1653.93
99	1656.28
100	1661.13
101	1664.9
102	1683.9
103	1711.26
104	1713.24

105	2385.89
106	3023.34
107	3061.39
108	3103.55
109	3199.87
110	3220.61
111	3229.0
112	3247.01
113	3375.86
114	3520.51
115	3557.56
116	3562.03
117	3574.53
118	3599.82
119	3618.94
120	3667.22
121	3685.85
122	3783.68
123	3866.88
124	3880.56
125	3884.78
126	3885.69
127	3886.76
128	3888.58
129	3892.99
130	3895.54
131	3903.98

Optimised geometry:

C -3.8916120000 2.4998060000 0.4939720000
C -4.0509730000 1.1749480000 0.3523720000

C	-2.9206740000	0.2330810000	0.3521790000
C	-1.5986000000	0.8428020000	0.5829400000
C	-2.5744730000	3.0262360000	0.6525870000
H	-4.7357320000	3.1707900000	0.5019280000
H	-5.0478520000	0.7782560000	0.2225970000
H	-2.9170920000	-0.2930140000	-0.6117480000
H	-0.7411780000	0.1840790000	0.6202850000
H	-0.4633840000	2.6195940000	0.8723350000
O	-2.3591450000	4.2962270000	0.7077330000
H	-3.2226120000	4.8643500000	0.5860600000
H	-3.0989190000	-0.5420320000	1.1054100000
C	-1.4318650000	2.1641640000	0.7292390000
O	-4.4606060000	5.5676250000	0.2541270000
H	-4.7041860000	5.2615000000	-0.6672170000
H	-4.5410280000	6.5232260000	0.2911580000
O	-3.4127140000	0.3794480000	3.4501200000
H	-3.1117940000	-0.1203290000	4.2129870000
H	-3.1446710000	1.3030030000	3.6070850000
O	-3.9605690000	0.7583220000	-2.7511850000
H	-3.9394670000	0.1480550000	-3.4927460000
H	-3.4462420000	1.5403500000	-3.0315910000
O	-3.4761850000	3.1603640000	3.4427400000
H	-4.4285520000	2.9634140000	3.3613730000
H	-3.3883790000	3.7704710000	4.1789080000
O	-5.7997120000	1.7590550000	2.9991520000
H	-6.6356010000	1.6538590000	3.4593370000
H	-5.2440850000	1.0035440000	3.2530870000
O	-6.2229290000	2.0462180000	-1.7808560000
H	-7.0688990000	1.8608860000	-2.1956290000
H	-5.5657230000	1.4729170000	-2.2181730000
O	-2.7007880000	3.1768210000	-3.3088020000
H	-2.6478400000	3.5000000000	-4.2126290000
H	-1.7877050000	3.2371660000	-2.9425730000
O	-0.2491650000	3.1962260000	-2.2338360000
H	0.5268710000	2.9010450000	-2.7147690000
H	0.0652050000	3.8274870000	-1.5599180000
O	-4.9143050000	4.4759930000	-2.0535780000
H	-4.1073520000	4.1379340000	-2.4737470000
H	-5.5018300000	3.6984120000	-1.9866960000
O	0.3754850000	4.7990490000	-0.0885290000
H	-0.4594140000	5.0361130000	0.3324270000
H	0.9632870000	5.5511090000	0.0160420000
